# Using Single-Voxel Magnetic Resonance Spectroscopy Data Acquired at 1.5T to Classify Multivoxel Data at 3T: A Proof-of-Concept Study

**DOI:** 10.3390/cancers15143709

**Published:** 2023-07-21

**Authors:** Gülnur Ungan, Albert Pons-Escoda, Daniel Ulinic, Carles Arús, Alfredo Vellido, Margarida Julià-Sapé

**Affiliations:** 1 Centro de Investigación Biomédica en Red (CIBER), 28029 Madrid, Spain; gulnur.ungan@autonoma.cat (G.U.); carles.arus@uab.cat (C.A.); avellido@lsi.upc.edu (A.V.); 2 Departament de Bioquímica i Biologia Molecular and Institut de Biotecnologia i Biomedicina (IBB), Universitat Autònoma de Barcelona (UAB), 08193 Barcelona, Spain; daniel.ulinic@uab.cat; 3 Group de Neuro-Oncologia, Institut d’Investigació Biomèdica de Bellvitge (IDIBELL), Hospital Universitari de Bellvitge, 08908 Barcelona, Spain; albert.pons@bellvitgehospital.cat; 4 IDEAI-UPC Research Center, UPC BarcelonaTech, 08034 Barcelona, Spain

**Keywords:** magnetic resonance spectroscopy, brain tumors, glioblastoma, decision support systems, nosologic imaging, metabolic pattern

## Abstract

**Simple Summary:**

One of the main applications of in vivo magnetic resonance spectroscopy (MRS) is in the non-invasive monitoring of the metabolic pattern of brain tumors. MRS comes in two basic modalities, single-voxel (SV), from which the signal is obtained, and multivoxel (MV), in which one or more contiguous grids of SVs are acquired. The purpose of our proof-of-concept study was to test whether it would be possible to train machine learning models using SV data at 1.5T, and test them with MV 3T data from independent patients, obtaining color-coded images of pathology (nosological images) to help radiologists in their preoperative evaluation of patients. With sequential forward feature selection followed by linear discriminant analysis, we obtained AUCs = 0.95 (meningioma), 0.89 (aggressive), 0.82 (low-grade glioma), and 0.82 (normal brain) in the MV test set.

**Abstract:**

In vivo magnetic resonance spectroscopy (MRS) has two modalities, single-voxel (SV) and multivoxel (MV), in which one or more contiguous grids of SVs are acquired. Purpose: To test whether MV grids can be classified with models trained with SV. Methods: Retrospective study. Training dataset: Multicenter multiformat SV INTERPRET, 1.5T. Testing dataset: MV eTumour, 3T. Two classification tasks were completed: 3-class (meningioma vs. aggressive vs. normal) and 4-class (meningioma vs. low-grade glioma vs. aggressive vs. normal). Five different methods were tested for feature selection. The classification was implemented using linear discriminant analysis (LDA), random forest, and support vector machines. The evaluation was completed with balanced error rate (BER) and area under the curve (AUC) on both sets. The accuracy in class prediction was calculated by developing a solid tumor index (STI) and segmentation accuracy with the Dice score. Results: The best method was sequential forward feature selection combined with LDA, with AUCs = 0.95 (meningioma), 0.89 (aggressive), 0.82 (low-grade glioma), and 0.82 (normal). STI was 66% (4-class task) and 71% (3-class task) because two cases failed completely and two more had suboptimal STI as defined by us. Discussion: The reasons for failure in the classification of the MV test set were related to the presence of artifacts.

## 1. Introduction

In vivo magnetic resonance spectroscopy (MRS) can non-invasively capture biochemical information from free metabolites in the millimolar (mM) range of concentration from living organisms such as humans. It does not use ionizing radiation and the only inconveniences for patients, at the magnetic fields normally used in the clinic (1.5T or 3T), are possible claustrophobia and the long acquisition times inside a noisy scanner. MRS comes in two main modalities, single-voxel (SV) and multivoxel (MV). In SV, one volume (basically a cube with edges of 1–2 cm) is excited to obtain the signal from the MR-visible metabolites. In MV, one or more grids of contiguous SVs are acquired. MV is also known as “spectroscopic imaging” or, sometimes, as chemical shift imaging (CSI). 

The MRS technique has been used for about 30 years and is still considered to be a promising one, but clinicians scarcely use it in their daily practice. Reasons for this are mainly related to the lack of a “killer app” that offers a distinctive advantage over alternatives. MRS is best suited for magnetically homogeneous tissues, such as brain tissue, and indeed a great deal of MRS applications concern brain diseases. In brain tumors, for example, the spectral pattern undergoes drastic changes depending on the tumor type and grade [1]; therefore, it has been the target for landmark studies that have shown its value in terms of prediction of type [2,3,4], infiltration [5,6,7,8], or relationship of the pattern with prognosis [9,10,11]. Despite this, clinicians still struggle with the export of MRS data from scanners, while scientists struggle with the access to data and hidden proprietary DICOM tags, as data sharing has never been customary in the MRS area. Only recently has the MRS community engaged in efforts to establish a consensus for acquisition, processing parameters and reporting [12,13,14,15], or to share datasets through the recently created MRShub platform [16]. Other factors influencing the advancement of applications based on in vivo MRS are related to the progress of the technique itself, in terms of technical capabilities, the increase in magnetic field that allows for better resolution, and the development of improved sequences, such as semi-Laser [12,17], which provide better data quality. In the early period of MRS, a magnetic field of 1.5T was the norm, while nowadays the clinical routine involves 3T scanners in many centers, with 7T scanners becoming more and more common in top institutions. Apparently, such heterogeneity is not a problem, because MRS data are not routinely shared. This creates a difficult challenge: how do we train machine learning models to analyze these data, if it will take years to recruit the necessary patients to feed data into prediction models? Should we throw away all the previous data and knowledge gained throughout the years with SV acquired at lower fields? Recently, deep learning has begun to be used in MRS to develop automated quality control procedures to detect artifactual patterns in MRS. As deep learning is very data-intensive and there are no easily accessible MRS datasets with a sufficient amount of data to appropriately train these models, one frequent solution has been to resort to synthetically generated sets [18,19,20].

The work we present here is a proof of concept for the feasibility of stored MRS data reuse for machine learning, as applied to brain tumor diagnosis. Our hypothesis is that it is possible to train machine learning models using 20–30-year-old 1.5T SV data and apply the best model to classify and color-code these data in the form of nosological images’ “not-so-old” MV data (from 2006 to 2009) to achieve metabolic-based segmentations of the most common brain tumor types that match the solid tumor region and identify whether the tumor is infiltrative or not. 

For this, we have used retrospective, multicenter curated MRS data from the largest existing SV database to train a machine learning model that will help us classify new MV data. Each voxel in each MV (single- or multi-slice) will be classified and assigned a color according to the probability of belonging to each of the classes of our classifier (e.g., normal brain in blue, malignant tumors in red, etc.). Then, a color-coded image of the limits of the tumor, as well as the tumor type, will be generated, just as a mosaic image is the aggregate result of thousands of tiles of different colors.

We will demonstrate that this is indeed possible, not only using multicenter SV data to train and to test MV data, but also changing the magnetic field, as the SV were acquired at 1.5T, whereas the MV that we used were acquired at 3T, and with a different sequence (PRESS and STEAM for SV and semi-Laser for MV) and at a different clinical center. We will also show that, when our classifier failed on the MV data, it was due to the presence of artifacts.

This work has been possible with the reuse of two databases that our research group has been curating after the end of the funding period, namely the INTERPRET and the eTUMOUR databases.

INTERPRET was a European multicenter study carried out twenty years ago (IST-1999-10310, from January 2000 to December 2002) that collected MRS data from eight magnetic resonance centers in five countries [21,22]: CDP (Centre Diagnòstic Pedralbes-CETIR, Units: Pedralbes, Barcelona and Esplugues del Llobregat, Spain), IDI (Institut de Diagnòstic per la Imatge-Unitat Bellvitge, L’Hospitalet del Llobregat, Spain), SGUL (St George’s University of London, United Kingdom), UMCN (Universitair Medisch Centrum Nijmegen, The Netherlands), UJF (Unité Mixte Université Joseph Fourier/INSERM U594, Grenoble, France), FLENI (Fundación para la Lucha contra las Enfermedades Neurológicas de la Infancia, Buenos Aires, Argentina), and MUL (Uniwersytet Medycznyw Lodzi, Lodz, Poland). The second database was obtained from the eTUMOUR project (Web Accessible MR Decision Support System for Brain Tumour Diagnosis and Prognosis, incorporating in vivo and ex vivo Genomic and Metabolomic Data), an EU-funded effort (FP6-2002-LIFESCIHEALTH 503094), carried out in the 2004–2009 period, that involved 21 partners across Europe and Argentina. One of its results was the eTumour database (eTDB) [23].

## 2. Materials and Methods

**Figure 1:**
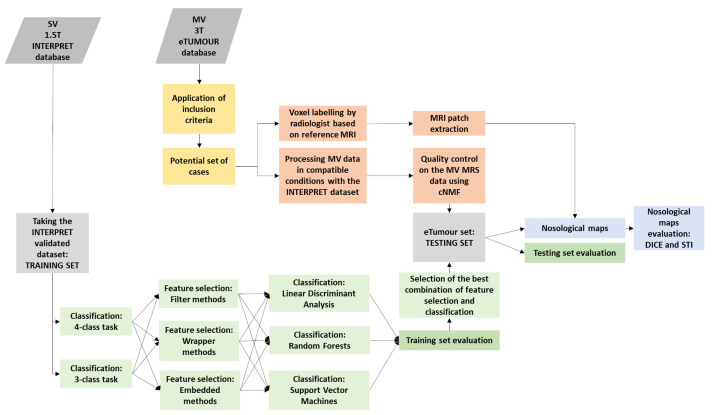
Summary of the pipeline.

A summary figure of the pipeline used in the methodology is displayed in Figure 1.

### 2.1. Datasets

The study was retrospective. Two short TE datasets were used, one for SV and one for MV. The SV one was the INTERPRET-validated dataset. For the current study, we used the short TE dataset only from the so-called INTERPRET-validated database [21,22,24], which is composed of 304 single-voxel (SV) spectra acquired from three different brands of 1.5T MR scanners, namely GE Signa Advantage and LX CV/i 1.5T, Philips NT and ACS NT 1.5T, and Siemens Vision 1.5T, from the most common tumor types and from 22 normal volunteers, as has been described previously [21,22,24]. Each spectrum belonged to one patient, and was validated at multiple levels, ensuring that the spectral quality was good, that each spectrum had been acquired from the cellular part of the tumor avoiding cysts or edematous regions and from the same region where the diagnostic biopsy sample had been taken, and that the histopathological diagnosis had been agreed upon among a panel of expert pathologists. All studies were performed in accordance with the medical ethics and regulations of the countries concerned and all patients or their legal representatives signed informed consent forms, agreeing to the study or the use of anonymized data for research. This dataset had been acquired between the years 2000–2002; therefore, the version of the histopathological classification used by pathologists was the reference one at the time, namely the WHO classification of brain tumors of the year 2000 [25,26]. This dataset has been extensively used in independent studies during the last 20 years, for machine learning applications to MRS (e.g., in [24,27,28,29,30,31,32,33,34,35,36]). Table 1 summarizes the acquisition conditions for the SV dataset. For the current study, only the short TE data from the INTERPRET database are used. 

In the in vivo MRS field, it is necessary to process the raw data in the time domain as it comes from the MR scanner. Steps involve applying signal processing algorithms such as the Fourier transform to convert the signal into the frequency domain, water signal suppression, and phasing and/or apodization, which are among the most common steps [12,37]. The processing tool and parameters used for this work were those used in the INTERPRET project and the Data Manipulation Software (DMS) [21,24,38,39], which provides a 512-point spectrum in the [−2.7, 7.1] ppm frequency interval, normalized to unit length (UL2), and with the [4.2, 5.1] ppm region zeroed so that any remnant unsuppressed water does not interfere with the UL2 peak heights of the relevant metabolite signals. Otherwise, the INTERPRET parameters involved water suppression with Hankel Lanczos Singular Value Decomposition (HLSVD) using 10 Lorentzians in the [4.31, 5.11] ppm region. This specific parametrization has the advantage of unifying different spectral ranges and number of points found from slightly different acquisition conditions [12], in particular during the late 1990s and early 2000s, in which the DICOM standard did not exist for MRS data. The so-called 1.5T SV short TE dataset, then, was processed exactly using the INTERPRET parameters with manual realignment. This exact data matrix has been used in several studies [24,27,36] and in all versions of the INTERPRET decision support system [39]. The SV data were converted into XML files using SpectraClassifier version 1.0 [28].

The 304 spectra data matrix contains 35 low-grade glial tumors (*lgg*) comprising 22 astrocytomas (*a2*), 6 oligoastrocytomas (*oa*), and 7 oligodendrogliomas (*od*) of WHO 2000 Grade II; 123 aggressive tumors (*agg*) comprising 85 glioblastoma (*gb*), 38 metastasis (*me*), and 62 low-grade meningiomas (*mm*), including WHO 2000 Grades I and II. The rest of the cases in the dataset correspond to 22 normal (*no*) volunteers (normal-appearing white matter, NAWM), 10 malignant brain lymphomas (*ly*), 8 abscesses (*ab*), and 44 other cases from 17 different pathologies and/or grades. 

eTDB: The eTUMOUR data used for this study are multivoxel (MV). Since no previously processed MV data matrix from the eTDB exists, the database was queried with the following inclusion criteria:

1. The MV is stored as a valid experiment in the eTDB (not marked as test case).
2. Acquisition was done using PRESS or semi-Laser sequences.
3. Echo time (TE) is short TE (30–32 ms).
4. The diagnosis of the case is *mm*, *gb*, *me*, *lgg* (*a2*, *od* or *oa*).
5. The MRI study must be fully loaded into the eTDB, i.e., the whole set of images must be uploaded; therefore, the number of MRI slices could not be lower than the number of MRS slices.
6. For multi-slice MV acquisitions, the number of MRS slices had to be the same as the available MRI slices.
7. The data format should allow for the extraction of the parameters for MV grid localization over the corresponding MRI slice.

MV data fulfilling the inclusion criteria were downloaded and processed in the same way as the SV data, as it was crucial for this work to have the MV data processed in the same way, with the same normalization, spectral range, and number of points. To this end, processing was completed using the jMRUI2XML [40] plugin of jMRUI version 6.0 [41], which can output data exactly in the same format as the SV INTERPRET processed the data. For this, the plugin was run with the INTERPRET parameters, except that the order of priority in the alignment correction was 2.01 ppm first, then 3.21 ppm, and then 3.03 ppm. Given that, at the time of processing, it was noticed that MV grids had individual spectra that were flipped downwards (requiring 180 degree zero order correction), the person processing the data (GU) also performed an additional phase correction with the jMRUI menu on each individual spectrum that was visually observed to be flipped down before exporting. After performing these final corrections, each grid composed of n x m 512-point spectra on the [−2.7, 7.1] ppm range were exported as XML files with the above-mentioned jMRUI plugin. 

Both SV and MV data were processed offline with Excel (version 2021) to only extract the [0, 4.2] ppm range, to account for the remaining effects of the incomplete water suppression processing pipeline, and to renormalize UL2 in that range and re-export it as XML for all classification experiments. 

### 2.2. MRI Processing of the eTDB Data

From the available reference MR images of the eTDB cases, patches corresponding to the same location as the MV grid were extracted using Gannet [42], obtaining one MRI patch per MV slice. 

These patches were used for two different purposes: first, to label the anatomical regions detected such as ventricles, oedema, cysts, and unaffected brain tissue; second, to overlay the nosological images (the color maps obtained after classification of each individual voxel) onto the anatomical patches. 

### 2.3. MV Voxel Labelling

The multivoxel grid was co-registered to morphological MR images for each patient. Then, a neuroradiologist with 10 years of experience in neuro-oncology (A.P.) classified each individual voxel as: solid tumor region, abnormal tumor region (oedema or cysts), normal tissue, or ventricles. This voxel classification was carried out manually and was based on the expert neuroradiological evaluation of the underlying T2WI, FLAIR, or CE-T1WI available for each patient. The voxels labelled as ventricle were excluded from the analysis. 

### 2.4. Quality Control

SV data were considered as good quality because the data matrix was the same one that had been used in multiple previous studies, with the spectra having passed all the quality controls that have already been mentioned. Therefore, no additional quality control steps were applied.

MV-processed data still contained a variety of artifacts, such as low signal-to-noise (SNR) ratio, flipped-down spectra, or poor water suppression. Individual spectra with SNR values lower than 10 in the [−2.7, 7.1] ppm range were discarded. Afterwards, an approach based on extracting artifactual patterns using convex non-negative matrix factorization (cNMF) was applied [43]. We extracted from 2 to 5 sources to identify artifactual patterns, and if the highest contribution was from an artifactual pattern, then the individual spectrum was discarded from the analysis. 

### 2.5. Classification Tasks

Classifiers were trained with the SV data (training set) and tested with the MV data (test set). Two classification tasks were performed:

- 4-class task: *mm* vs. *lgg* vs. *agg* vs. *no*
- 3-class task: *mm* vs. *agg* vs. *no*.

### 2.6. Feature Selection

Feature selection was performed on the SV data. Five different algorithms from three different methods were used to extract from 3 to 20 features:

- Filter methods: Chi [44].
- Wrapper methods: Sequential Forward Feature Selection (SFFS) [45], Boruta Feature Selection [46], and Select k-best [47].
- Embedded methods: Lasso Feature Selection [48].

For each feature selection algorithm, mutually correlated features were discarded using Pearson’s correlation [49], with the additional criterion of discarding the left (higher ppm value) feature if the correlation between the left and the right features was >80%. For Boruta feature selection, we applied Pearson’s correlation with different threshold values as 50%, 60%, 70%, 80%, and 90%. 

Shapley values were used to explain the global importance of each feature in the model and were calculated for LDA by using the Shap library [50].

### 2.7. Classification

The following classification methods were used: Linear Discriminant Analysis (LDA) as implemented in SpectraClassifier [28], Random Forest (RF) [51], and Support Vector Machines (SVM) [52] implemented in Python version 3.7.9.

### 2.8. Classifier Evaluation

Classifier evaluation was completed on the training and test sets. Each classification process was repeated 1000 times with bootstrapping on the training data set. Balanced error rate (BER) and area under the ROC curve (AUC) were calculated for the training and test sets. The test set validation was completed using the MV spectra from the solid tumor and the normal regions because the class labels (the tumor type) were the same as in the training set. The solid tumor regions of the MV set corresponded to the SV solid tumor regions. The normal regions of the MV dataset corresponded to the *no* class in the SV training set—these SV were acquired over the white matter of normal volunteers. 

The criteria for choosing the best classifier were that the AUCtrain and the AUCtest should be as close to 1 as possible, and that the AUCtest/AUCtrain ratio should be closest to 1. Furthermore, the BER, BERtrain, and BERtest values should be as close to 0 as possible, while the BERtest/BERtrain ratio should be closest to 1.

### 2.9. Visualization of the Test Set Classification: Nosological Maps

After obtaining the best classifier, we used it to obtain the nosological images of each MV case with the SpectraClassifier 3.0 tool [28]. Briefly, SpectraClassifier MV tab plots each individual voxel in a color chosen by the user, whose intensity represents the probability of the winning class. The colors chosen for each of the classes were: blue for normal brain (*no*), red for the aggressive class (*gb* and *me*), green for the *lgg,* and yellow for *mm*. A Power-BI tool was implemented for the nosological image representation. The excluded voxels were shown as black voxels with 0.5 transparency. As mentioned previously, two types of voxels were excluded: those that were in the ventricle areas as marked by the radiologist, and the ones discarded after cNMF analysis for being artifactual. A third step for nosological image display and analysis was taken, in which the SNR <10 spectra were also excluded.

### 2.10. Nosological Image Evaluation

The following aspects were evaluated:

- Whether the class of the solid tumor region corresponded to the diagnosis of the patient.
- Whether the localization of the solid tumor region agreed with the MRI segmentation performed by the radiologist.
- Whether it agreed with the MRI segmentation (in cases where the radiologist marked a surrounding, abnormal area).

The evaluation measures were: BER for each grid and AUC for each class of each grid. It was noted that, for patients with a multi-slice acquisition, each grid had a different distribution of classes according to the radiologist’s segmentations. The Dice score was also calculated for each class of each grid, to see how well MV segmentation matched the radiologist’s segmentations.

The accuracy in the prediction of the class of the solid tumor region was computed for the test set using AUC and with the following Solid Tumor Index (STI):

$$\mathrm{STI}=\frac{\mathrm{number}\;\mathrm{of}\;\mathrm{correctly}\;\mathrm{classified}\;\mathrm{voxels}}{\mathrm{total}\;\mathrm{number}\;\mathrm{of}\;\mathrm{voxels}\;\mathrm{in}\;\mathrm{the}\;\mathrm{solid}\;\mathrm{tumor}\;\mathrm{region}\;-\;\mathrm{excluded}\;\mathrm{voxels}\;\mathrm{by}\;\mathrm{low}\;\mathrm{quality}}$$

## 3. Results

### 3.1. Available Data

A total of 85 cases in the eTDB fulfilled inclusion criteria 1–4 (valid experiment, PRESS or similar, short echo time, and belonging to the tumor classes). Applying criterion 5, there were 44 cases. Applying 6 and 7 resulted in a final set of 17 usable cases (Supplementary Materials A). There were 4 *mm*, 8 *gb*, 2 *me*, 1 *lgg*, and 2 anaplastic glial (oligodendroglioma and oligoastrocytoma) (Supplementary Materials A). Then, the total number of cases used for the classification tasks was 15 (4-class task) and 14 (3-class task) as the anaplastic intermediate malignancy grades (WHO Grade III in the 2000 and 2007 classifications) do not belong to any of the classes of the tasks that we established. 

Causes for the exclusion of cases were mainly due to the old formats of data, e.g., 3 cases from *Universidad de Valencia* were in Philips old Gyroscan format (SDAT/SPAR), in which the grid localization was not possible with the available parameters and current software programs; 29 cases *from St. Georges University of London* (SGUL) with GE format Probe 8x or SAGE IDL whose parameters could not be read for co-registration; 1 case from *Medizinicz Lodz* that did not have a companion MRI; and 2 cases from *University Center Nijmegen* (UMCN) and 5 cases from Cambridge University that, although they were in Siemens *Numaris* 4 RDA format, had an uneven number of MRI slices with respect to the number of MV slices in the eTDB. A final set of 17 cases from UMCN were included in the study, of which the most prominent characteristics were that they had been acquired using the semi-Laser sequence [53,54] at a TE of 30 ms on a 3T scanner. The rest of relevant characteristics are shown in Table 2. In total, 6 of the 17 cases were multi-slice with a z dimension = 8, with a range of four different VOI geometries, while the other 11 were single-slice, with a range of 10 different VOI dimensions, from 18 to 25 for the x dimension and 14 to 24 for the y dimension (Table 2 and Supplementary Materials A).

In total, 59 MV slices were included in the analysis, containing 8452 individual voxels inside the VOI, of which 812 (2.5%) were discarded by the radiologist because they were placed inside the brain ventricles, making a final total of 7640 voxels available for further analysis.

All 17 cases (including the two anaplastic ones) were used for the visualization with nosological images. 

**Figure 2:**
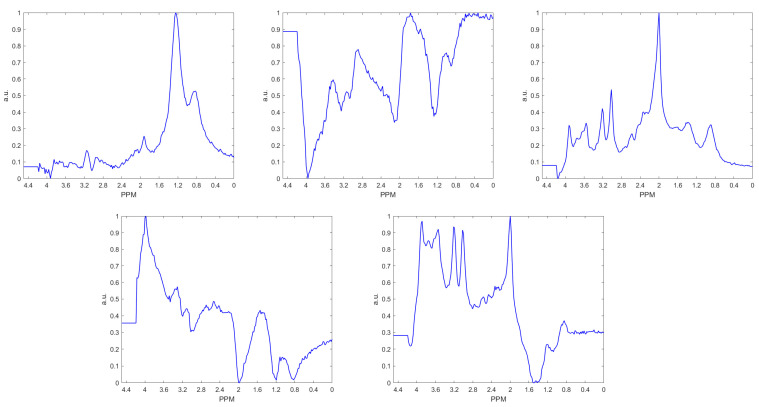
Sources obtained via cNMF on MV data. First row, left to right: source 1, source 2, and source 3. Second row, left to right: source 4 and source 5. Source 1 was the majority contribution in 1245 spectra, source 2 (considered artifactual) in 273 spectra, source 3 in 3685 spectra, source 4 (considered artifactual) in 623 spectra, and source 5 in 1814 spectra.

With respect to the QC procedure, Figure 2 shows the sources obtained after performing cNMF. Source 1 represents a typical necrotic pattern, with high lipids at 0.9 and 1.28 ppm; source 3 represents a typical normal brain pattern, with NAA-containing compounds at 2.01 ppm being the highest peak; and source 5 can be considered partly artifactual (high baseline and inverted peaks around 1.5 ppm, presumably due to low homogeneity or other causes, but still interpretable, with a low 2.01 ppm peak, attributable to areas of neuronal loss). The other two sources (2 and 4) were considered artifactual by the expert spectroscopists (CA and MJS). As can be observed, the so-called sources 2 and 4 correspond to flipped-down spectra. The explanation for finding this type of artifact was that the member of the team conducting the visual inspection of the 8452 spectra, the class labelling, and the manual correction of the flipped-down spectra (GU) missed part of them due to eyestrain. Therefore, an important proportion of the flipped-down spectra were still present in the testing dataset. Nevertheless, the team decided to be conservative and only exclude the clearly flipped down spectra, which was when the winning source contribution was either number 2 or number 4. From the 7640 spectra inside the VOI, but not on ventricles, the winning source was number 1 in 1245, number 2 in 273, number 3 in 3685, number 4 in 623, and number 5 in 1814 spectra; therefore, 896 individual spectra were discarded through this procedure. The quality control procedure discarded entire slices from the multi-slice cases.

### 3.2. Feature Selection and Classification Results

For both classification tasks, the best results were obtained with the combination of SFFS and LDA (Table 3 and Table 4), therefore we used these results for nosological image visualization. 

Regarding the performance of the different feature selection and classification methodologies, as expected, the BER for the MV test set was higher than for the SV training set. 

For the 4-class task (Table 3), all methods performed similarly in the training set, except Chi feature selection with any classification method (mean BER ranging from 0.21 to 0.33). Chi failed particularly between six and eight features; also, SFFS combined with RF, the worst classifier with six features. K-best also did not perform well at a low number of features (three features), combined with LDA and SVM classifiers. In the test set, the best results were obtained for LDA classification, combined with SFFS, K-best, or Boruta. 

For the 3-class task (Table 4) in the SV training set, again, SFFS had the lowest performance with SVM and RF below six features. Chi also performed poorly with any classifier, particularly at five features both with the SV training and the MV test set, no matter the classification method. In the MV test set, the best combinations were again LDA with SFFS, Lasso, and Boruta, according to the maximum, minimum, and mean BER values obtained. 

Therefore, given the criteria set for choosing the best classifier, the best combination was SFFS followed by LDA both for the 4-class task and the 3-class task, using eight and nine features, respectively. 

**Figure 3:**
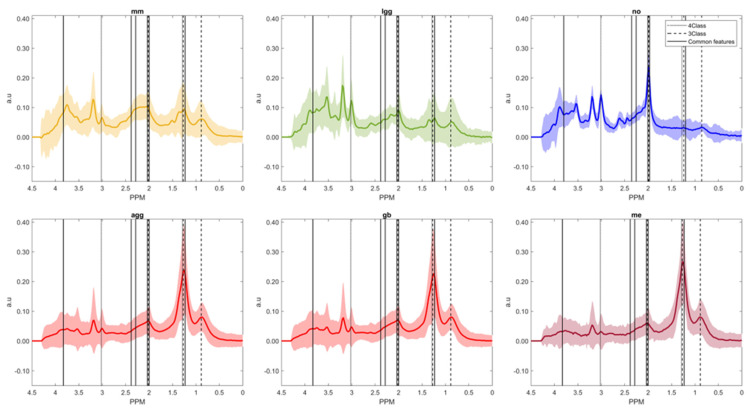
Mean and standard deviation of the different classes (*mm*, *lgg, no*, *gb*, *me,* and the superclass *agg*) in the training set (SV), as well as features selected with the SFFS method, for the 4-class task (continuous vertical lines), the 3-class task (dashed vertical lines), and for both tasks (thick continuous vertical line).

**Figure 4:**
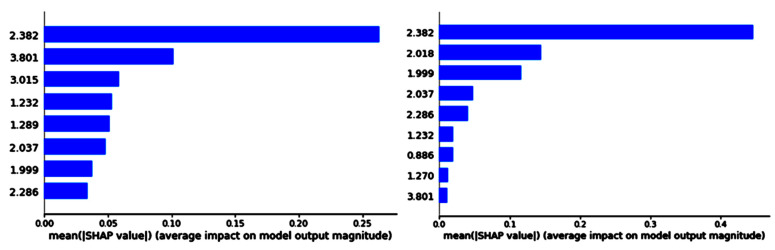
Shapley values of the better combinations for 4 and 3 classes. Left, 4 classes; right, 3 classes. *X*-axis is the selected feature’s ppm values and *Y*-axis is the Shapley values.

In Table 5, a summary of the main classifier performance values of the chosen combination of feature selection and classifier is shown. Figure 3 and Figure 4 show the feature importance for each of the two tasks, in the SFFS/LDA combination, where the most important feature in both tasks was 2.382 ppm, which could be consistent with Glx, macromolecules, or mobile lipids.

### 3.3. Visualization Results

Detailed results on all the measures were taken, case by case, for each of the 49 grids, and the two classification tasks are shown in Supplementary Materials B and C. 

**Figure 5:**
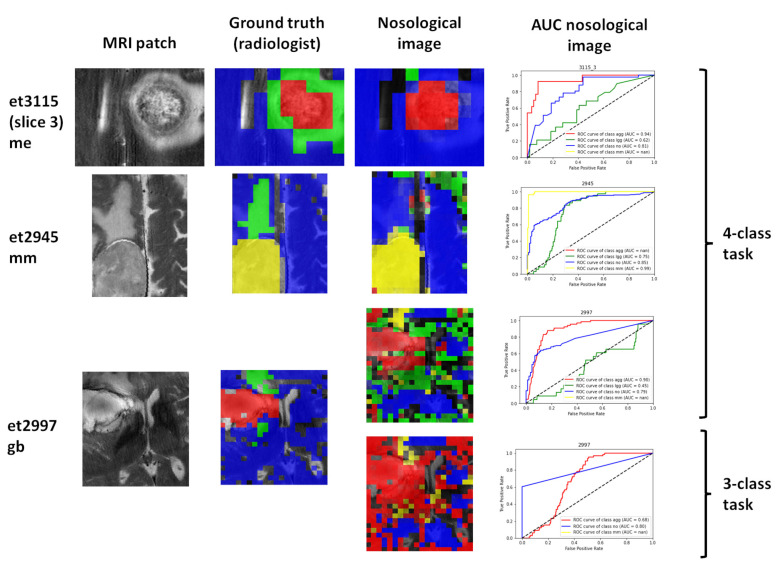
Three example cases. Rows: cases. Columns: MRI patch or MRI that corresponds to the same location as the MV MRS slice. Ground truth is the segmentation carried out by the radiologist: blue, normal brain; red, solid tumor region in *gb* or *me*; yellow, solid tumor region in *mn*; green, abnormal region (oedema, other). In the nosological image on the third column, the color codes are the same, except that the green represents *lgg*. The fourth column shows the bilateral AUC values for each case. The keys on the right side indicate the classification tasks.

Figure 5 displays three characteristic cases. The most relevant finding upon qualitative evaluation of the whole test set was that, for most cases, the solid tumor region belonged to the same class of the tumor the patient had. Also, in *gb*, the surrounding abnormal region identified by the radiologist was classified as *lgg* when the 4-class task was applied, and in some cases extended further (Figure 5, case et2997). When the 3-class task was applied, areas that had been classified as *lgg* were classified either as *gb* (Figure 5, case et2997) or as mm. We can explain that as follows: the classifier would predict the closest label to the data, depending on the classes it was trained to recognize. Not surprisingly, *lgg* predictions for the abnormal surrounding area of a *gb* are in line with the knowledge on this kind of tumor, which is infiltrative. In contrast, for *me* and *mm*, the edematous regions surrounding the solid tumor region are predicted as *no*, again in agreement with the non-infiltrative nature of these tumors. In several cases/slices, a “dual” tumor type was predicted: the already-mentioned *lgg* for the solid tumor surrounding areas and a second type of tumor (again, see the example in Figure 5, case et2997). The nosological image of this glioblastoma marks the upper anatomically abnormal area as *mm* instead of the expected *lgg* or even *gb*, both for the 4-class task and the 3-class task. As can be noted, it is not possible that the patient has a *gb* and a *mm* at the same time, and the “yellow” color is due to the misclassification of these voxels, with the classifier assigning the *mm* class to an infiltrating pattern not properly recognized by the *gb* or *lgg* patterns, or a region with artifactual MV data. 

**Figure 6:**
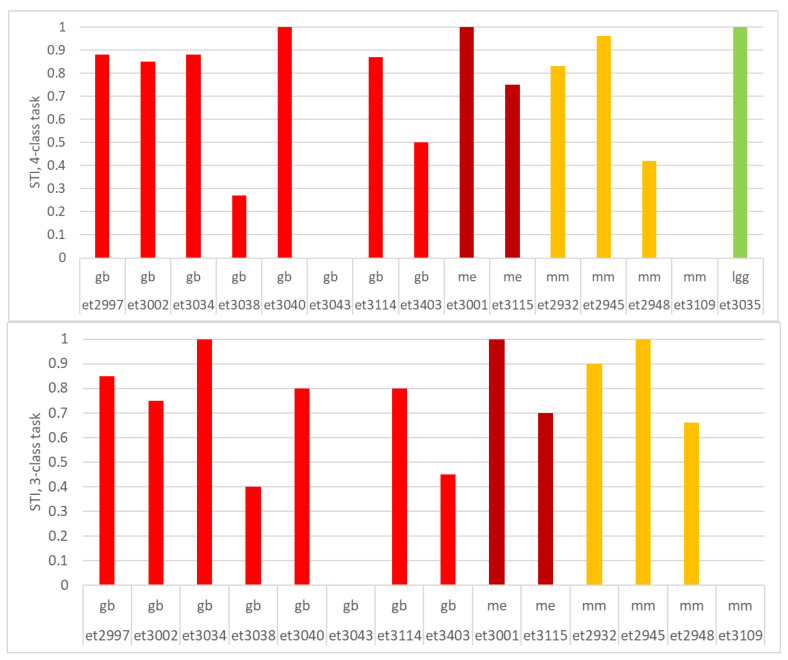
STI per case: top, 4-class task; bottom, 3-class task.

With respect to the aspects evaluated:

The STI index showed whether the class of the solid tumor region corresponded to the diagnosis of the patient. This was so in 66% (10 out of 15) of cases for the 4-class task and in 71.4% (10 out of 14) of cases for the 3-class task, taking a threshold of STI > 0.50 as an indication of success (Figure 6). In the 4-class task, there are three failed *gb*, et3038 with STI < 0.3, et3043 (multi-slice) with STI = 0, and et3403 (multi-slice) with STI = 0.5. From the four *mm*, two failed: et2948 with STI = 0.42 and et3109 (multi-slice), completely failing (STI = 0). The cases passing the STI threshold had a mean value of 0.89 overall. In the 3-class task, the failing cases were the same as for the 4-class task, and the mean STI of the successful cases was 0.84. 

**Figure 7:**
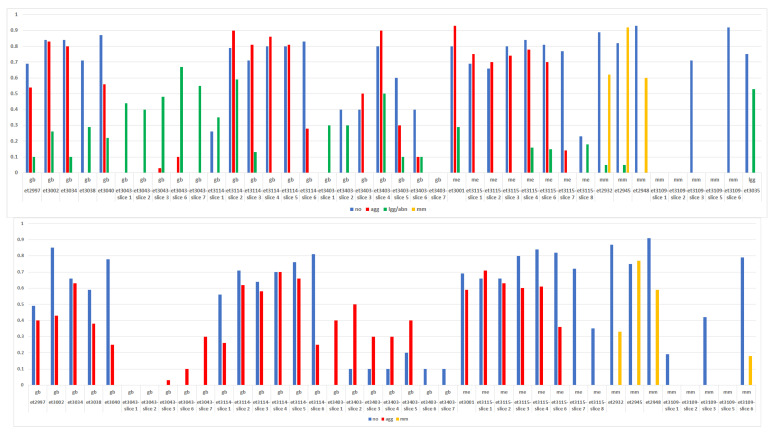
Dice score case-by-case and slice-by-slice for the multi-slice acquisitions. Top, 4-class task; bottom, 3-class task. Blue, normal brain; red, solid tumor region in *gb* or *me*; yellow, solid tumor region in *mm*; green, abnormal region (oedema, other).

The Dice score showed whether the localization of the solid tumor region agreed with the MRI segmentation performed by the radiologist. Figure 7 shows the Dice scores for all included slices and cases, for the 4-class task and the 3-class task. As expected, the results were similar to those of the STI, but some aspects must be highlighted. Beginning with the 4-class task, the multi-slice *gb* et3043 and the single-slice et3038 failed (no predicted *agg* region matched the anatomical solid tumor region). The multi-slice et3403 *gb* also failed in most slices except the central one (number 4). The remaining *gb* had Dice values for *agg* that were above 0.50. The single-slice me (et3001) had an excellent Dice value for *agg,* while in the multi-slice me (et3115) the prediction of the solid tumor region failed in the last two slices (7 and 8). In three out of four mm, the segmentation of the solid tumor region had a Dice value > 0.50 and again the prediction of this region for the multi-slice mm et3109 failed. For the *lgg*, the Dice value of the solid tumor region was > 0.50. With respect to the 3-class task, the Dice values were lower for the *gb* and me that had good results in the 4-class task, which were higher for the multi-slice cases et3043 and et3403. As expected, in general terms, the external slices of the multi-slice cases had lower Dice scores for the solid tumor region. 

The Dice scores also showed whether or not the surrounding abnormal areas agreed with the radiologist’s segmentation. This can also be examined in Figure 7 by looking at the *no* (blue) and the abnormal (green). Regarding the normal brain, it can be seen in the 4-class task that, in all cases, the Dice score for *no* (blue) was above 0.70, except for the two previously discussed multi-slice *gb* (et3043 and et3403), and that in the 3-class counterpart, there is a general decrease in Dice values for the normal region. With respect to the surrounding anatomically abnormal region that was identified by the radiologist, the first relevant finding was that the 4-class task captured it better than the 3-class task. This is shown graphically in Supplementary Materials B and C, case-by-case and slice-by-slice. Looking at the 4-class task in Figure 7, it can be observed that *gb* have variable Dice scores (green) for the peritumoral area depending on case and slice. It can be noted that the Dice scores for “green” regions are higher at the external slices (1…8) and the highest “red” Dice score is for the central slices, as expected (cases et3043 and et3403): *gb* normally have a necrotic core, surrounded by more infiltrative, *lgg*-like regions. Here, the Dice score is calculated between the anatomically abnormal region segmented by the radiologist and the area predicted as *lgg*-class. Therefore, it is to be expected that there is a certain degree of infiltration that is captured by the classifier in the form of *lgg* prediction. Interestingly, the case et3043 that failed in the solid tumor region delimitation had Dice scores ranging between 0.40 and 0.67. From the *me*, et3001 had a Dice score of 0.30 and et3115 had a Dice score < 0.20 in three out of eight slices. Of the three *mm* that were successful with STI, the Dice score for the surrounding area was even lower, as expected due to the non-infiltrative nature of these tumors, not reaching 0.10 in two out of three cases. 

**Figure 8:**
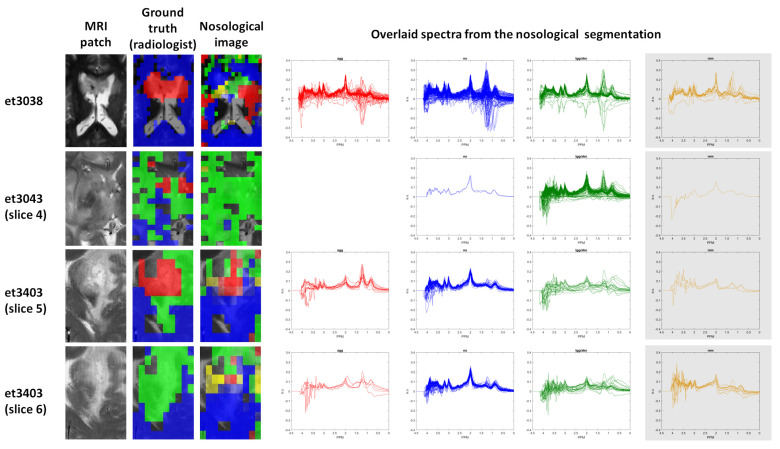
Four slices from three different cases in which the classifier predictions failed. All belong to the *gb* class. Rows: slices. Columns: MRI patch or MRI that corresponds to the same location as the MV MRS slice. Ground truth is the segmentation completed by the radiologist: blue, normal brain; red, solid tumor region in *gb* or *me*; yellow, solid tumor region in *mm*; green, abnormal region (oedema, other). In the nosological image on the third column, the color codes are the same, except that the green represents *lgg*. The fourth, fifth, sixth, and seventh columns show the overlaid individual spectra from the regions segmented in the nosological image.

With respect to the failed MV test set cases, some illustrative examples are shown in Figure 8. Case et3038 is a *gb* located inside the ventricles. This is a poor area for obtaining good homogeneity in the magnetic field, and indeed it can be observed in the last four columns of Figure 8 that a great proportion of flipped-down spectra (clearly artifactual) are distributed among the four classes. In addition, as the lipid features (0.886 ppm and 1.289/1.270 ppm on Figure 4) do not have high relevance in the chosen model (4-class task, Figure 4, left), some necrotic lipid areas also appear classified as normal (it is also noteworthy that most blue spectra from this case have a choline/creatine ratio close to 1 and higher NAA than creatine, which is characteristic of the normal brain; therefore, these possible necrotic lipids might even be due to voxel bleeding due to the already-mentioned poor homogeneity). As for case et3043, it is noteworthy that the anatomical segmentation in slices 4, 5, and 6 is clearly different, with the main area of necrosis on slice 5, which is to be expected if the tumor is studied in the three directions of space. Therefore, even this case is a *gb*, and one should not expect that all the anatomical regions of the tumor are colored in red, like *agg*. Spectroscopically, in slices 4, 5, and 6, the blue area would be correct, with no lipids, NAA 2–3 times higher than choline and creatine, and a choline/creatine ratio of 1. The green area would also be reasonable in metabolic terms, if we assume the proliferating area surrounding the necrotic core, either with a similar pattern to the normal plus mobile lipids or again due to the voxel bleeding effect from the necrotic areas. Regarding the yellow area, it is indeed the most contradictory, as it *gb* cannot be a *gb* and an *mm* at the same time. However, from the spectroscopic point of view, it is noteworthy that the spectral pattern of the yellow nosological areas is visually very much like the green nosological areas, only that there is a slight difference in the macromolecule region (higher in the yellow ones), precisely covering the region where the most important feature is located (2.382 ppm, see the Shapley values in the 4-class task, Figure 4, left). In short, the mm label would simply be due to the coded voxels displaying an abnormal pattern for which the *mm* classifier is the closest one, with the available classifiers.

There are other very important reasons for the misclassification of some areas in the different slices that can be seen in Supplementary Materials B and C. One of the main ones is that there are spectra with a low SNR value (see, for example, eT3043 slices 1, 2, 6, and 7 for the 3-class task in Supplementary Materials and also the flat lines on the baseline, as well as the green spectra in Figure 8), as well as the already-mentioned flipped-down spectra, and other artifacts such as bad water suppression, shifted data (misaligned), and/or ghosting, as can be seen in the noisy region between 3.5–4 ppm in the overlaid spectra in the bottom row of Figure 8.

## 4. Discussion

In this proof-of-concept study, we have shown that it is possible to successfully apply a classifier developed on multicenter SV MRS data acquired preoperatively at 1.5T from brain tumor patients to another set of similar patients that were studied with an improved MV MRS protocol based on the semi-Laser sequence, and in some cases also with multi-slice acquisitions and also at a double strength in the magnetic field (3T). Indeed, the system is not perfect as the classification failed in specific MV cases, but we also demonstrated that the failure was mostly due to the presence of artifactual data in the MV set. 

To our knowledge, this is the first study ever to attempt a SV–MV train–test machine learning experiment, although there is previous literature on the compatibility between 1.5T and 3T using machine learning techniques, only focusing on SV [34], or using MV datasets and treating them like SV data [55]. In particular, the study by Kounelakis et al. [55] is the most similar to ours, as the authors used data from 21 glioma patients from UMCN acquired with MV at 1.5T and 43 glioma patients from another hospital (LUH) with MV at 3T, using voxels in the solid tumor region. They also mention a “core 1.5T dataset” comprising 303 patients, which is very similar to the INTERPRET set, although the details are not specified. The authors made classifiers with SVM, obtaining better results for the 1.5T set than for the 3T set. They discussed the limitations of their results based on the different acquisition conditions and the low number of voxels on the LUH dataset, mainly due to the different shimming. However, there were other reasons that would have a greater influence in the classification performance, such as the data from UMCN being from a STEAM sequence at short TE (20 ms), while the LUH data were long TE (144 ms). As an example, in gliomas, the lactate doublet is inverted (flipped-down) at a TE of 144 ms, whereas at a TE of 20 ms it is pointing up, e.g., the TE is one parameter that should be compatible between different sets. Another limitation of that study was that the authors attempted a classification between different grades of glial tumors (WHO Grade II, II, and IV), and this has been known to be problematic due to the heterogeneity of the Grade III on the one hand, and on the other, due to the known differences in the histological classification of gliomas depending on the pathologist and the region sampled, before the current WHO classifications [56,57] based on genetic features. We have considered that the subsequent changes that happened in the WHO (e.g., the 2021 classification [56]) do not affect the purpose of our study, and we use these labels to distinguish among broad tumor super-classes, as will be discussed later. In our study, we did not use the two intermediate Grade III gliomas for this reason. Instead, we used a classification problem that has been successfully tested in many studies [21,24,38], i.e., the *agg* vs. *lgg* vs. *mm* distinction, to which we added the *no* class, which can also be distinguished without problems [29,35]. Another work with more traceable data is one that focused on SV data [34], taking the same INTERPRET dataset that we used for training and an eTDB SV dataset for testing. Fuster-García et al. in [34] trained classifiers on the bilateral task of *agg* vs. *lgg*, using SV data from the INTERPRET project, and tested them with a set of 37 SV spectra acquired with two different scanners from two centers: 21 of them were acquired with a GE Signa 3T with a TR of 2000–5000 ms, a TE of 30 ms, an SW of 1000 Hz, and 2048 data points, and the other 16 spectra were acquired with a Philips 3T scanner using a TR of 1800–2000 ms, a TE of 32ms, a spectral width of 2000 Hz, and 1024 data points. In [34], the processing procedure was also the same as that of our study. The feature selection methods were SFFS and peak integration. The classifiers were LDA, k-nearest neighbors (KNN), and artificial neural networks (ANN). Average test accuracies in the ranges of 86–87% were found, which are comparable to our reported AUCs.

Other studies using SV MRS at 3T report results in the same range as ours. For example, Zarinabad et al. [58] report balanced accuracy rates (BAR) of 0.81 with LDA, 0.86 with SVM, and 0.89 with RF on a cohort of 41 child brain tumors, SV MRS at 3T, after 100 runs of over-sampling. It is noteworthy that BAR= 1-BER and the cohort size is ca. 1/6 of ours. Also, in children brain tumors, Zhao et al. [59] report a maximum performance achieved with LOO cross-validation of BAR = 0.85 at 1.5T with 116 SV from three different child tumor cases using SVM and a BAR = 0.75 at 3T with 73 child tumor cases, using LDA after over-sampling the minority class.

Another study by Tsolakis et al. [60] using the combination of data acquired at 3T with MV PRESS and DSC-MR from 35 *gb* and 14 *me* patients, achieved 0.98 accuracy for the peritumoral area using the NAA/ Cr ratio and rCBV followed by Naïve Bayes.

Pedrosa de Barros et al. [61] conducted a study on 41 training (7624 spectra) and 17 testing (3276 spectra) cases, acquired at long TE 1.5T with MV MRS, to distinguish between edema, healthy brain without Glx, active tumor, and necrosis. In gliomas, cNMF was used to determine the signal sources in the healthy brain and glioma and were compared with the ground truth as determined via the automated segmentation of the anatomy with the BraTumIA software. In that study, Dice scores or similar are not reported and a pairwise Pearson correlation table is provided between the MRI classes and the MRS classes, making a comparison with our study difficult. In that study, the authors also provide the segmentation images as demonstration. For spectral quality control, they used their in-house methodology based on machine learning (RF) [62], similarly to us.

Our classification results are not only comparable to previous studies on similar datasets, but in most instances achieve better results on the independent test set, which is the most independent of all the ones reviewed above. Our independent test set is from several years afterwards (2006–2009 vs. 1994–2002), from a different center (UMCN vs. the INTERPRET partners), a partially different manufacturer (Siemens vs. Philips or GE), a different sequence (semi-Laser vs. PRESS or STEAM), and a different magnetic field (3T vs. 1.5T).

In our study, we used a variety of techniques for dimensionality reduction: filter, wrapper, and embedded methods. In the filter or univariate methods, the relationship between features and class labels is considered, but not redundancy. In the wrapper or multivariate methods, features are selected iteratively by maximizing the prediction accuracy of the classifier. The embedded methods are very similar to the wrapper techniques since they are likewise used to streamline the target capacity or execution of a learning calculation or model. The distinction of wrapper strategies is that a natural model structure metric is utilized amid learning. In our study, we found that the wrapper method used (SFFS) worked better. We attribute this to the fact that cross-validation was used to evaluate feature selection. All methods tested performed similarly in the training phase but were prone to overfitting. In addition, SFFS selected a low number of features that corresponded to the maximum intensities of the most prominent peaks in the spectra, whereas other methods did not. The results are nevertheless not surprising, as it was also consistently found in previous studies with the same dataset that SFFS performed well [24,63], and the features we found are the same that were found previously [39]. Regarding the classification algorithms, LDA out-performed RF and SVM, in agreement with previous studies on the same dataset [35]. Although RF classifiers are one of the best and most robust techniques, it has been shown that when the dataset’s noise increases, RF performs worst [64]. Although RF performs as well as LDA in the training phase, LDA handled the noisy data better than RF. 

Importantly, the results from our study can be replicated, as the SFFS/LDA classifier we used is included in an MRS classifier software SpectraClassifier [28], which has demonstrated good performance in other studies [39,63,65].

The main limitation of our results is the low spectral quality of the MV dataset. Despite several steps (SNR thresholding, manual phase correction, renormalization to minimize incomplete water suppression at the processing stage, and the additional cNMF approach for identifying the artifactual patterns), many artifactual spectra still remained. We could have applied more complete quality control measures, such as the FWHM measure to detect poor water suppression or other methodologies, but this was not the purpose of our study, and in addition, our MV spectra did not have an accompanying unsuppressed water file. When it had, it had a different dimension than the metabolite file, and therefore it was not possible to perform a FWHM measurement to discard spectra with poor homogeneity [66,67]. In fact, automatic quality control is nowadays a hot topic in the MRS arena. Currently, no single automated method exists for filtering out poor-quality spectra, neither for SV nor MV, and expert-based routine checking of each individual spectrum of every MV MRS acquired in a clinical setting is simply not feasible. The existing literature puts forward several promising approaches for quality-control-based machine learning, as discussed in the introduction [13,20,68], but sometimes the data used to train are synthetic, and in others the systems are trained to detect only one artifact and none of these approaches has been tested in a real-world scenario, taking into account hardware and software from multiple scanner vendors and acquisition parameters, etc. One important problem with our dataset was the phase correction, which we had to correct individually with suboptimal results. In this respect, future studies could incorporate new approaches that are being developed, for example, as in [19], where the authors used an unsupervised deep-learning approach for phase and frequency correction. 

A possible limitation of our approach with MV PRESS data is what is known as chemical shift displacement artifact (CSDA), in which there is a displacement in the localization of the VOI caused by the differences in the chemical shift, with an artifactual decrease in the intensity of the NAA peak in the normal brain, which could fool the classifier. Sequences such as semi-Laser [12,15,53] offer an effective solution for this artifact and in this case our nosological images did not encounter the CSDA. However, it remains to be shown whether, in a more standard clinical scenario without the availability of the semi-Laser, comparable results to the present ones would have been achieved.

Another possible limitation concerns the individual voxel neuroradiological labelling, as all morphological MR sequences were not always available for all patients. Nevertheless, the detailed evaluation by an expert neuroradiologist ensures the ground truth. Further studies should rely on the whole set of images for the exact delimitation of the anatomical abnormality regions and also on the evaluation of adjacent voxels, as in [6]. 

Another aspect that could be seen as a limitation is the ground truth based on the radiologist, in particular for the Dice scores we obtain that do not necessarily match the radiologist’s segmentations in the peritumoral area. It is not feasible or ethical in human patients to systematically validate the infiltration with a biopsy. In some instances it could be performed with some limitations; for example, in a recent work [69], the authors report that for some patients (not all), they validated possible infiltration by taking three targets from the tumor core, the peri-tumor region, and the margins via stereotactic biopsy. Even so, it is impossible to sample the entire brain. It has already been known that the metabolic abnormality does not exactly match the anatomical abnormality [70], although the surrounding abnormality is a well-accepted characteristic of gliomas [71,72]. However, the radiologist’s segmentations continue to be used as the ground truth in many MRS studies due to such ethical limitations [73]. 

With respect to the implications of our study in the development of decision support systems for brain tumors, based on MV MRS, we foresee that they could be used to obtain a prediction of the degree of malignancy (Grade IV or not) and the broad tumor type (glial, meningeal). They could also be used to predict whether the tumor is infiltrative (if the solid tumor region is surrounded by a tumoral low-grade glial-like area), which is important in distinguishing between glioblastoma and solitary metastasis. 

In the case that additional curated SV datasets from other pathologies become available, it could be possible to extend such an approach to different or more specific clinical differential diagnoses. One limitation of our approach, for example, is the availability of only one *lgg* case. 

Our initial purpose was to train with SV and test with MV, but given the data available, we ended up training with 1.5T and testing with 3T. Therefore, another question for future studies would be how far, in terms of magnetic field strength (e.g., 7T), such an approach can go: in our case, data were made compatible by down-sampling at a spectral resolution of ca. 0.014 ppm/point.

Another issue related to resolution that affects the range of potential applications is the voxel size of MV, with most centers acquiring in the 0.5–1cm^3^ range, which is insufficient for some applications such as radiotherapy planning. However, a scenario in which MRS datasets increase their spatial resolution with specialized sequences [74] could also be the ground for extending the range of applications, provided that first it is demonstrated that the data are compatible in machine learning terms. 

The fact that, despite the above-mentioned limitations, and even with many artifactual spectra, we obtained reasonable and informative segmentations in most of the investigated cases, failing cases with poor spectral quality, points towards the elephant in the room for any future clinical applications of MRS. Clinicians should trust their datasets, and for this, scanner manufacturers or post-processing programs should provide reliable artifact detection. If the data is of good quality, class and segmentation predictions based on machine learning will be reliable as we showed; otherwise, the results will not be reliable.

## 5. Conclusions

We have shown that it is possible to train machine learning models on the SV data of brain tumors, at low field (1.5T), and apply these classifiers to metabolically segment MV data obtained from double the magnetic field (3T) from independent centers, scanner manufacturers, formats, and acquisition conditions. This was possible due to a processing pipeline that unified the number of points and the frequency range and normalized unit length, as well as using similar echo times (short TE). 

We have also shown that the predictions fail in the test set mainly because of the presence of artifactual spectra that were not properly filtered out at the processing stage. 

Our work opens the door for future studies in which the compatibility between different magnetic fields and acquisition conditions can be tested, as well the influence of the different types of artifacts that can affect the newest MRS acquisitions. Finally, we demonstrate the value of working, curating, and sharing old SV datasets for training machine learning models applicable to advanced MRS datasets, which is not the norm nowadays.

## Figures and Tables

**Table 1:** Summary of the acquisition parameters used in the SV short TE dataset.

Parameter	SV STEAM	SV PRESS
**Magnetic field**	1.5T	1.5T
**TE**	20–32 ms	30–32 ms
**TR**	1600–2000 ms	1600–2000 ms
**Volume**	4–8 cm^3^	4–8 cm^3^
**N averages metabolites**	256	192–128
**N averages water**	8–32	8–16
**N points**	512 (Philips)	512 (Philips)
	1024 (Siemens)	1024 (Siemens)
	2048 (GE)	2048 (GE)
**Bandwidth**	1000 Hz (Philips)	1000 Hz (Philips)
	1000 Hz (Siemens)	1000 Hz (Siemens)
	2500 Hz (GE)	2500 Hz (GE)
**Dummy scans**	4	4

**Table 2:** Relevant parameters of the MV data after applying the case inclusion criteria.

Parameter	Value
**Magnetic field**	3T
**Sequence**	Semi-Laser
**Model and scanner brand**	TrioTim Siemens
**Software version**	Syngo MR B13 4VB13A
**TE**	30 ms
**TR**	1000 ms
**FOV**	16 × 16 × 8/32 × 16 × 8/ 32 × 32 × 1
**VOI**	8 × 18 × 8/10 × 6 × 8/10 × 8 × 8/10 × 10 × 8/18 × 14 × 1/18 × 15 × 1/18 × 20 × 1/18 × 21 × 1/20 × 14 × 1/20 × 16 × 1/20 × 20 × 1/20 × 24 × 1/22 × 20 × 1/25 × 16 × 1
**Hanning filter**	100%
**Slice thickness**	10 mm
**N averages metabolites**	1–3
**N averages water**	1
**N points**	1024 or 4096
**Bandwidth**	2404 or 4000 Hz

**Table 3:** BER results for the 4-class task, under the different combinations of feature selection methods and classifiers, for the SV training and the MV test sets. The results are depicted as a heatmap, where the most intense green corresponds to the best results (lowest BER). “Drop” in this context means the Pearson’s correlation-based threshold applied (i.e., “50 drop” means the threshold was set at 0.5 correlation, and we dropped those variables with a correlation higher than 0.5). “Full” means we used the whole set of features.

***Training Set***	**Number of Features**														
**Feature Selection Method**	**3**	**4**	**5**	**6**	**7**	**8**	**9**	**10**	**50 Drop**	**60 Drop**	**70 Drop**	**80 Drop**	**90 Drop**	**Full**	**Classifier**
**SFFS**	0.12	0.10	0.10	0.10	0.10	0.08	0.08	0.06							**LDA**
**SFFS**	0.38	0.28	0.30	0.65	0.20	0.37	0.39	0.36							**SVM**
**SFFS**	0.09	0.08	0.04	0.04	0.03	0.04	0.02	0.03							**RF**
**Chi**	0.24	0.17	0.13	0.54	0.53	0.49	0.06	0.51							**LDA**
**Chi**	0.21	0.17	0.11	0.45	0.47	0.46	0.07	0.49							**SVM**
**Chi**	0.14	0.12	0.10	0.36	0.39	0.38	0.07	0.11							**RF**
**K-best**	0.34	0.20	0.17	0.19	0.14	0.11	0.14	0.11							**LDA**
**K-best**	0.40	0.12	0.12	0.09	0.11	0.09	0.10	0.11							**SVM**
**K-best**	0.05	0.05	0.06	0.04	0.11	0.09	0.10	0.11							**RF**
**Lasso**	0.17	0.11	0.08	0.12	0.09	0.08	0.08	0.16							**LDA**
**Lasso**	0.15	0.11	0.08	0.10	0.09	0.08	0.08	0.10							**SVM**
**Lasso**	0.13	0.10	0.08	0.10	0.10	0.10	0.10	0.11							**RF**
**Boruta**									0.09	0.09	0.09	0.14	0.16	0.07	**LDA**
**Boruta**									0.06	0.07	0.06	0.07	0.05	0.06	**SVM**
**Boruta**									0.03	0.01	0.02	0.03	0.03	0.01	**RF**
***Test Set***	**Number of Features**														
**Feature Selection Method**	**3**	**4**	**5**	**6**	**7**	**8**	**9**	**10**	**50 Drop**	**60 Drop**	**70 Drop**	**80 Drop**	**90 Drop**	**Full**	**Classifier**
**SFFS**	0.38	0.34	0.35	0.34	0.33	0.27	0.33	0.41							**LDA**
**SFFS**	0.68	0.60	0.60	0.39	0.64	0.64	0.64	0.40							**SVM**
**SFFS**	0.53	0.42	0.46	0.59	0.56	0.57	0.44	0.47							**RF**
**Chi**	0.71	0.68	0.31	0.66	0.68	0.68	0.49	0.66							**LDA**
**Chi**	0.69	0.66	0.56	0.65	0.68	0.69	0.55	0.69							**SVM**
**Chi**	0.68	0.67	0.55	0.63	0.64	0.70	0.56	0.70							**RF**
**K-best**	0.43	0.43	0.43	0.34	0.23	0.21	0.36	0.23							**LDA**
**K-best**	0.64	0.48	0.50	0.59	0.57	0.58	0.54	0.59							**SVM**
**K-best**	0.52	0.47	0.55	0.59	0.57	0.58	0.54	0.59							**RF**
**Lasso**	0.53	0.48	0.55	0.51	0.50	0.47	0.47	0.50							**LDA**
**Lasso**	0.54	0.53	0.51	0.53	0.57	0.49	0.49	0.59							**SVM**
**Lasso**	0.53	0.48	0.52	0.55	0.57	0.58	0.58	0.59							**RF**
**Boruta**									0.42	0.33	0.24	0.20	0.27	0.30	**LDA**
**Boruta**									0.45	0.47	0.46	0.46	0.48	0.44	**SVM**
**Boruta**									0.47	0.48	0.48	0.47	0.48	0.45	**RF**

**Table 4:** BER results for the 3-class task, under the different combinations of feature selection methods and classifiers, for the SV training and the MV test sets. The results are depicted as a heatmap, where the most intense green corresponds to the best results (lowest BER). “Drop” in this context means the Pearson’s correlation-based threshold applied (i.e., “50 drop” means the threshold was set at 0.5 correlation, and we dropped those variables with a correlation higher than 0.5). “Full” means we used the whole set of features.

***Training Set***	**Number of Features**											
**Feature Selection Method**	**3**	**4**	**5**	**6**	**7**	**8**	**9**	**10**	**80 Drop**	**90 Drop**	**Full**	**Classifier**
**SFFS**	0.07	0.07	0.07	0.07	0.07	0.07	0.05	0.05				**LDA**
**SFFS**	0.23	0.20	0.19	0.06	0.06	0.06	0.05	0.06				**SVM**
**SFFS**	0.40	0.33	0.35	0.10	0.08	0.15	0.10	0.11				**RF**
**Chi**	0.12	0.12	0.39	0.05	0.06	0.07	0.07	0.09				**LDA**
**Chi**	0.10	0.11	0.36	0.05	0.05	0.06	0.05	0.07				**SVM**
**Chi**	0.09	0.08	0.49	0.06	0.07	0.05	0.05	0.06				**RF**
**K-best**	0.13	0.17	0.07	0.04	0.08	0.13	0.12	0.12				**LDA**
**K-best**	0.15	0.12	0.10	0.07	0.06	0.11	0.10	0.10				**SVM**
**K-best**	0.23	0.16	0.11	0.11	0.10	0.14	0.12	0.13				**RF**
**Lasso**	0.08	0.08	0.05	0.05	0.05	0.05	0.03	0.04				**LDA**
**Lasso**	0.09	0.04	0.06	0.08	0.07	0.06	0.06	0.03				**SVM**
**Lasso**	0.08	0.06	0.07	0.06	0.07	0.06	0.07	0.06				**RF**
**Boruta**									0.23	0.10	0.06	**LDA**
**Boruta**									0.09	0.12	0.10	**SVM**
**Boruta**									0.09	0.10	0.04	**RF**
***Test Set***	**Number of Features**											
**Feature Selection Method**	**3**	**4**	**5**	**6**	**7**	**8**	**9**	**10**	**80 Drop**	**90 Drop**	**Full**	**Classifier**
**SFFS**	0.25	0.25	0.26	0.26	0.27	0.26	0.19	0.25				**LDA**
**SFFS**	0.38	0.38	0.38	0.27	0.29	0.28	0.32	0.33				**SVM**
**SFFS**	0.44	0.44	0.43	0.33	0.32	0.35	0.33	0.33				**RF**
**Chi**	0.42	0.41	0.61	0.32	0.33	0.33	0.32	0.45				**LDA**
**Chi**	0.39	0.39	0.58	0.35	0.35	0.36	0.35	0.44				**SVM**
**Chi**	0.39	0.41	0.67	0.33	0.34	0.36	0.34	0.40				**RF**
**K-best**	0.50	0.40	0.40	0.35	0.30	0.38	0.32	0.27				**LDA**
**K-best**	0.48	0.58	0.41	0.43	0.46	0.48	0.47	0.46				**SVM**
**K-best**	0.44	0.53	0.38	0.41	0.44	0.50	0.48	0.48				**RF**
**Lasso**	0.39	0.27	0.28	0.28	0.26	0.28	0.33	0.29				**LDA**
**Lasso**	0.36	0.35	0.28	0.35	0.33	0.32	0.32	0.44				**SVM**
**Lasso**	0.31	0.24	0.32	0.33	0.34	0.35	0.35	0.34				**RF**
**Boruta**									0.39	0.32	0.27	**LDA**
**Boruta**									0.49	0.43	0.44	**SVM**
**Boruta**									0.46	0.44	0.51	**RF**

**Table 5:** Three- and four-class AUC using SFFS and LDA.

		AUC			
	Set	*mm*	*agg*	*lgg*	*no*
**4-class task**	**SV train**	0.99	0.97	0.98	0.99
	**MV test**	0.95	0.89	0.82	0.82
**3-class task**	**SV train**	0.99	0.98	-	1.00
	**MV test**	0.90	0.83	-	0.82

## Data Availability

Available on request.

## Supplementary Materials

The following supporting information can be downloaded at: https://www.mdpi.com/article/10.3390/cancers15143709/s1. Supplementary Materials A: *Supplementary Table S1* *for: Using Single Voxel Magnetic Resonance Spectroscopy Data Acquired at 1.5T to Classify Multivoxel data at 3T: a Proof of Concept for Brain Tumour Diagnosis*; Supplementary Materials B: *SUPPLEMENTARY MATERIALS “B” Using Single Voxel Magnetic Resonance Spectroscopy Data Acquired at 1.5T To Classify Multivoxel Data at 3T: A Proof-of-Concept Study*; Supplementary Materials C: *SUPPLEMENTARY MATERIALS “C”: Using Single Voxel Magnetic Resonance Spectroscopy Data Acquired at 1.5T to Classify Multivoxel Data at 3T: A Proof-of-Concept Study.*

## Abbreviations

LDA: linear discriminant analysis; *lgg*: low-grade glioma; *me*: metastasis; *mm*: meningioma; MRS: magnetic resonance spectroscopy; *no*: normal; RF: random forest; SFFS: sequential forward feature selection; SV: single-voxel; TE: echo time; WM: white matter; *gb*: glioblastoma multiforme; STI: Solid Tumor Index; SVM: Support Vector Machine.
