# Supplementary material for: Using Single-Voxel Magnetic Resonance Spectroscopy Data Acquired at 1.5T to Classify Multivoxel Data at 3T: A Proof-of-Concept Study
Source: Cancers (Basel). 2023 Jul 21;15(14):3709. doi: 10.3390/cancers15143709 (PMC10377805; doi:10.3390/cancers15143709)
Supplement: Supplementary file 1 [file cancers-15-03709-s001.zip › Supplementary Materials A-20230720.pdf]

Using Single-Voxel Magnetic Resonance Spectroscopy Data Acquired at 1.5T to Classify Multivoxel Data at 3T: A Proof-of-Concept Study

Gülnur Ungan [1,2], Albert Pons-Escoda [3], Daniel Ulinic [2], Carles Arús [1,2], Alfredo Vellido [1,4] and Margarida Julià-Sapé [1,2,*]

[1] Centro de Investigación Biomédica en Red (CIBER), 28029 Madrid, Spain; gulnur.ungan@autonoma.cat (G.U.); carles.arus@uab.cat (C.A.); avellido@lsi.upc.edu (A.V.)
[2] Departament de Bioquímica i Biologia Molecular and Institut de Biotecnologia i Biomedicina (IBB), Universitat Autònoma de Barcelona (UAB), 08193 Barcelona, Spain; daniel.ulinic@uab.cat
[3] Group de Neuro-Oncologia, Institut d'Investigació Biomèdica de Bellvitge (IDIBELL), Hospital Universitari de Bellvitge, 08908 Barcelona, Spain; albert.pons@bellvitgehospital.cat
[4] IDEAI-UPC Research Center, UPC BarcelonaTech, 08034 Barcelona, Spain
* Correspondence: margarita.julia@uab.cat

Supplementary Table S1. Details of the MV eTDB included cases, ordered by case number. The last row of the table shows total numbers for the column of interest.

							Number of voxels after QC				
**CASE ID**	**PARAFFIN SECTION WHO CLASSIFICATION (eTDB pathologists' consensus)**	Characteristics of the lesions	**FOV**	**VOI**	**Number of slices**	**Total number of voxels inside of the VOI**	**Solid tumour region**	**Abnormal region**	**Normal brain tissue**	**Discarded due to QC**	**Number of slices after QC and SNR threshold for visualisation**
et2491	ANAPLASTIC OLIGODENDROGLIOMA 9451/3		32x32	22x20	1	440	77	42	224	96	1
et2932	ATYPICAL MENINGIOMA 9539/1		32x32	20x16	1	320	12	47	253	8	1
et2945	FIBROUS MENINGIOMA 9532/0		32x32	18x20	1	360	79	36	213	32	1
et2948	MENINGIOMA 9530/0		32x32	18x15	1	270	24	10	223	10	1
et2997	GLIOBLASTOMA 9440/3		32x32	20x24	1	480	65	23	291	101	1
et3001	METASTASIS 8000/6		32x32	20x20	1	400	27	44	316	12	1
et3002	GLIOBLASTOMA 9440/3		32x32	25x16	1	400	33	65	275	27	1
et3034	GLIOBLASTOMA 9440/3		32x32	18x20	1	360	62	26	225	47	1
et3035	DIFFUSE ASTROCYTOMA 9400/3		32x32	18x14	1	252	3	25	177	47	1
et3038	GLIOBLASTOMA 9440/3		32x32	18x21	1	378	63	0	227	88	1
et3040	GLIOBLASTOMA 9440/3		32x32	20x14	1	280	12	31	04	33	1
et3043	GLIOBLASTOMA 9440/3		16X32	8x18	8	1152	114	209	248	90	5
et3109	MENINGIOMA 9530/0		16X16	10x8	8	640	10	0	439	180	5
et3114	GLIOBLASTOMA 9440/3		16X16	10x6	8	480	52	46	178	25	6
et3115	METASTASIS 8000/6		16X16	10x8	8	640	64	138	385	53	7

CASE ID	PARAFFIN SECTION WHO CLASSIFICATION (eTDB pathologists' consensus)	Characteristics of the lesions	FOV	VOI	Number of slices	Total number of voxels inside of the VOI	Number of voxels after QC: Solid tumour region	Abnormal region	Normal brain tissue	Discarded due to QC	Number of slices after QC and SNR threshold for visualisation
et3401	ANAPLASTIC ASTROCYTOMA 9401/3		16X16	10x10	8	800	17	55	438	62	6
et3403	GLIOBLASTOMA 9440/3		16X16	10x10	8	800	139	126	271	200	7
						8452	853	923	4387	1111	47

Supplementary Table S2. Characteristics of the lesions of the eTDB included cases, ordered by case number. The last row of the table shows total numbers for the column of interest.

CASE ID	PARAFFIN SECTION WHO CLASSIFICATION (eTDB pathologists' consensus)	Radiological characteristics of the lesions
**et2491**	ANAPLASTIC OLIGODENDROGLIOMA 9451/3	Likely intra-axial, ill-defined, extensive, infiltrative lesion in both frontal lobes, extending across the corpus callosum. High T2 heterogeneity. A cluster of cystic areas and foci of very low-T2 signal noted in the left frontal lobe. Relatively moderate mass effect.
**et2932**	ATYPICAL MENINGIOMA 9539/1	Likely extra-axial, predominantly expansive, left parietal lesion. T2 hypointense, with ill-defined contour in relation to underlying brain cortex. Relatively abundant parenchymal oedema and moderate to severe mass effect.
**et2945**	FIBROUS MENINGIOMA 9532/0	Extra-axial, well-defined, expansive, T2-isointense right parietal mass with partial CSF cleft. Very abundant parenchymal oedema and moderate to severe mass effect.
**et2948**	MENINGIOMA 9530/0	Likely extra-axial, predominantly expansive, right frontal lesion. T2 isointense, with partial CSF cleft but ill-defined contour in relation to underlying brain cortex in its posterior aspect. Mild oedema and mass effect.
**et2997**	GLIOBLASTOMA 9440/3	Intra-axial infiltrative-expansive lesion in the right frontal lobe extending towards the ependymal surface in the frontal horn of the ipsilateral ventricle. Heterogeneous T2 signal, with a large area of necrosis and infiltration into the frontal cortex. Moderate oedema and moderate to severe mass effect.
**et3001**	METASTASIS 8000/6	Intra-axial, well-defined, expansive, lesion in the left frontal lobe. Ring-like appearance with central necrosis. Abundant perilesional oedema and moderate mass effect.
**et3002**	GLIOBLASTOMA 9440/3	Intra-axial, ill-defined, infiltrative-expansive lesion in the left frontal-parietal region. T2-isointense with mildly heterogeneous enhancement. Moderate oedema and mass effect.
**et3034**	GLIOBLASTOMA 9440/3	Intra-axial, infiltrative, midline, posterior transcallosal lesion extending to the right frontal-parietal region and left thalamus through the splenium. High T2 heterogeneity.
**et3035**	DIFFUSE ASTROCYTOMA 9400/3	Intra-axial left temporal lesion, T2-heterogenous lesion with foci of very low-T2 signal intensity and tiny cystic-necrotic areas. Mild mass effect.
**et3038**	GLIOBLASTOMA 9440/3	Intra-axial, infiltrative, midline, rostral transcallosal lesion. Foci of very low-T2 signal intensity and tiny cystic-necrotic areas. Heterogeneous enhancement. Mild mass effect.
**et3040**	GLIOBLASTOMA 9440/3	Intra-axial infiltrative-expansive lesion in the left temporo-parietal region. T2-heterogegenus with regions of necrosis. Moderate oedema and mass effect.
**et3043**	GLIOBLASTOMA 9440/3	Intra-axial extensive infiltrative lesion involving the right frontal, temporal, opercular, and insular regions with extension into the ipsilateral basal ganglia and corpus callosum and crossing to the contralateral paramedial frontal cortex and basal ganglia. Bilateral frontal and anterior caudate-capsular patchy enhancements. Moderate to severe mass effect.

**et3109**	MENINGIOMA 9530/0	Extra-axial, well-defined, expansive, T2-isointense left temporal mass with partial CSF cleft. Mild mass effect.
**et3114**	GLIOBLASTOMA 9440/3	Intra-axial infiltrative-expansive lesion in the left temporal lobe. T2-heterogeneous with regions of necrosis and extensive perilesional oedema. Moderate mass effect.
**et3115**	METASTASIS 8000/6	Intra-axial, well-defined, expansive, T2-heterogeneous lesion in the left frontal region. Abundant perilesional oedema and moderate mass effect.
**et3401**	ANAPLASTIC ASTROCYTOMA 9401/3	Intra-axial infiltrative-expansive lesion in the left thalamus. T2 heterogeneous with areas of necrosis. Relatively moderate mass effect.
**et3403**	GLIOBLASTOMA 9440/3	Intra-axial, extensive infiltrative lesion in the left temporal, frontal, opercular, and insular regions with extension into the ipsilateral basal ganglia. Mass-like component in the basal ganglia, T2 heterogeneous, with signs of necrosis. Moderate to severe mass effect.
