# Supplementary material for: Using Single-Voxel Magnetic Resonance Spectroscopy Data Acquired at 1.5T to Classify Multivoxel Data at 3T: A Proof-of-Concept Study
Source: Cancers (Basel). 2023 Jul 21;15(14):3709. doi: 10.3390/cancers15143709 (PMC10377805; doi:10.3390/cancers15143709)

Supplementary Materials "C":

Using Single Voxel Magnetic Resonance Spectroscopy Data Acquired at 1.5T to Classify Multivoxel Data at 3T: A Proof-of-concept Study

Gülnur Ungan [1,2], Albert Pons-Escoda [3], Daniel Ulinic [3], Carles Arús [1,2], Alfredo Vellido [1,4] and Margarida Julià-Sapé [1,2*]

1 Centro de Investigación Biomédica en Red (CIBER), 28029 Madrid, Spain; gulnur.ungan@autonoma.cat (G.U.); carles.arus@uab.cat (C.A.); avellido@lsi.upc.edu (A.V.)

2 Departament de Bioquímica i Biologia Molecular and Institut de Biotecnologia i Biomedicina (IBB), Universitat Autònoma de Barcelona (UAB), 08193 Barcelona, Spain; daniel.ulinic@uab.cat

3 Group de Neuro-Oncologia, Institut d'Investigació Biomèdica de Bellvitge (IDIBELL), Hospital Universitari de Bellvitge, 08908 Barcelona, Spain; albert.pons@bellvitgehospital.cat

4 IDEAI-UPC Research Center, UPC BarcelonaTech, 08034 Barcelona, Spain

*Correspondence: margarita.julia@uab.cat

4 Class

Legend of the next slides

Case ID (Grid Size)

Class. Balanced Error Rate (BER). Solid Tumor Index(STI)

MRI Patch	Nosological Image Groundtruth<br>Aggressive (agg)<br>Meningioma (mm)<br>Surrounding abnormal area<br>Unaffected normal brain area	Nosological Image Predicted<br>Aggressive (agg)<br>Meningioma (mm)<br>Surrounding abnormal area<br>Unaffected normal brain area	Area Under Curve For Each Class Nosological Image
Aggressive (agg) overlaid spectra (Red)	**Meningioma (mm) overlaid spectra (Yellow)**	Normal (no) overlaid spectra (Blue)	**Low grade glioma/abnormal (lgg/abn) overlaid spectra (Green)**

eT2997(20x24)
Glioblastoma. BER=0.47, STI=0.88

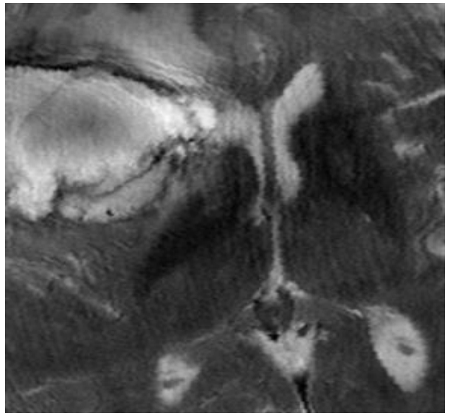

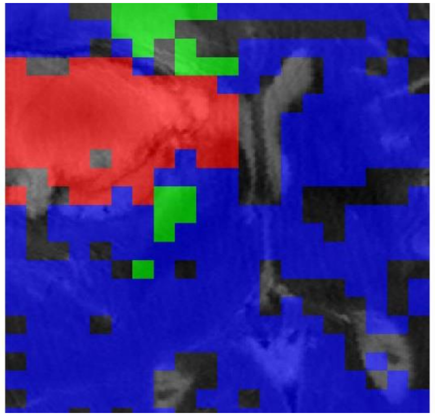

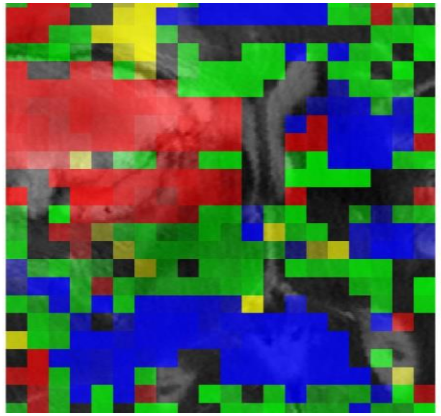

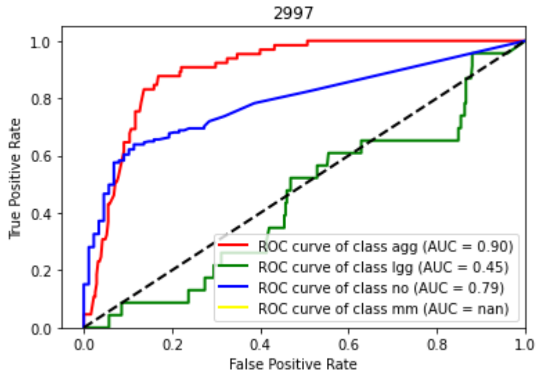

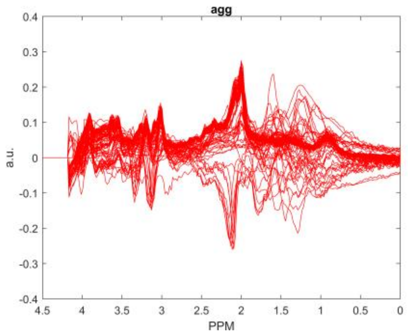

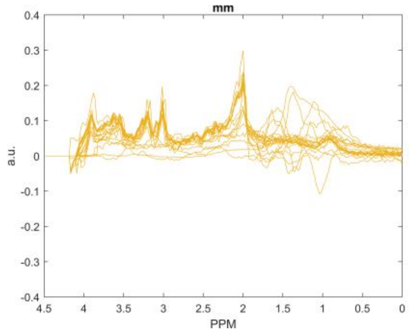

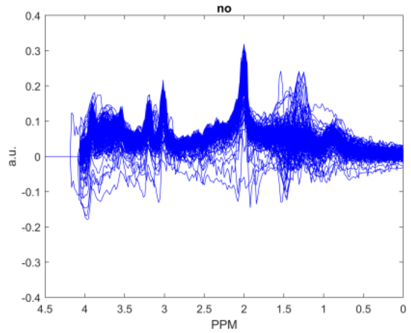

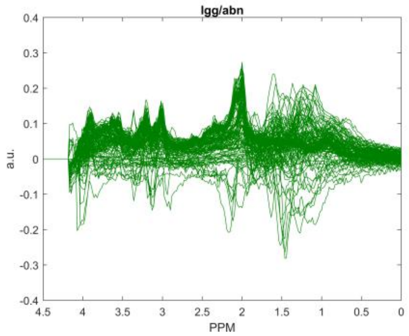

eT3002(25x16)
Gliobastoma. BER=0.39, STI=0.85

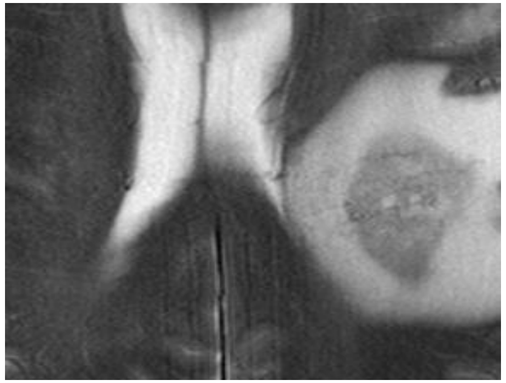

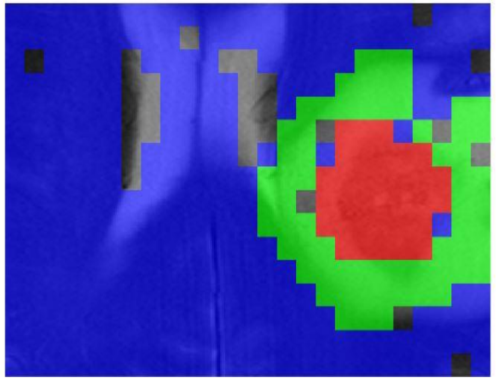

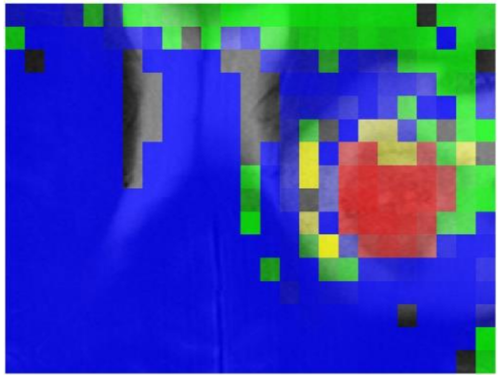

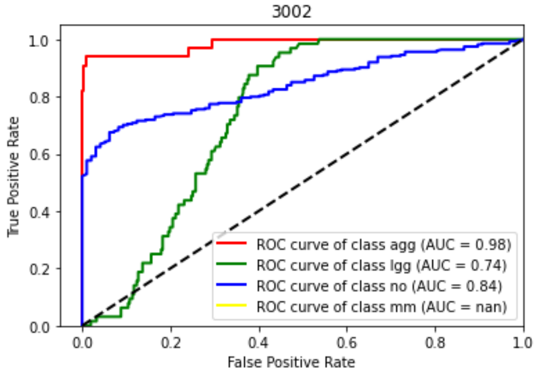

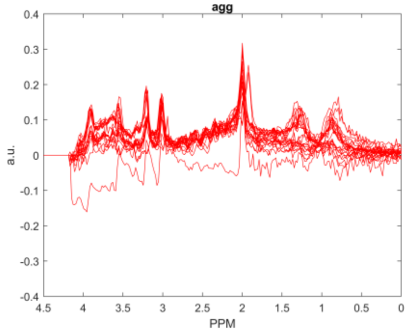

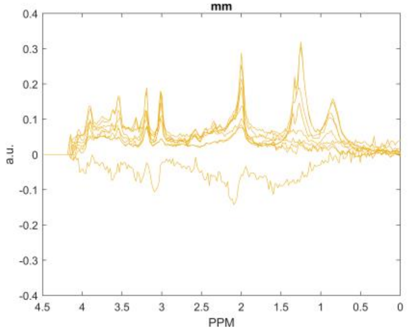

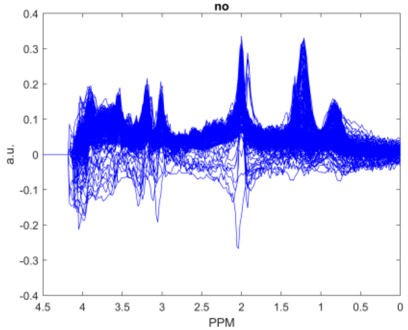

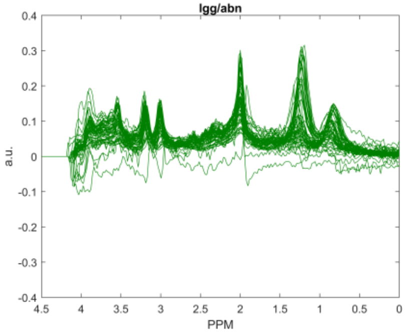

eT3034(18x20)
Glioblastoma. BER=0.38, STI=0.88

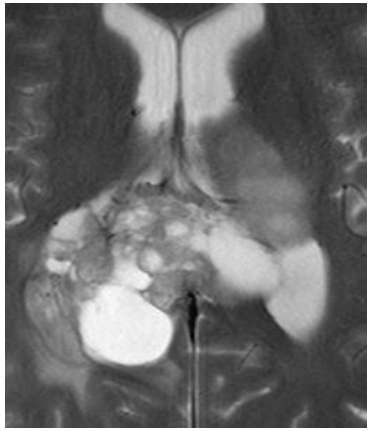

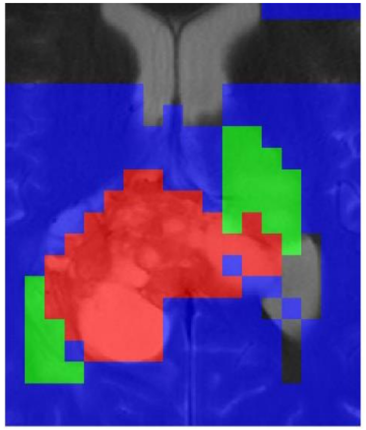

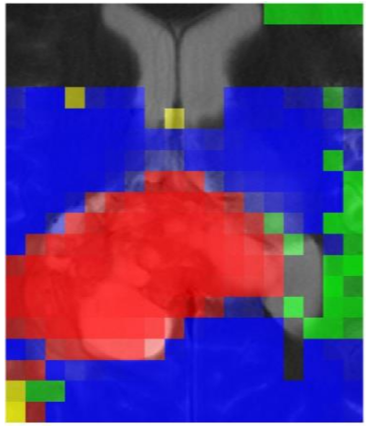

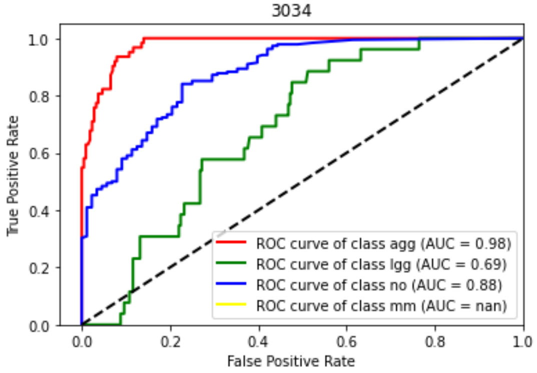

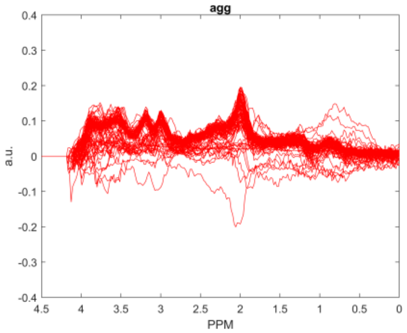

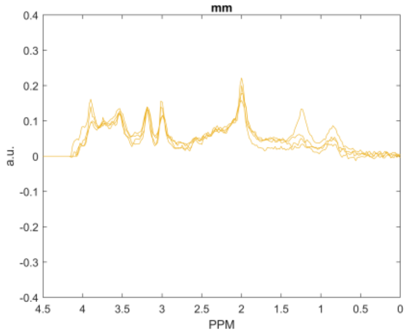

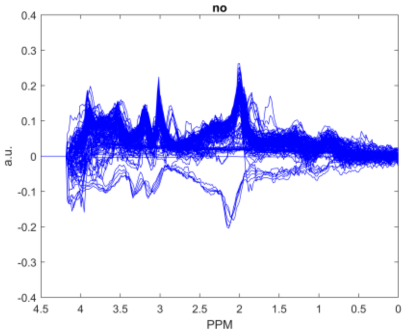

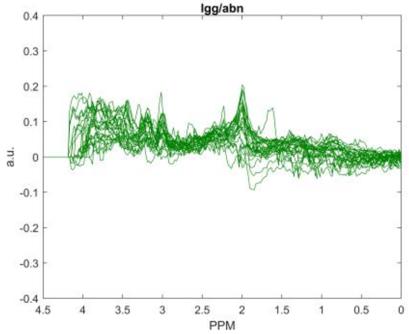

eT3038(18x21)
Glioblastoma. BER=0.56, STI=0.27

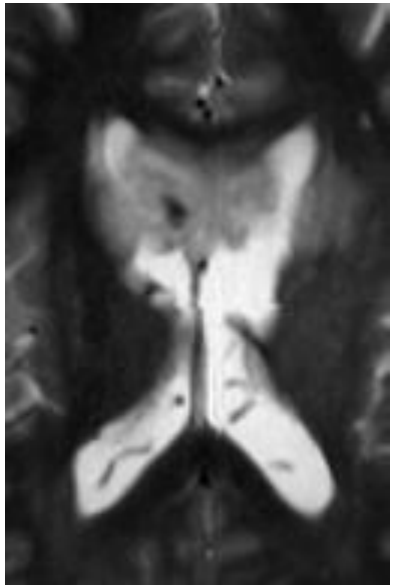

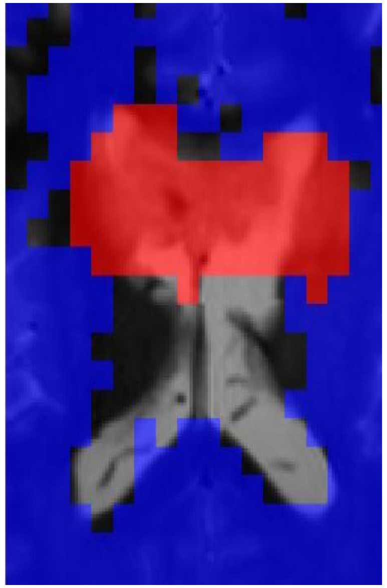

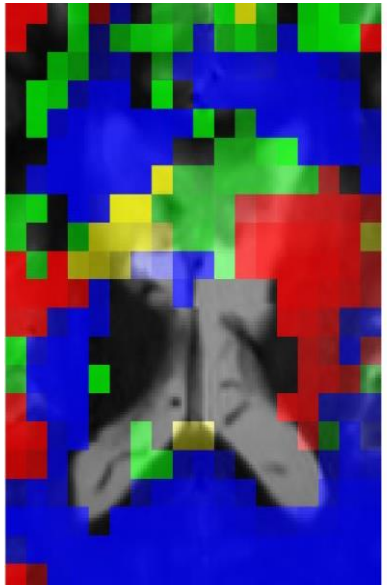

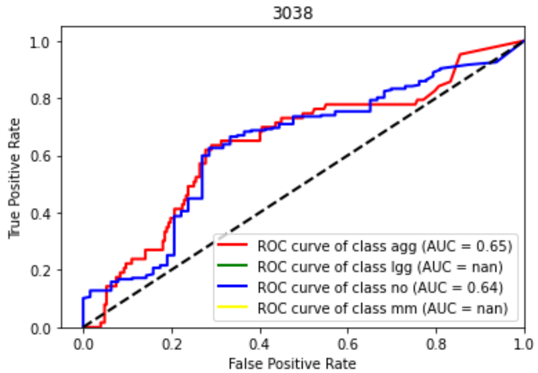

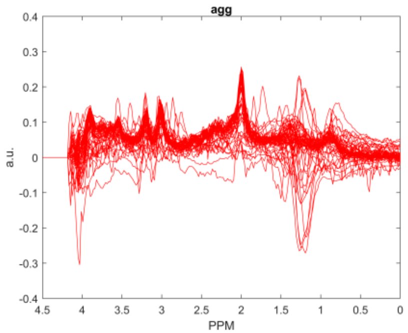

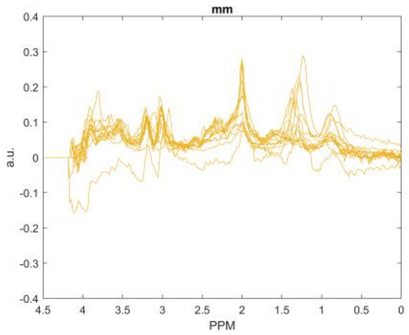

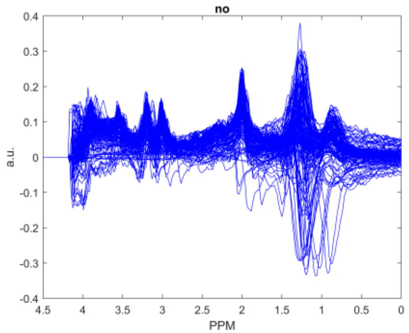

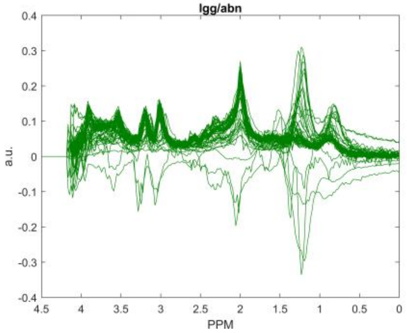

eT3040(20x14)
Glioblastoma. BER=0.33, STI=1.0

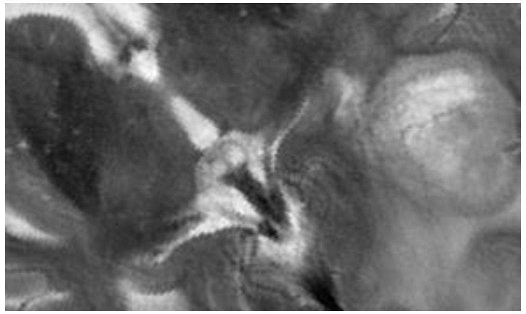

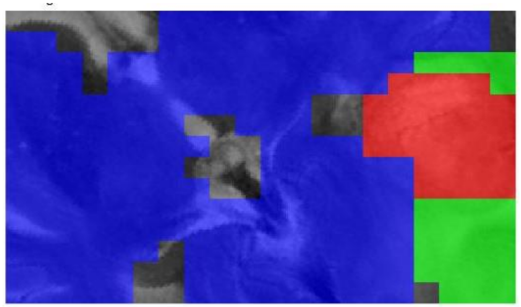

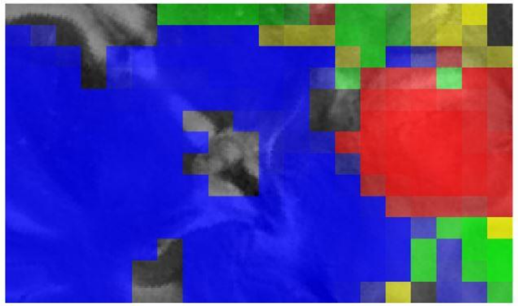

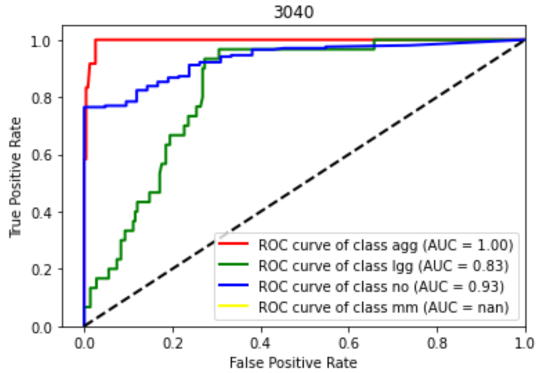

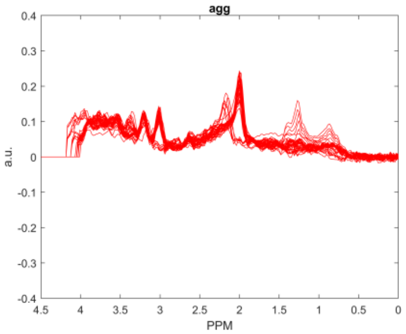

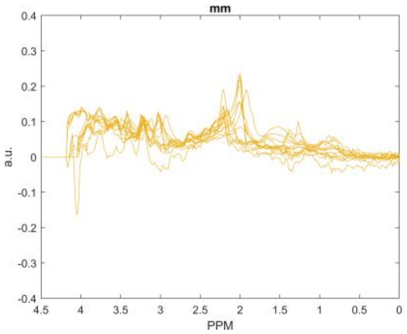

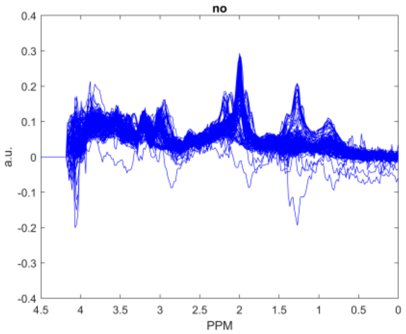

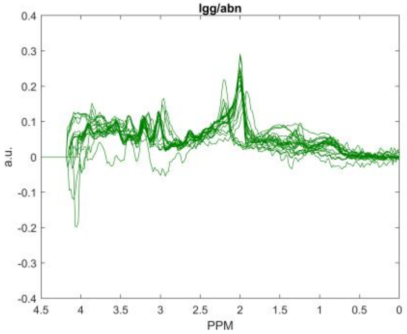

eT3043, S1(8x18)
Glioblastoma. BER=0.66, STI=0.5

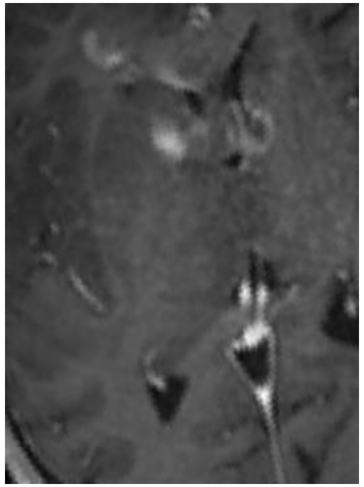

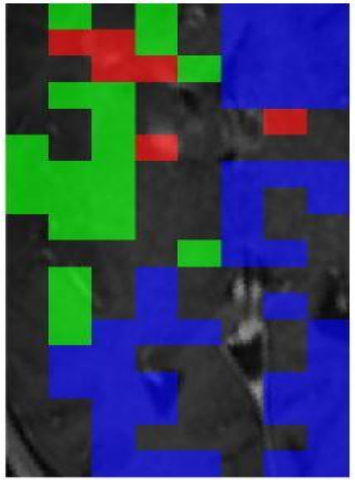

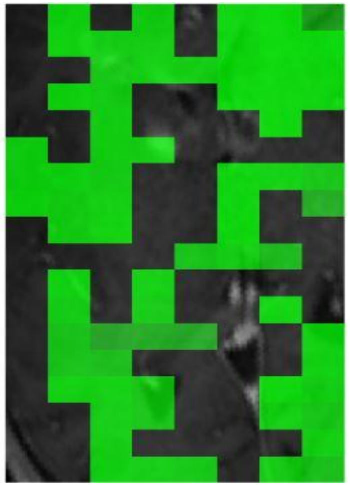

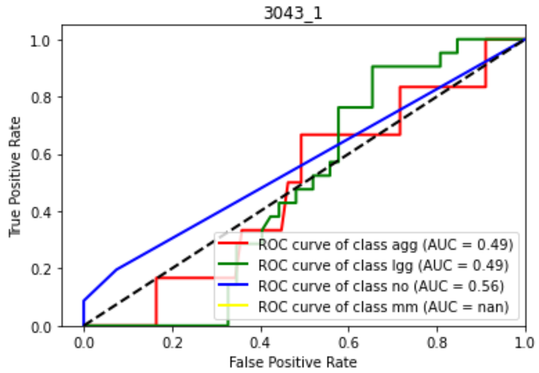

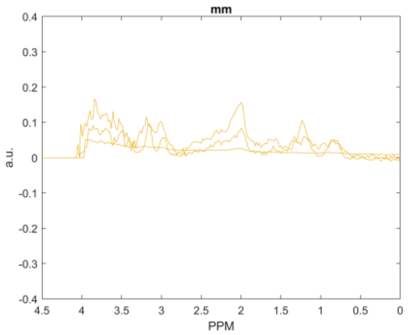

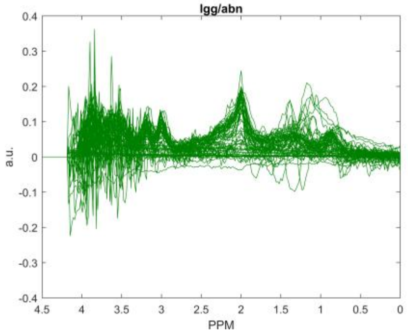

eT3043, S2(8x18)
Glioblastoma. BER=0.66, STI=0.5

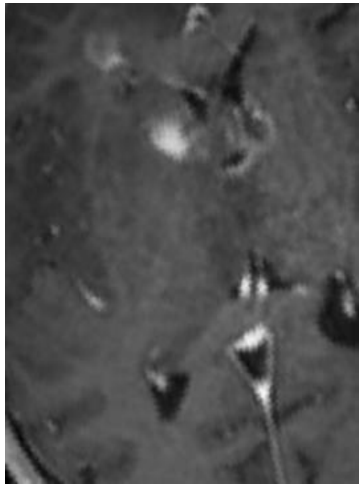

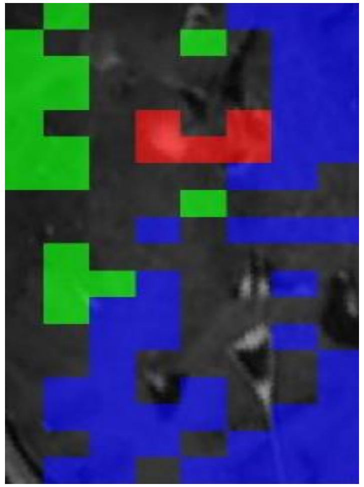

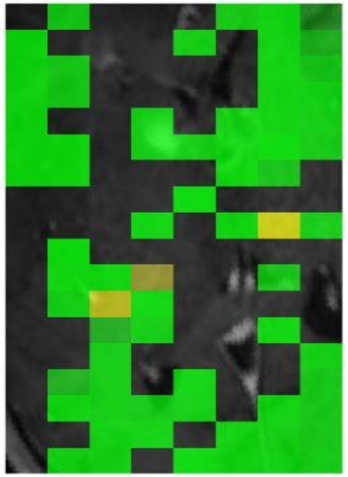

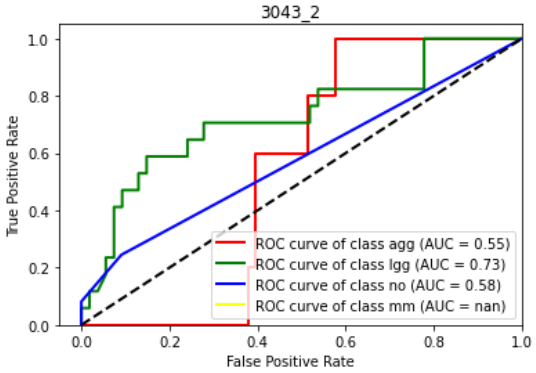

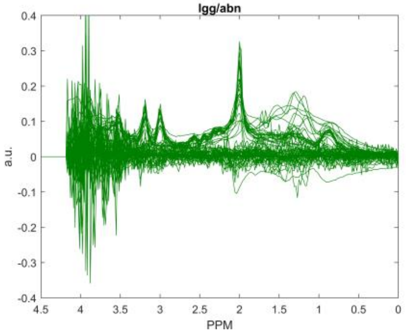

eT3043, S3(8x18)
Glioblastoma. BER=0.66, STI=0.5

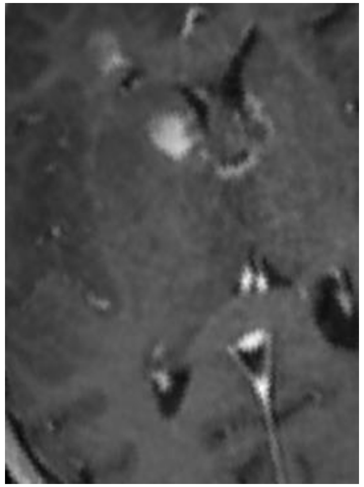

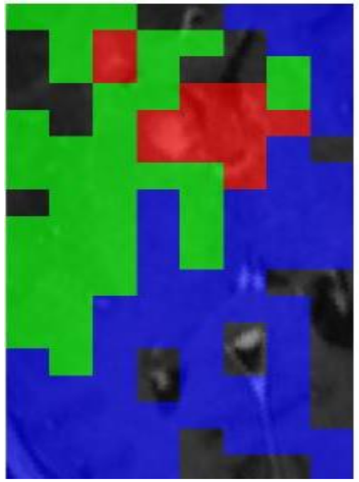

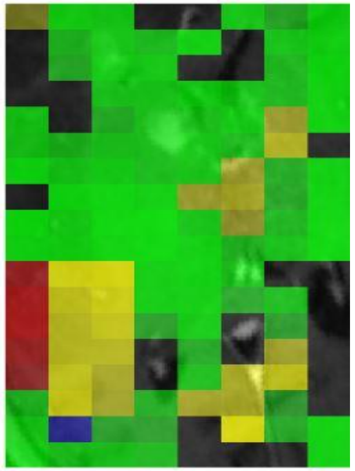

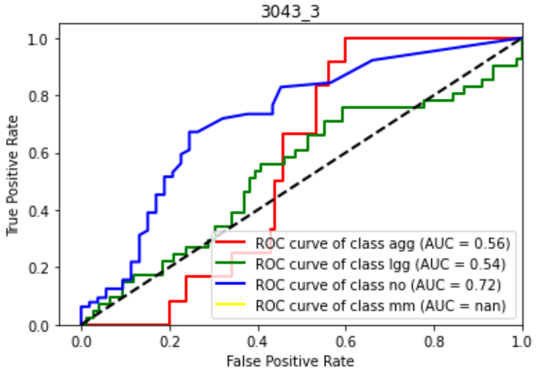

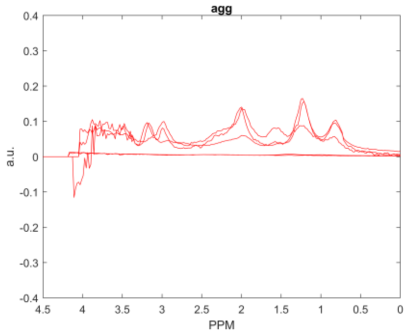

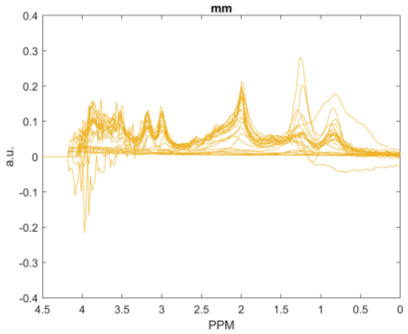

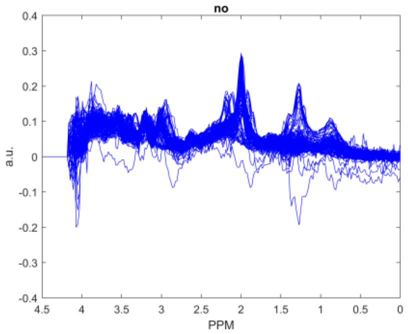

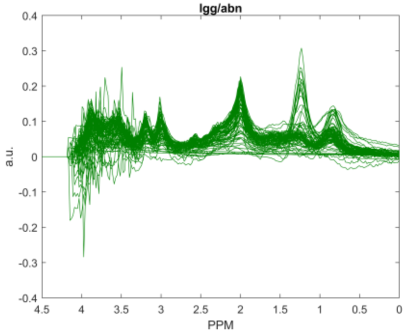

eT3043, S6(8x18)
Glioblastoma. BER=0.66, STI=0.5

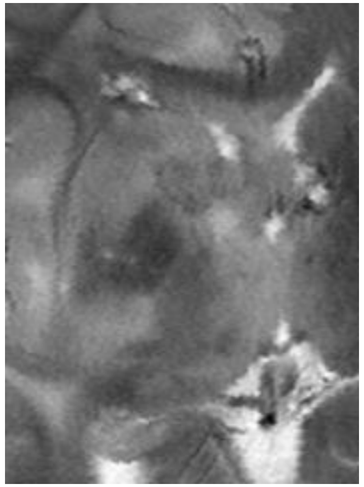

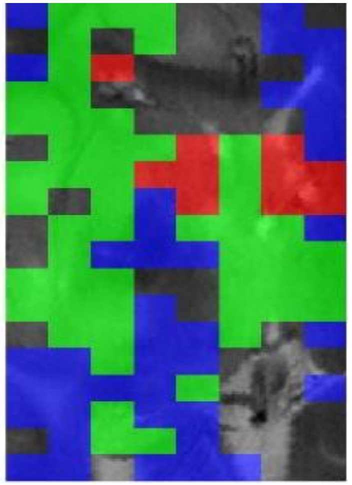

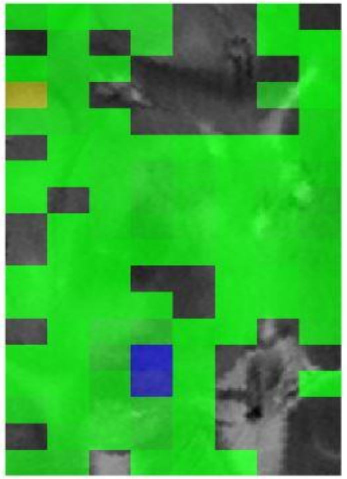

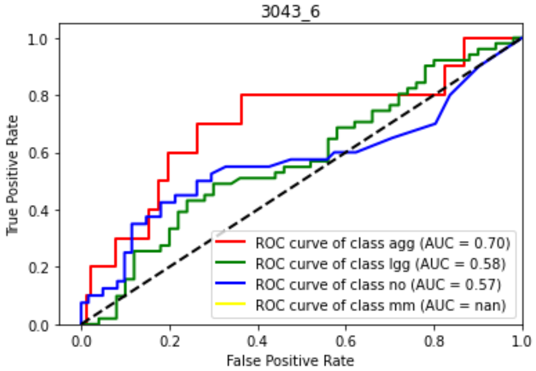

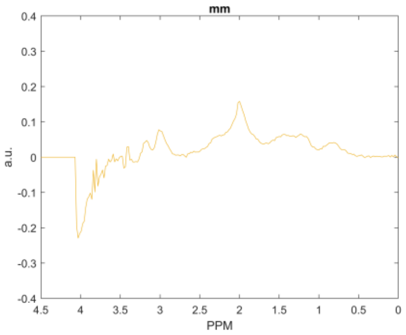

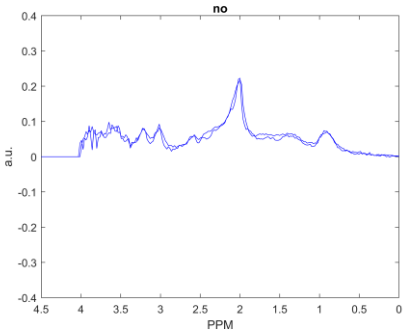

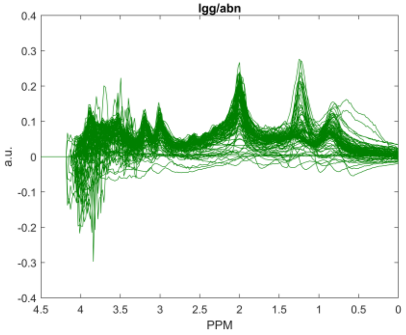

eT3043, S7(8x18)
Glioblastoma. BER=0.66, STI=0.5

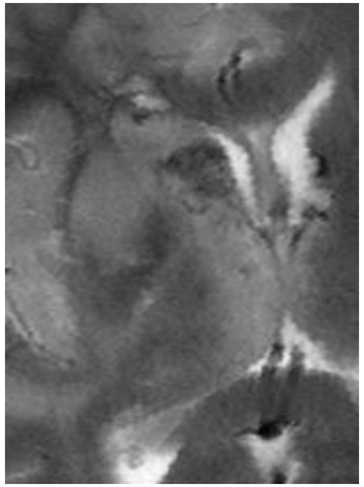

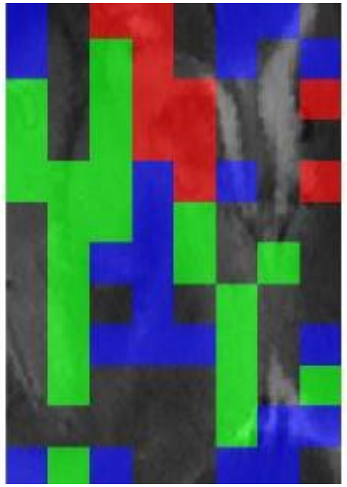

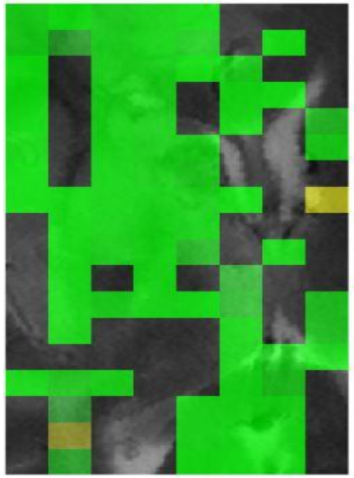

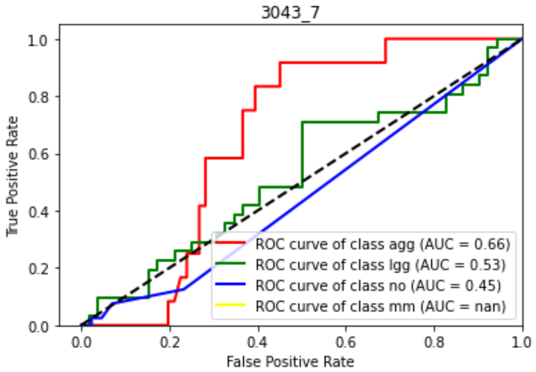

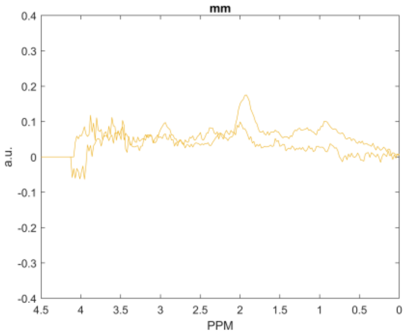

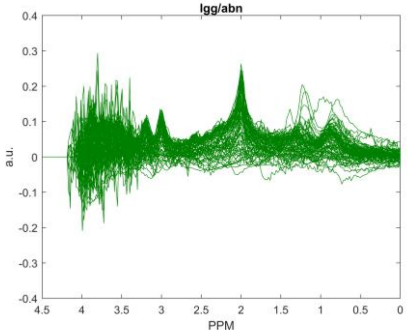

eT3114, S1(10x6)
Glioblastoma. BER=0.65, STI=0.87

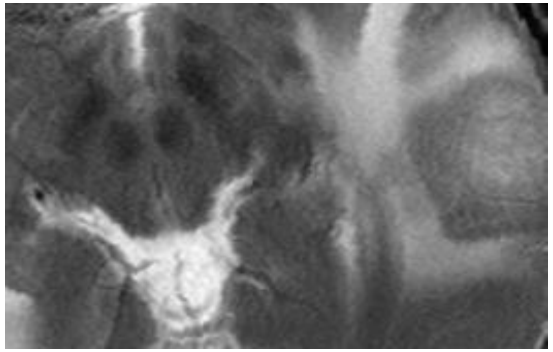

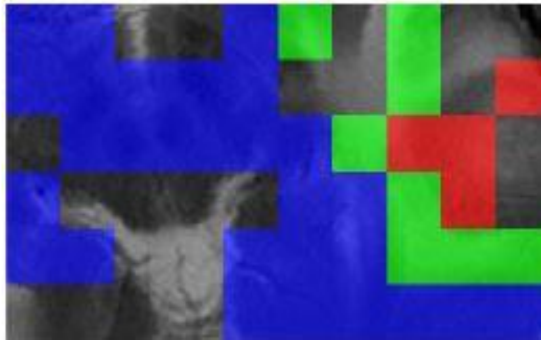

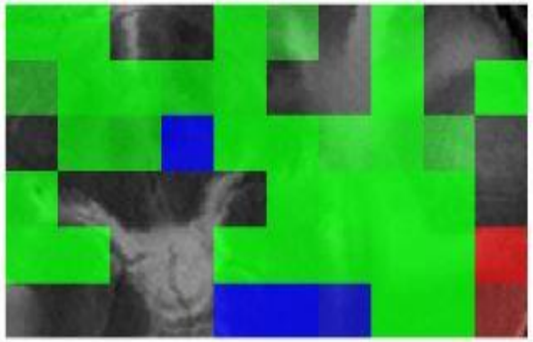

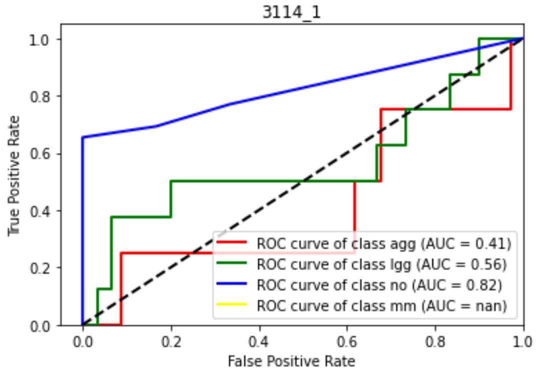

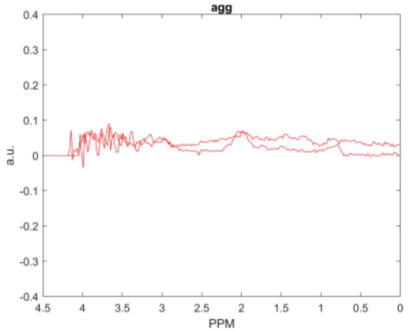

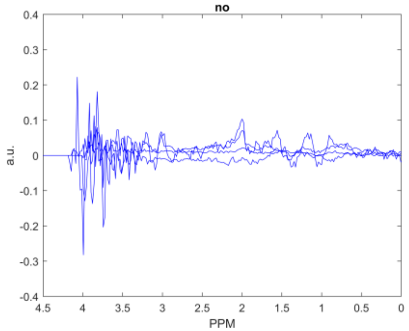

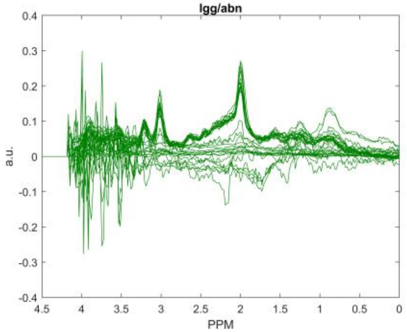

eT3114, S2(10x6)
Glioblastoma. BER=0.23, STI=0.87

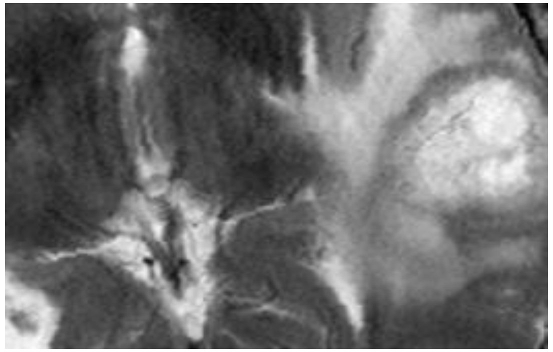

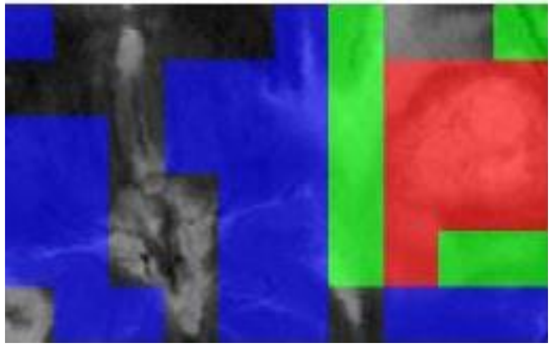

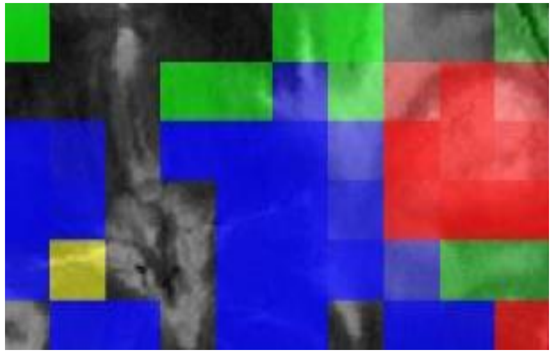

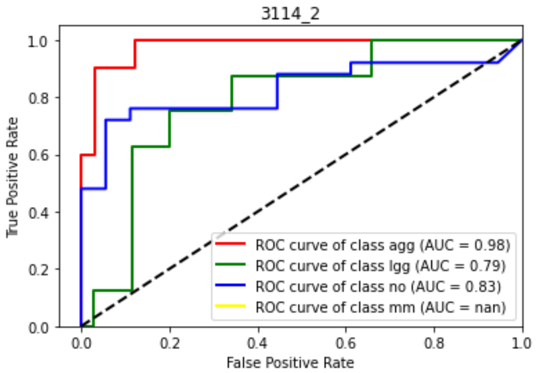

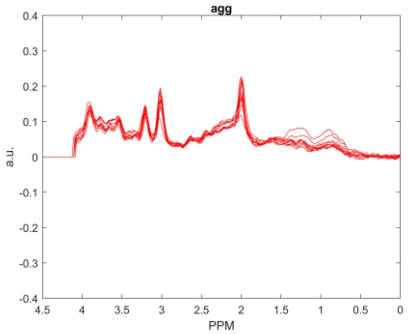

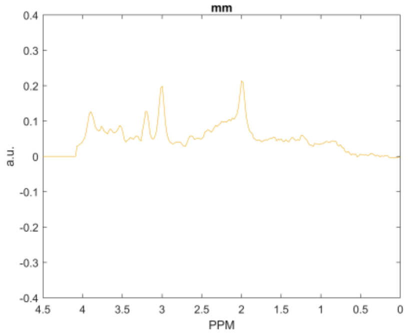

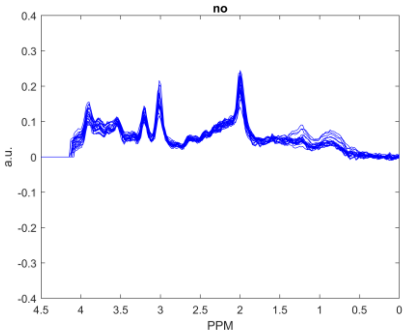

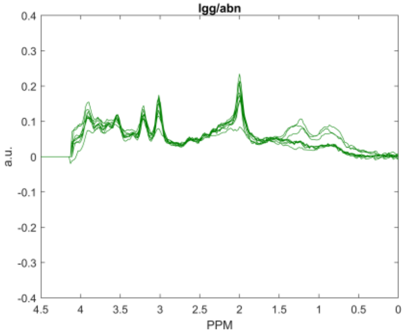

eT3114, S3(10x6)
Glioblastoma. BER=0.35, STI=0.87

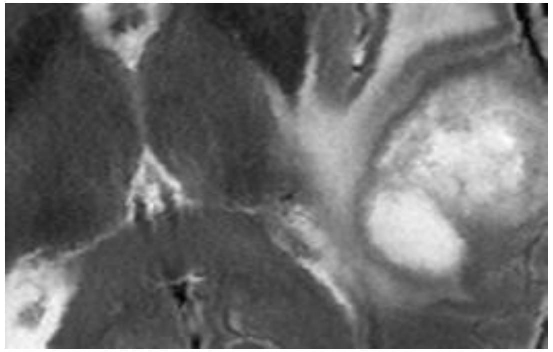

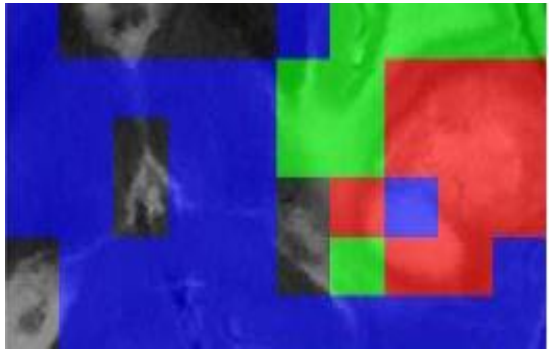

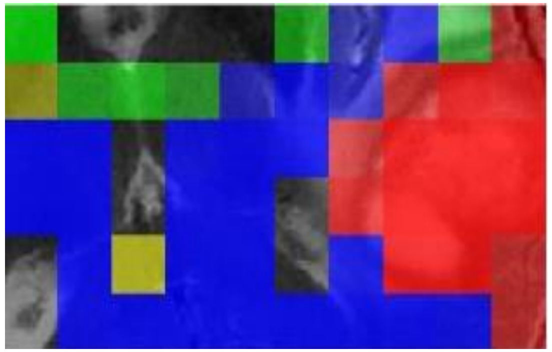

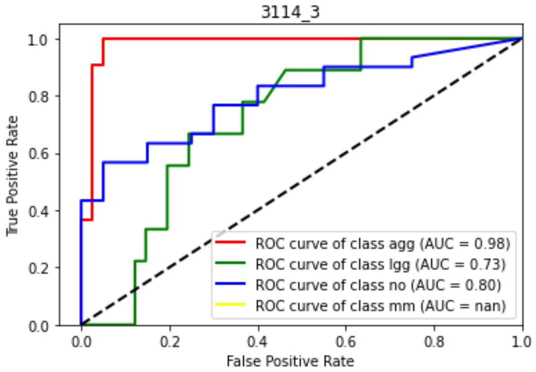

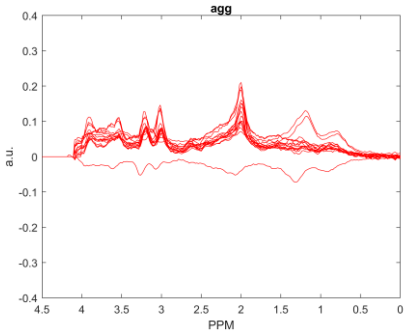

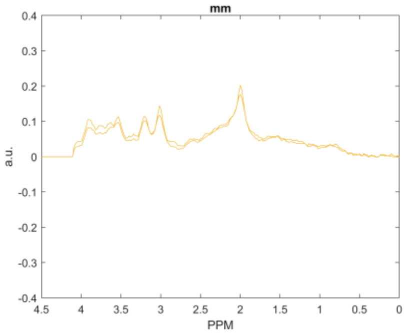

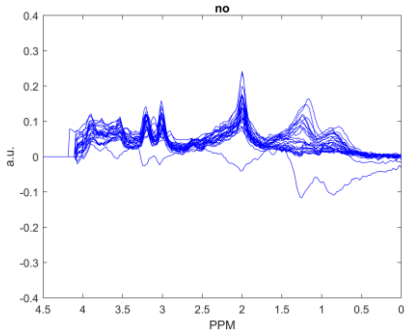

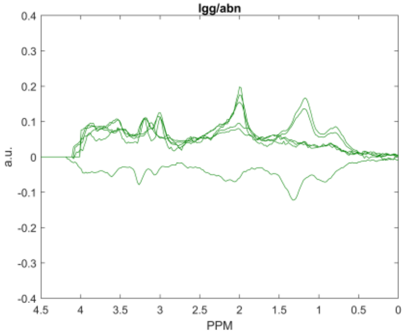

eT3114, S5(10x6)
Glioblastoma. BER=0.42, STI=0.87

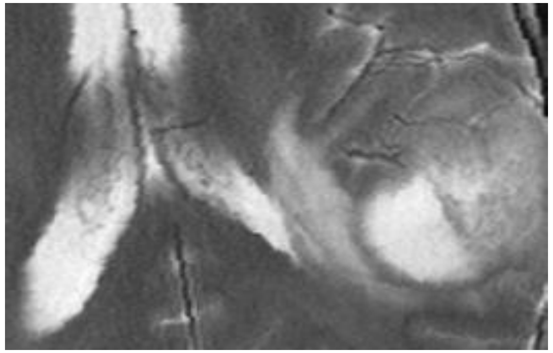

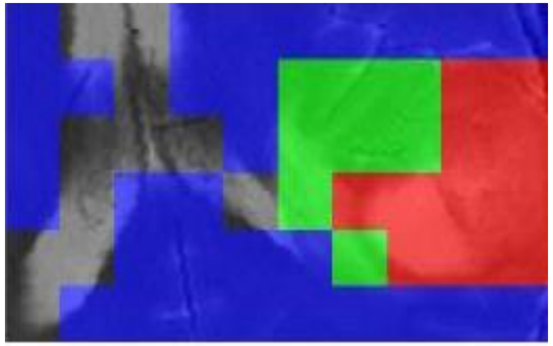

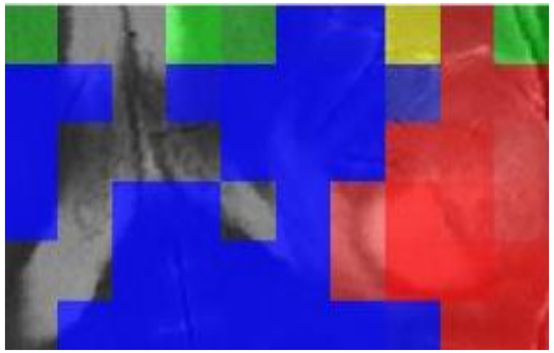

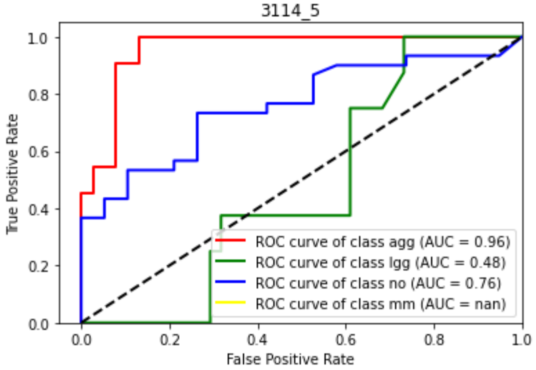

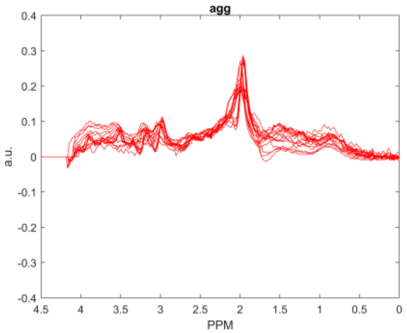

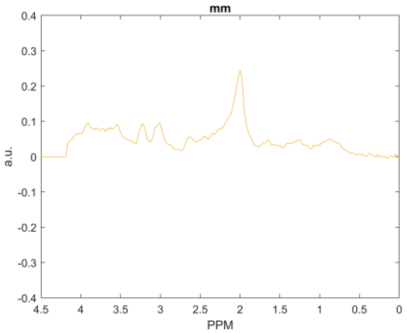

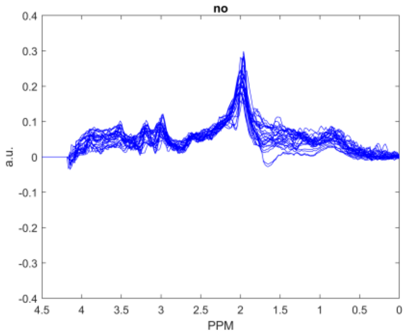

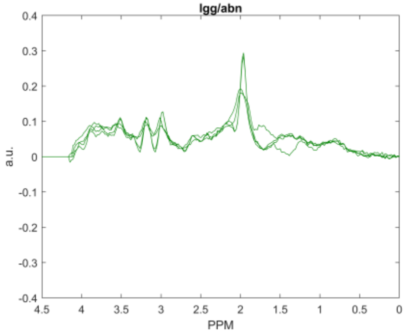

eT3114, S6(10x6)
Glioblastoma. BER=0.50, STI=0.87

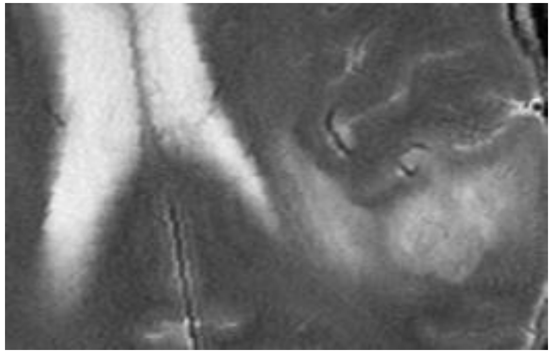

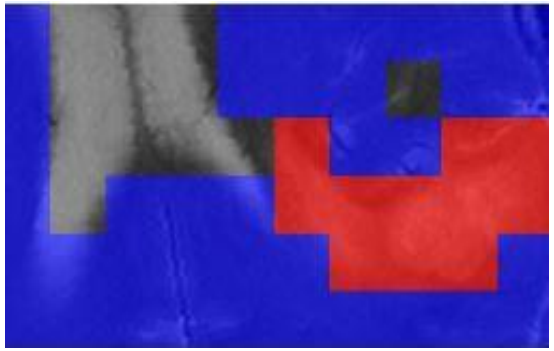

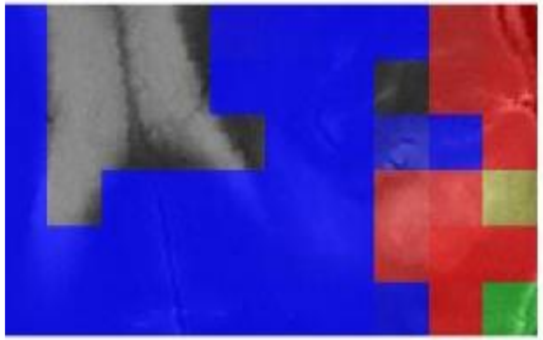

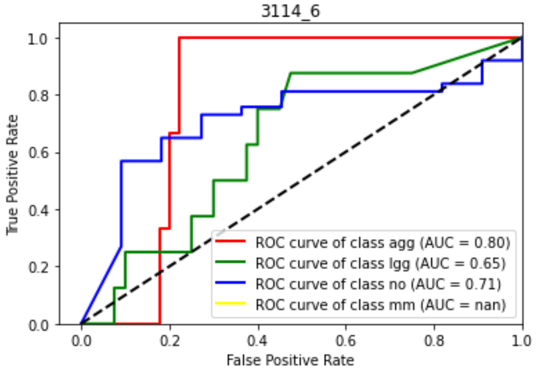

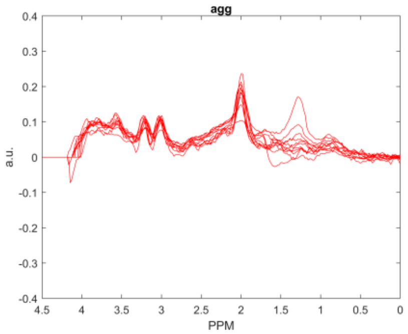

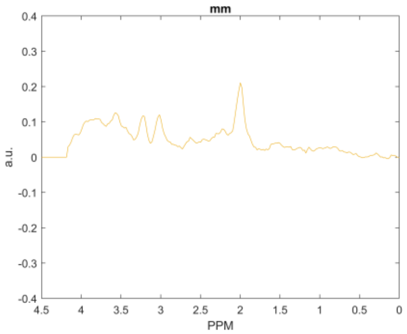

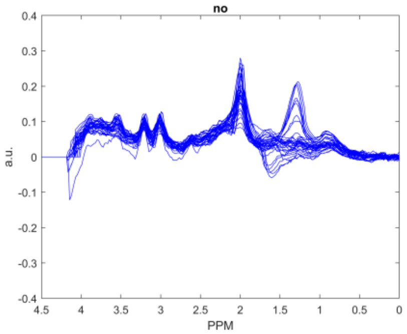

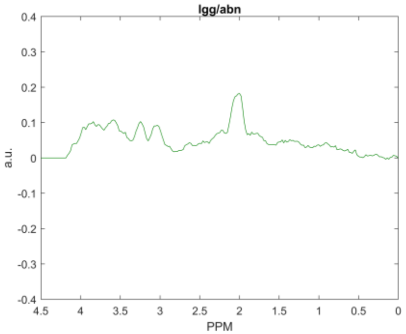

eT3403, S1(10x10)
Glioblastoma. BER=0.7, STI=0.2

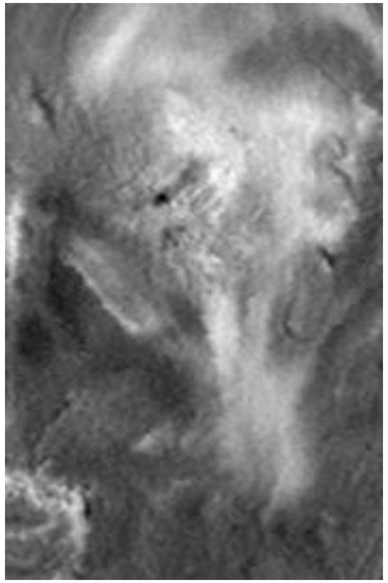

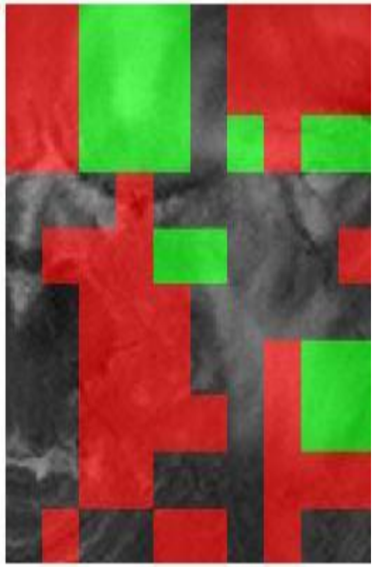

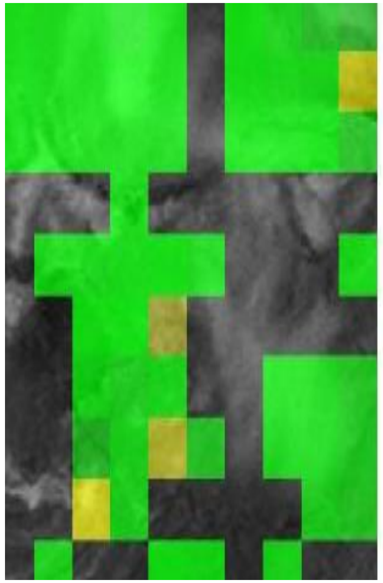

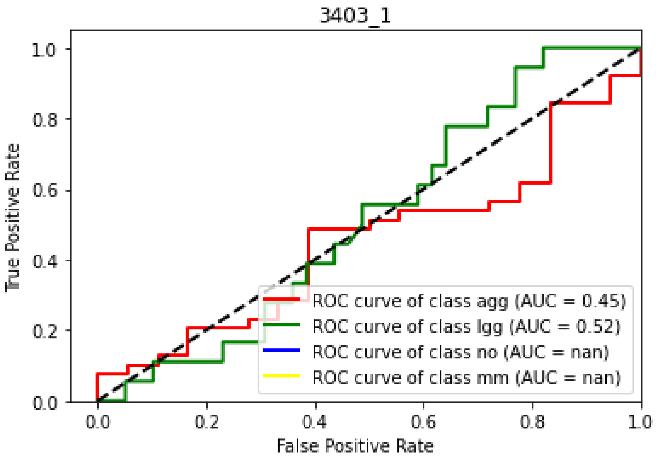

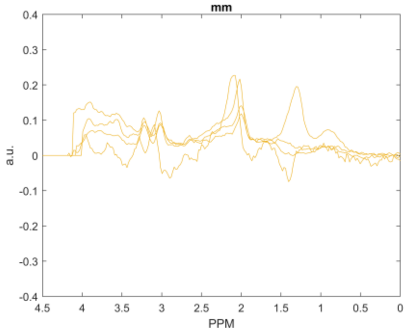

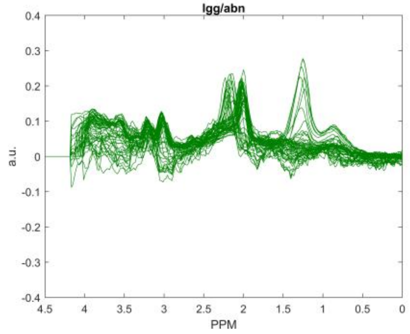

eT3403, S2(10x10)
Glioblastoma. BER=0.5, STI=0.7

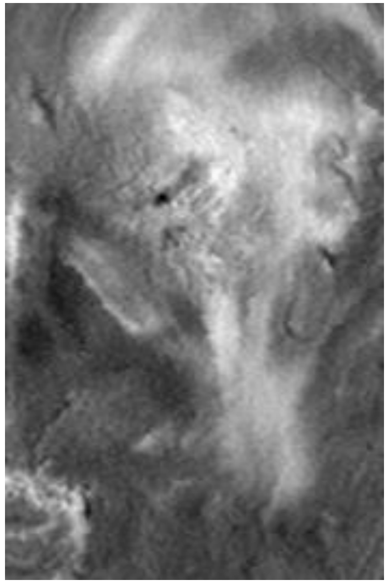

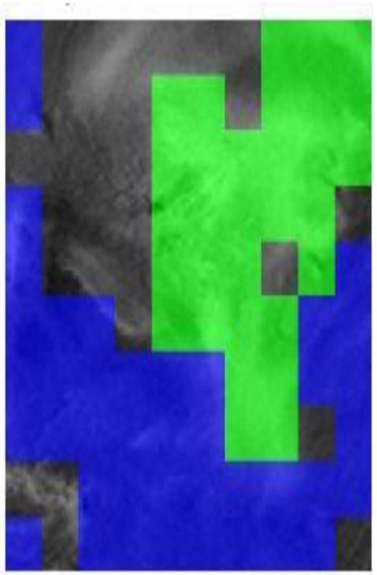

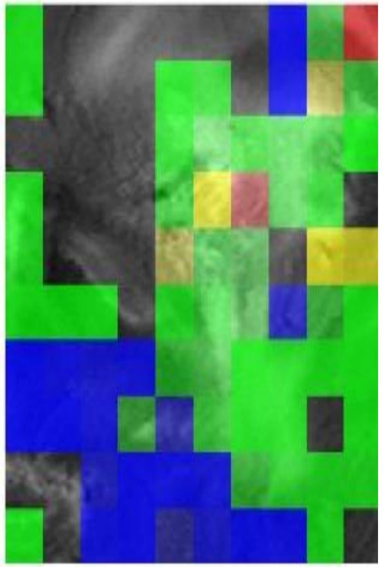

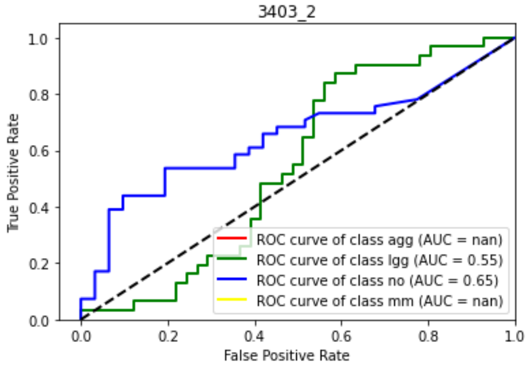

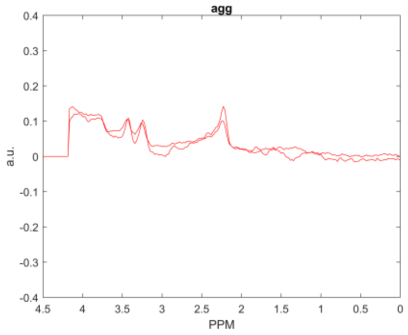

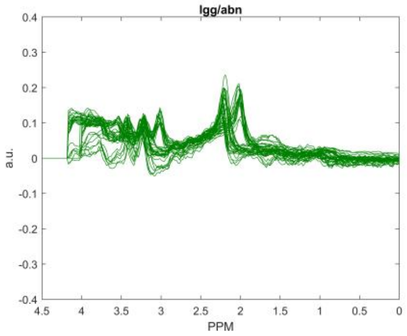

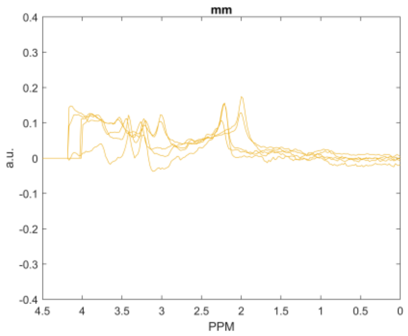

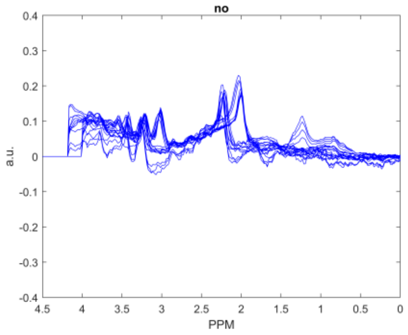

eT3403, S3(10x10)
Glioblastoma BER=0.3, STI=0.6

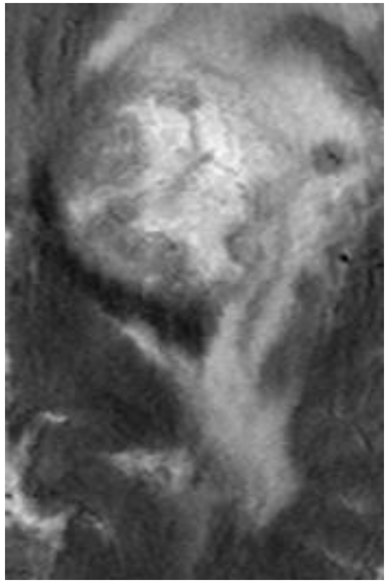

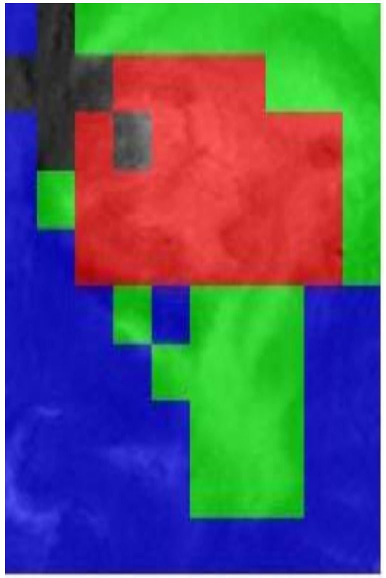

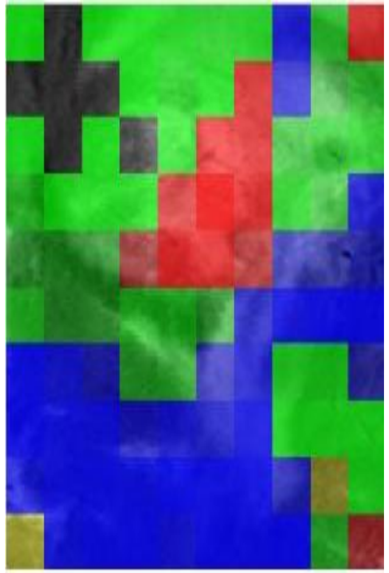

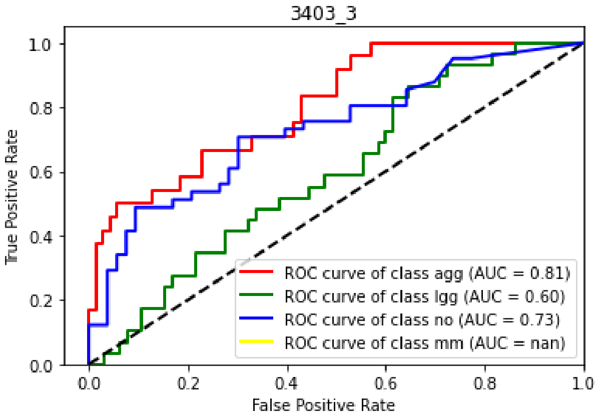

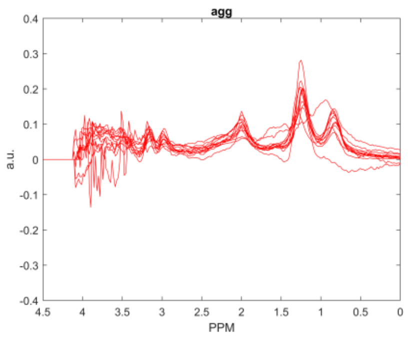

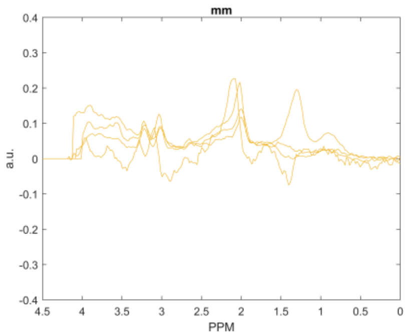

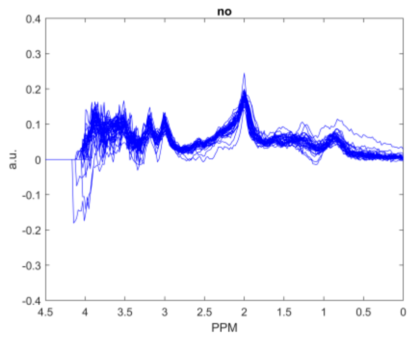

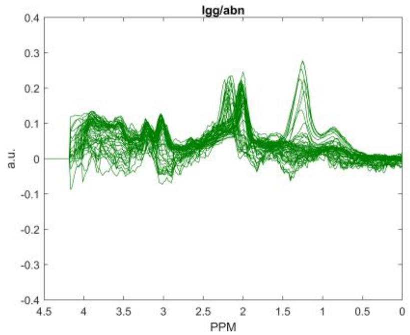

eT3403, S4(10x10)
Glioblastoma. BER=0.4, STI=0.65

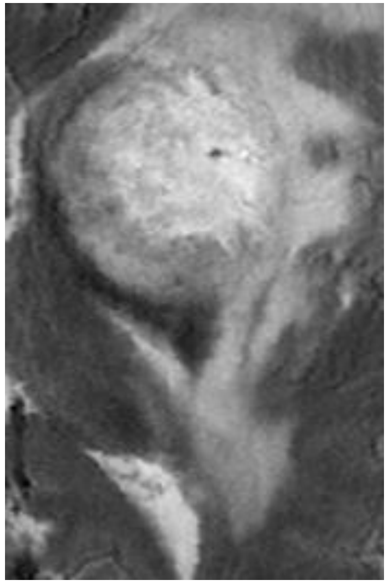

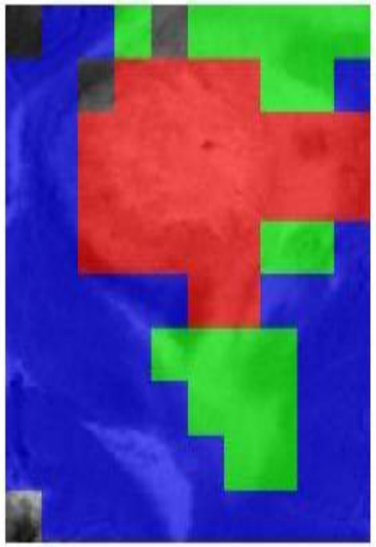

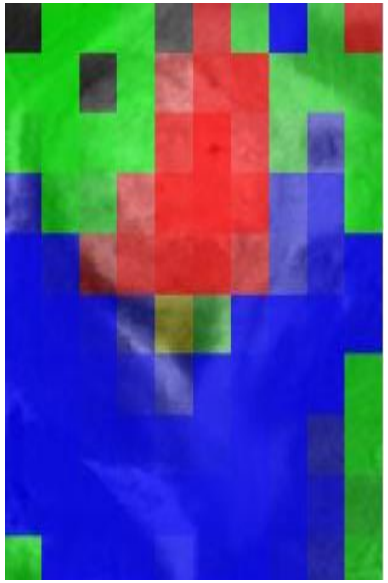

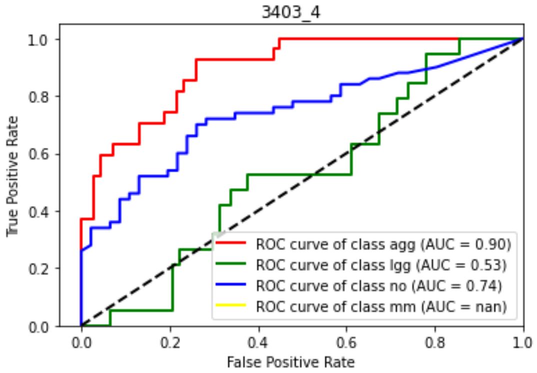

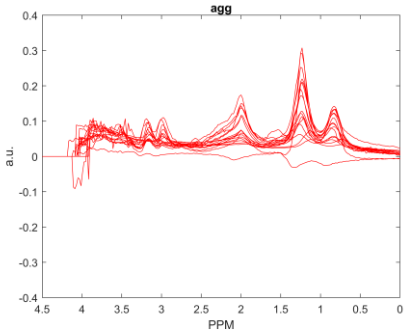

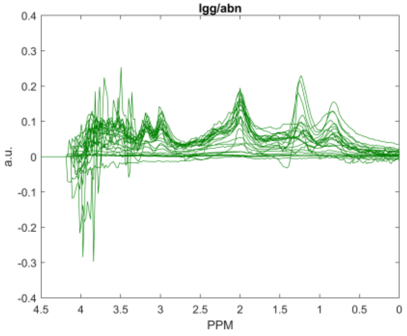

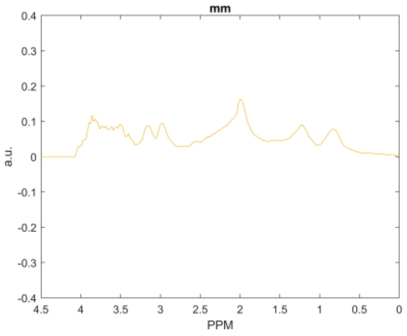

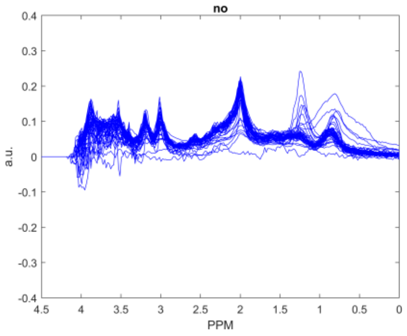

eT3403, S5(10x10)
Glioblastoma. BER=0.5, STI=0.4

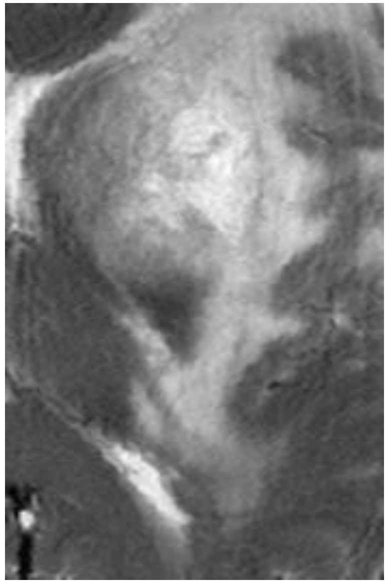

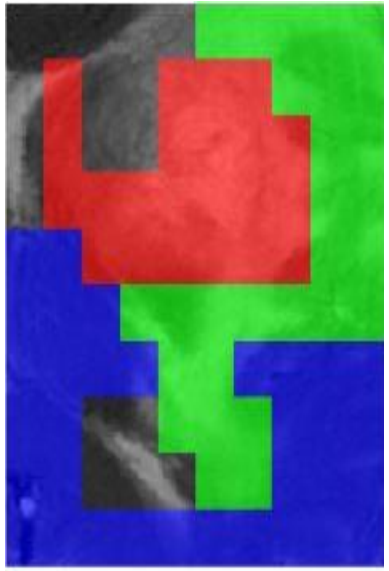

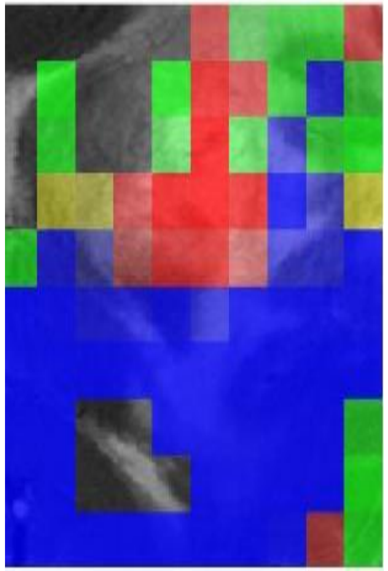

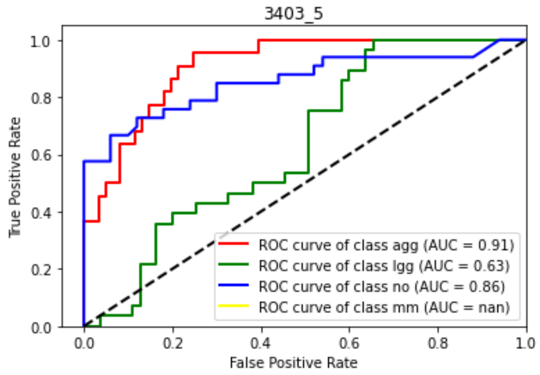

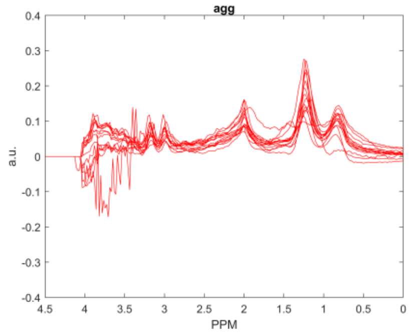

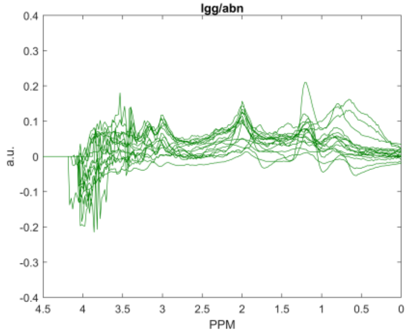

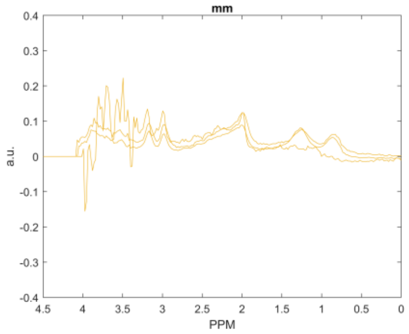

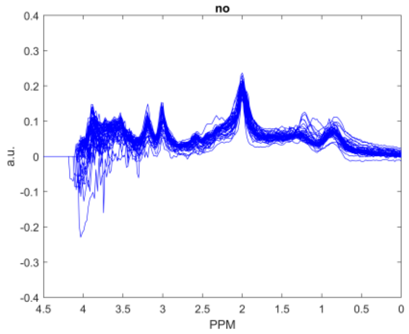

eT3403, S6(10x10)
Glioblastoma. BER=0.8, STI=0.2

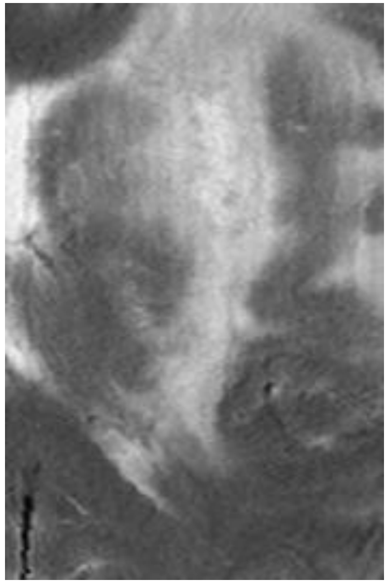

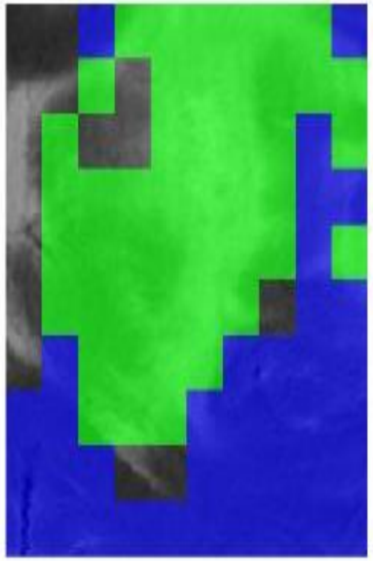

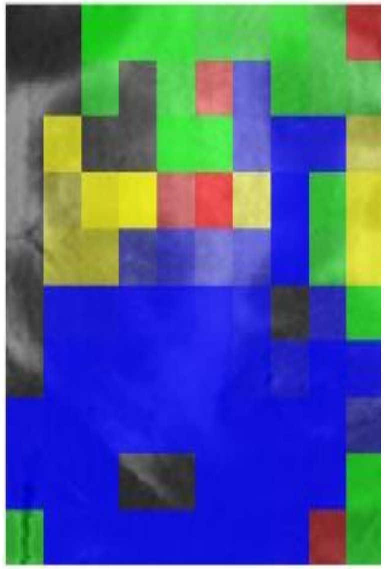

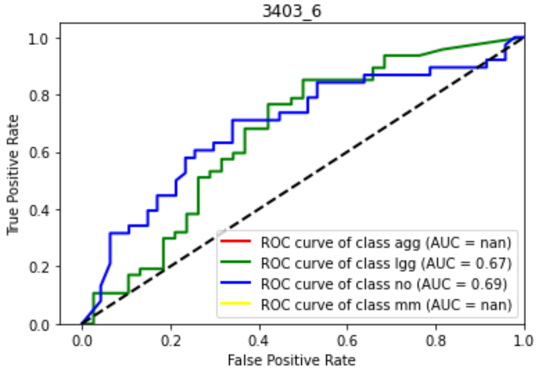

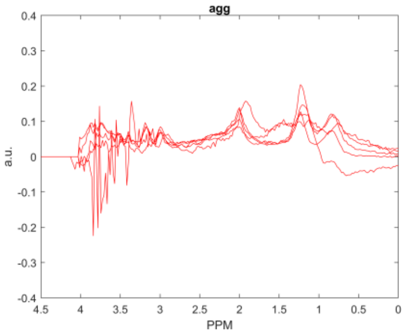

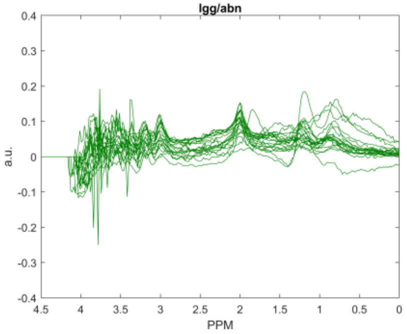

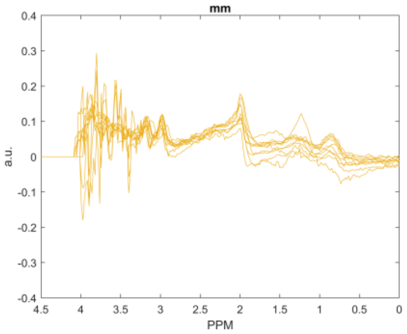

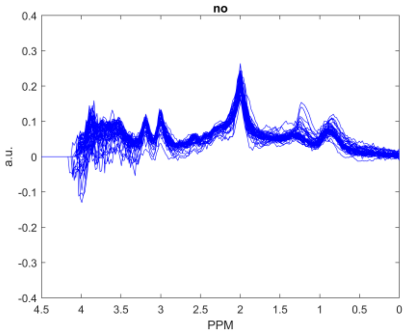

eT3403, S7(10x10)
Glioblastoma. BER=0.5, STI=0.6

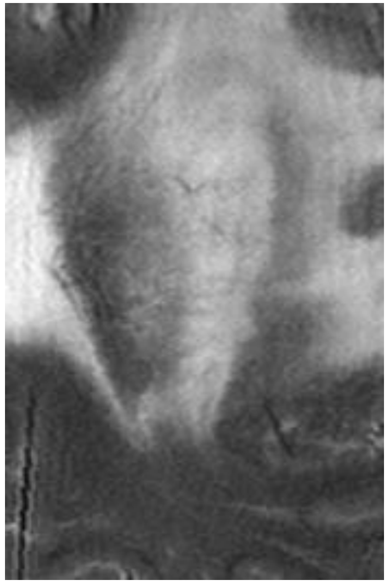

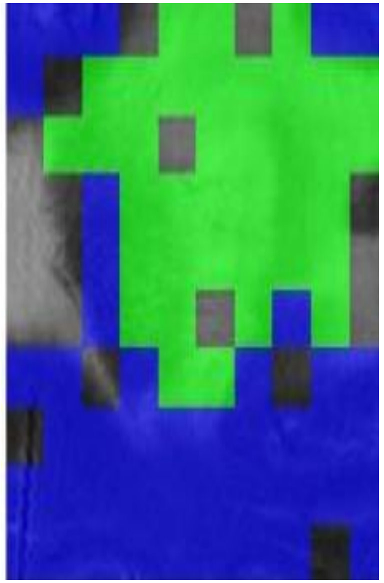

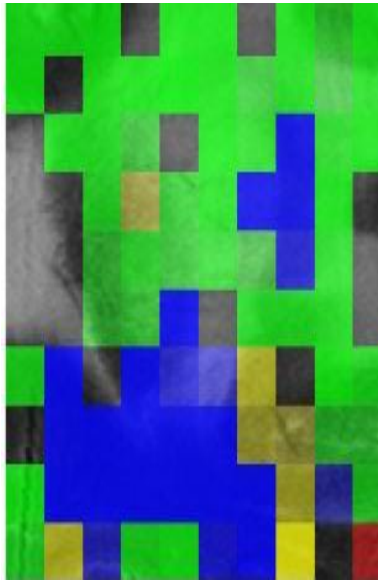

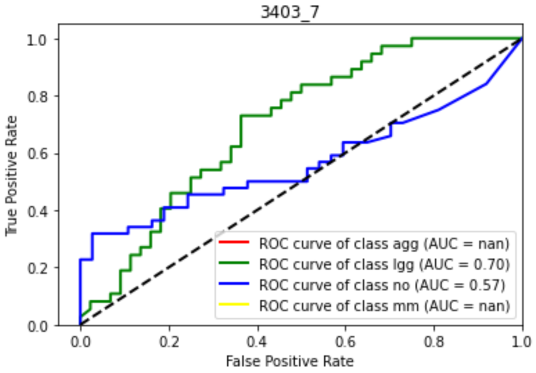

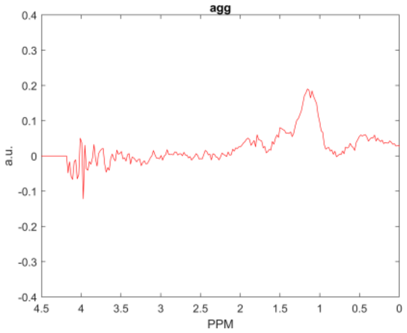

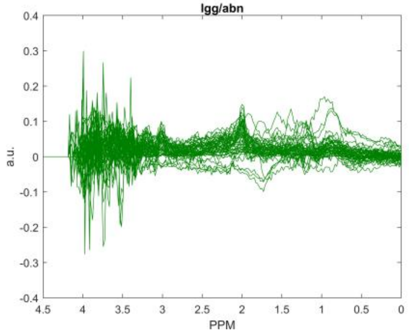

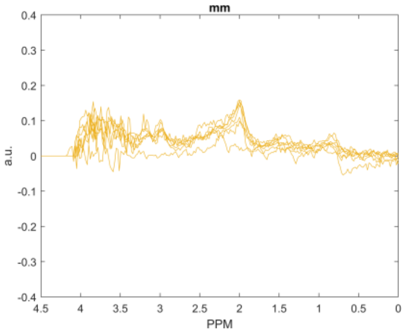

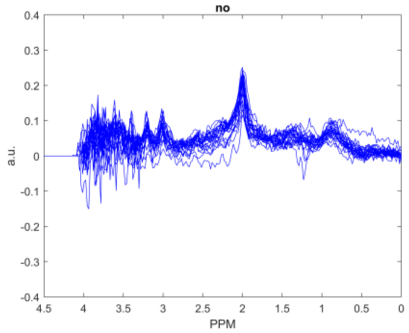

eT3001(20x20)
Metastasis. BER=0.31, STI=1.0

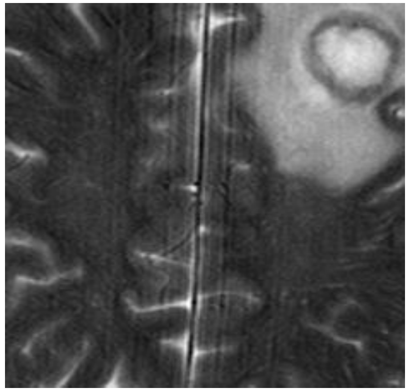

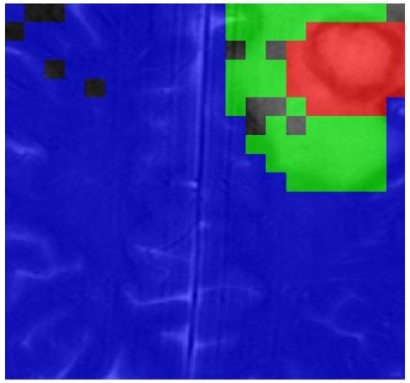

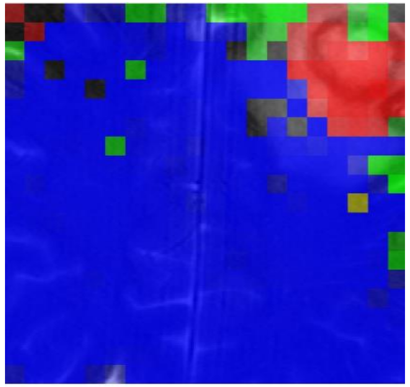

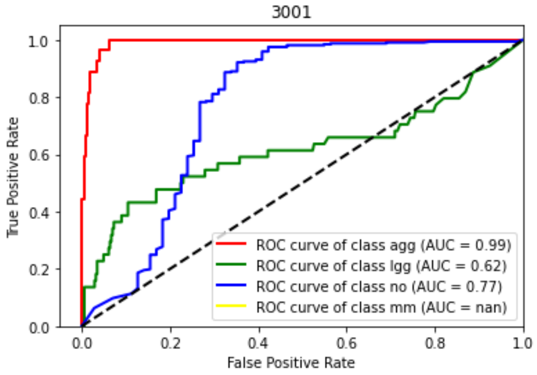

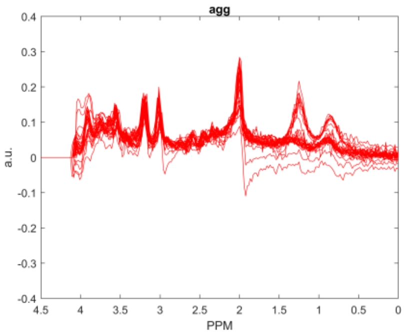

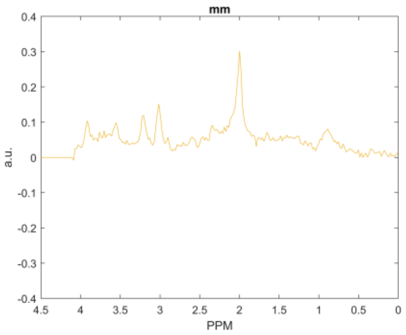

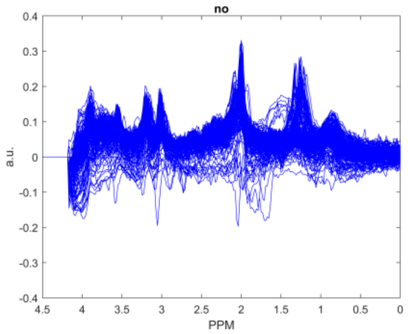

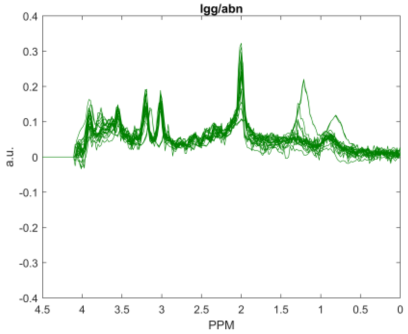

eT3115, S1(10x8)
Metastasis. BER=0.51, STI=0.75

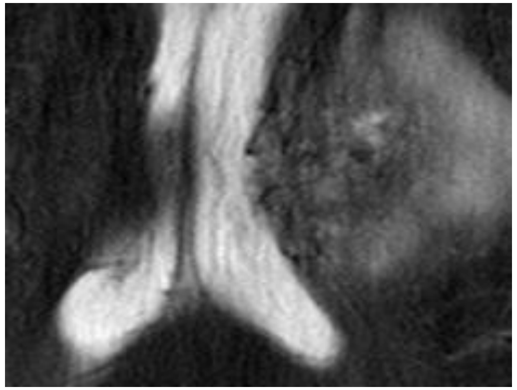

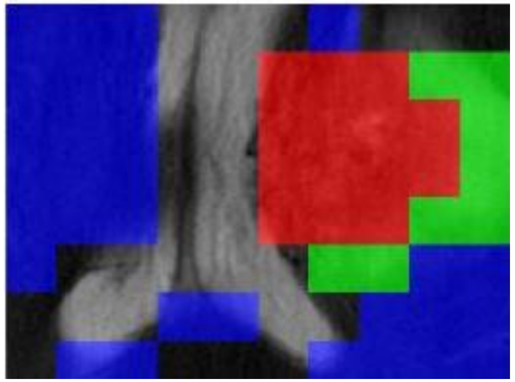

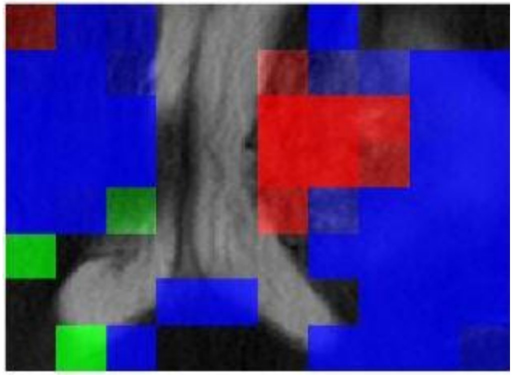

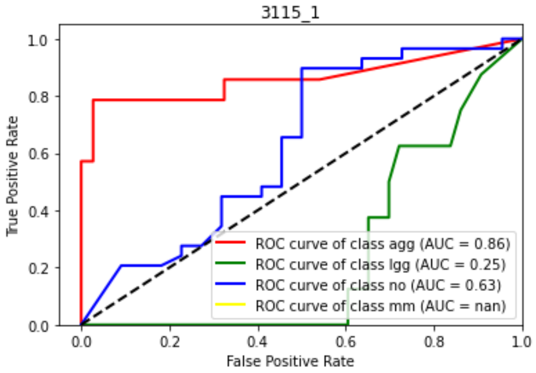

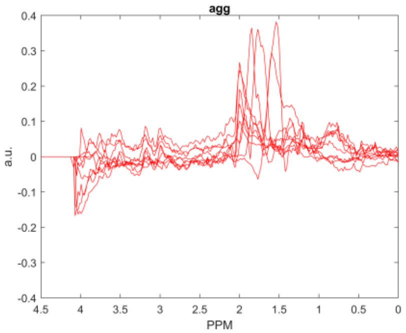

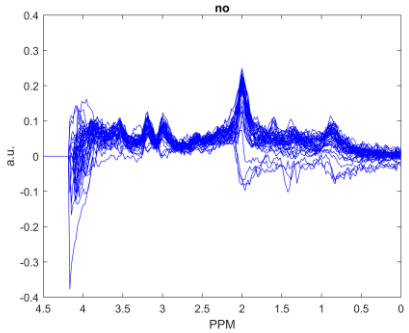

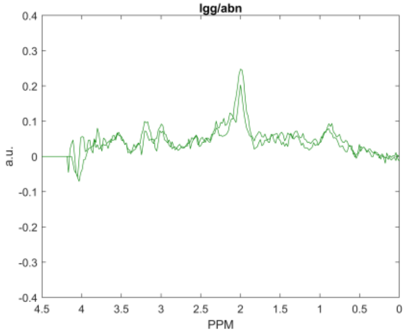

eT3115, S2(10x8)
Metastasis. BER=0.54, STI=0.75

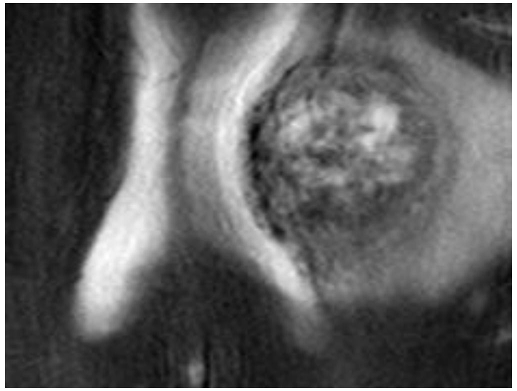

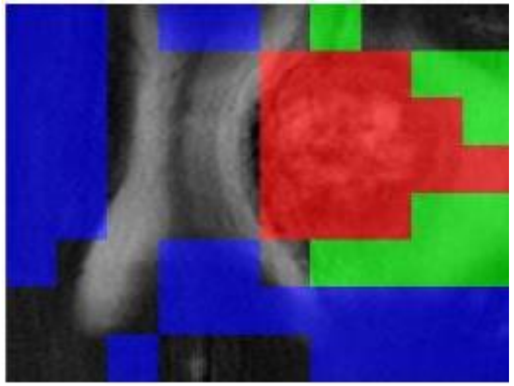

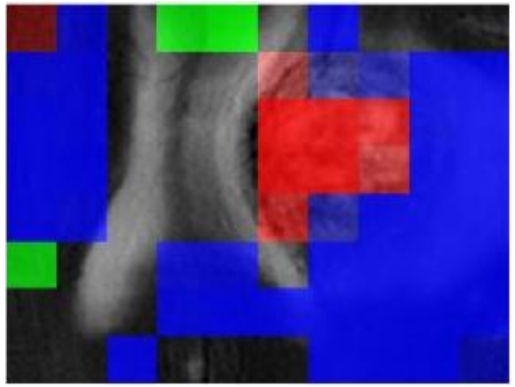

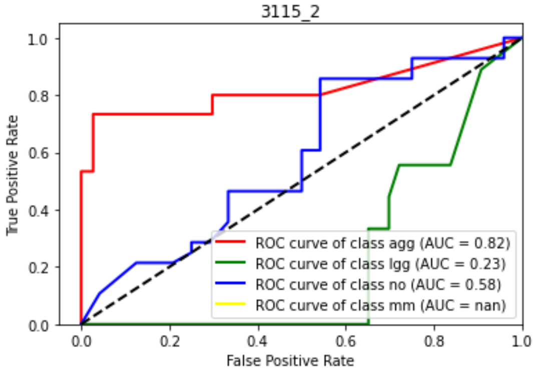

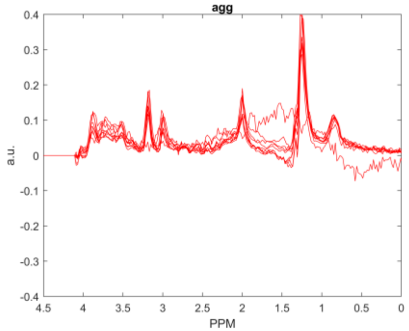

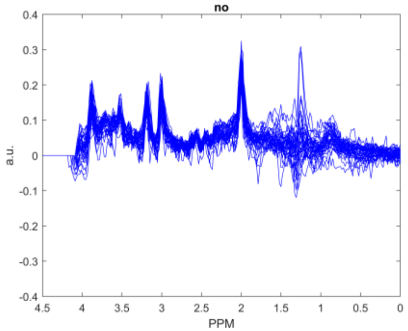

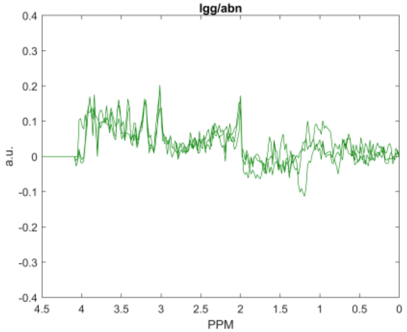

eT3115, S3(10x8)
Metastasis. BER=0.40, STI=0.75

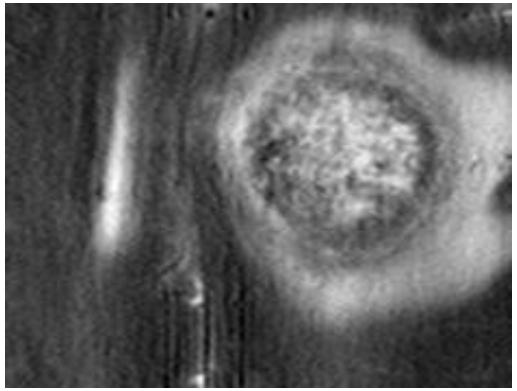

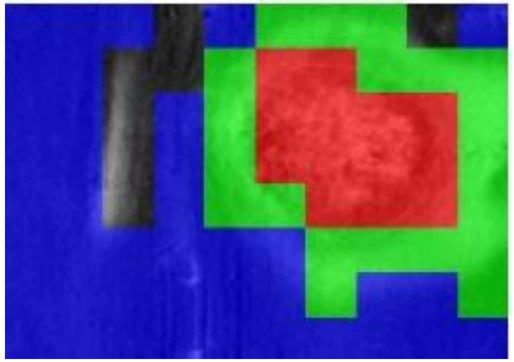

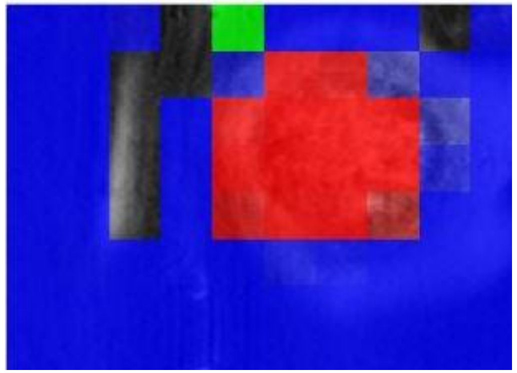

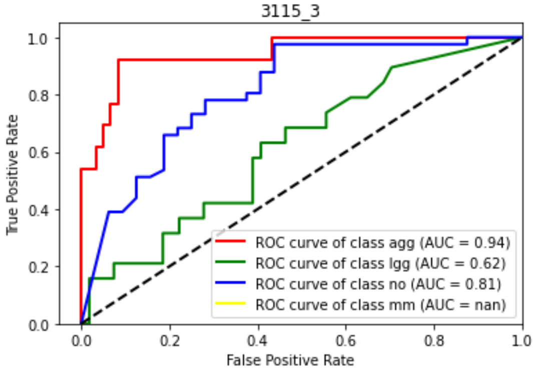

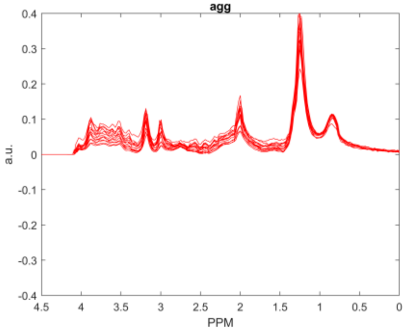

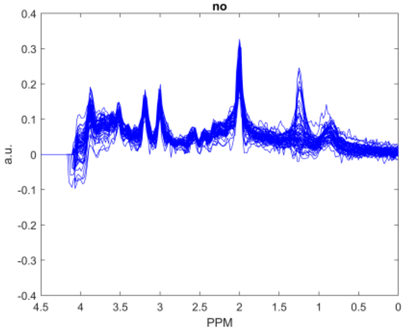

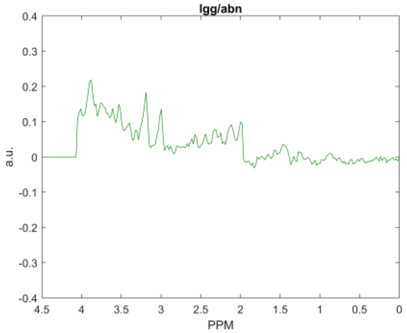

eT3115, S4(10x8)
Metastasis. BER=0.32, STI=0.75

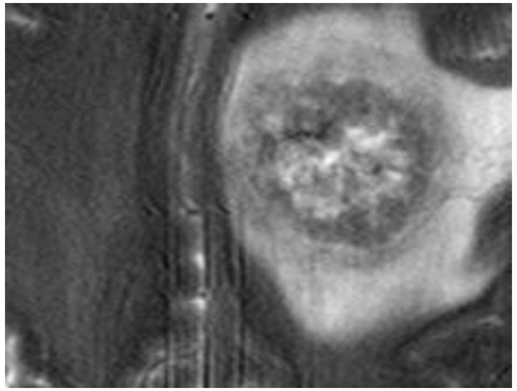

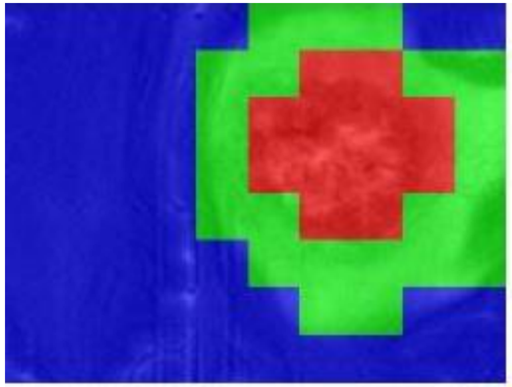

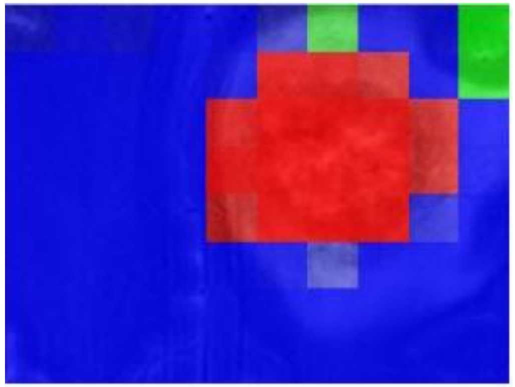

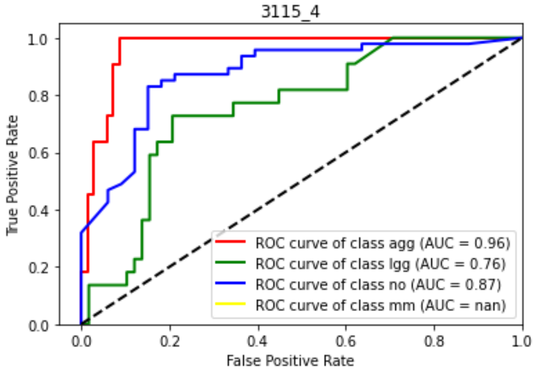

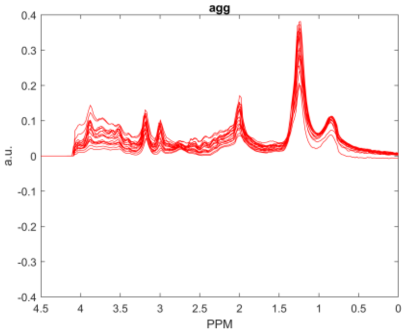

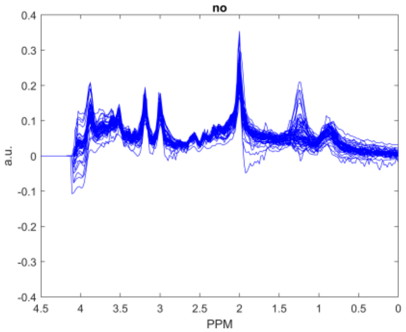

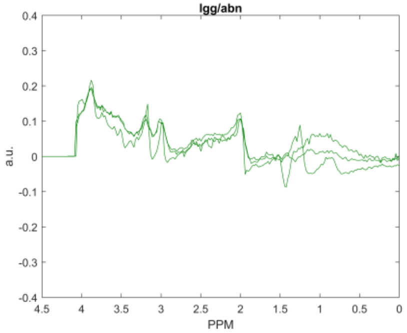

eT3115, S6(10x8)
Metastasis. BER=0.33, STI=0.75

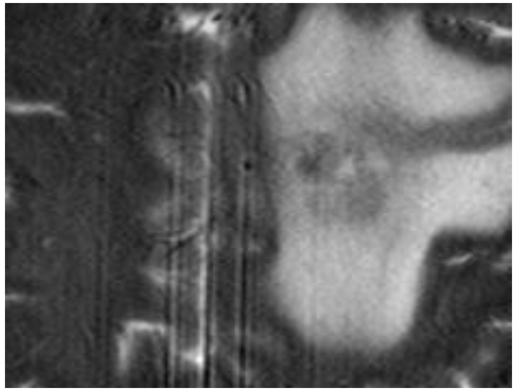

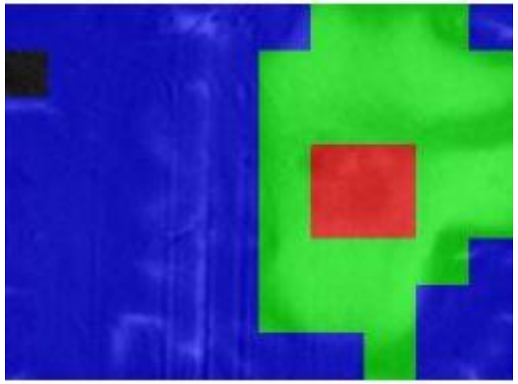

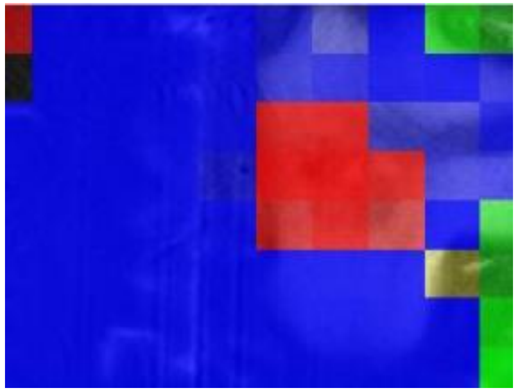

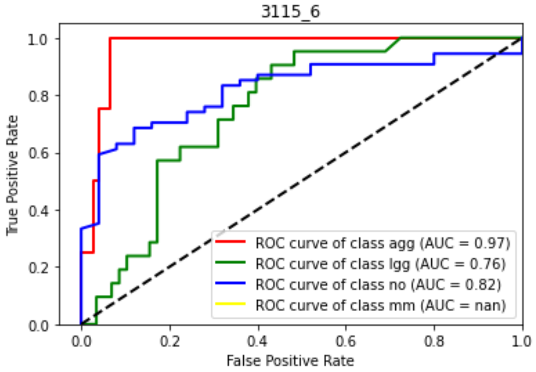

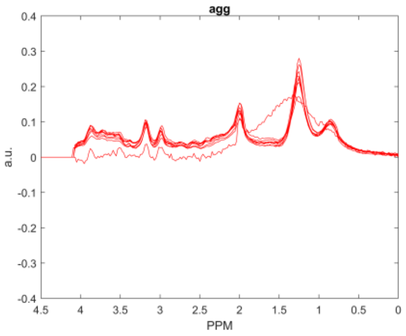

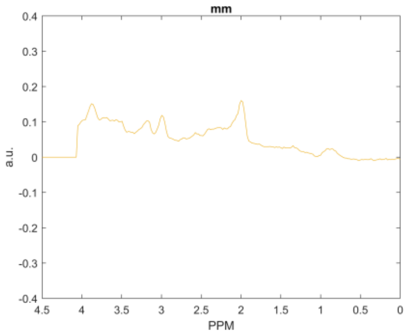

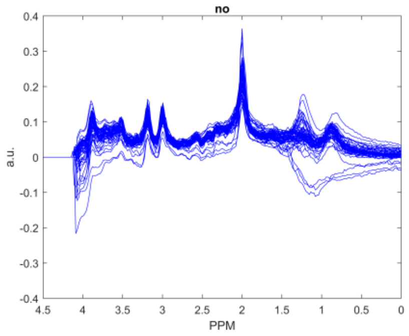

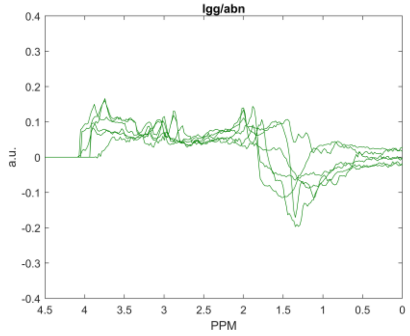

eT3115, S7(10x8)
Metastasis. BER=0.54, STI=0.75

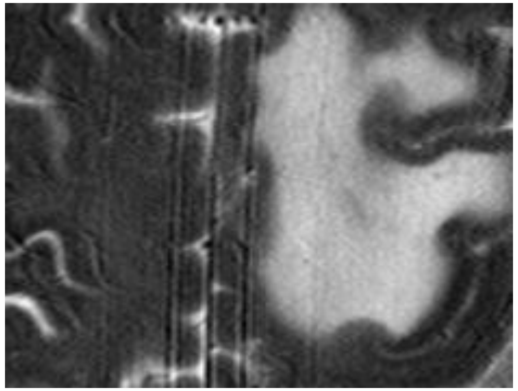

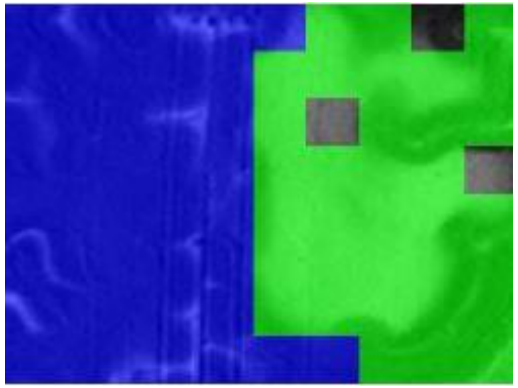

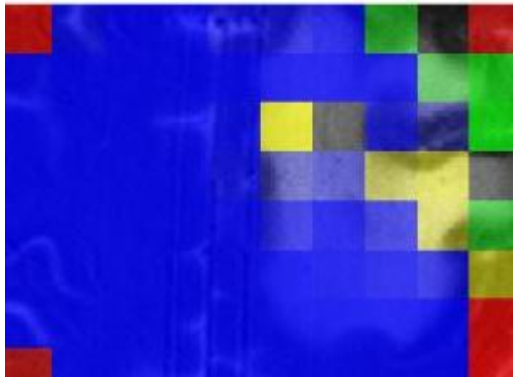

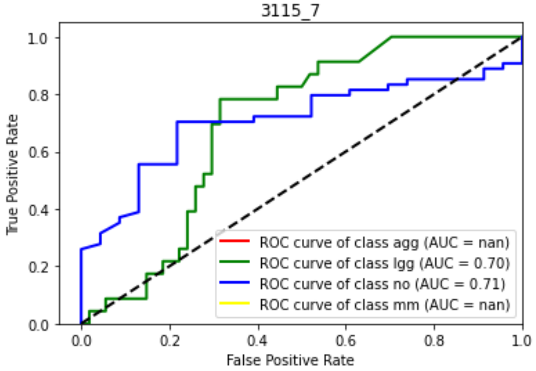

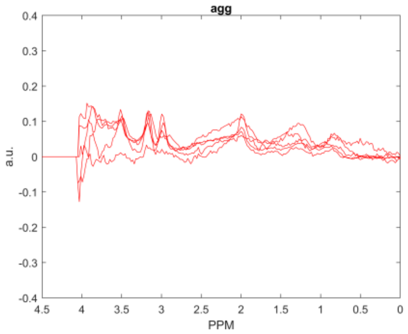

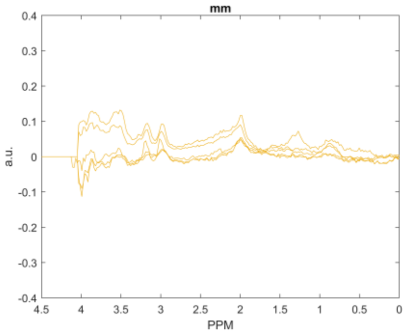

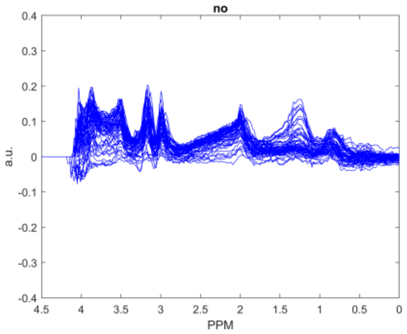

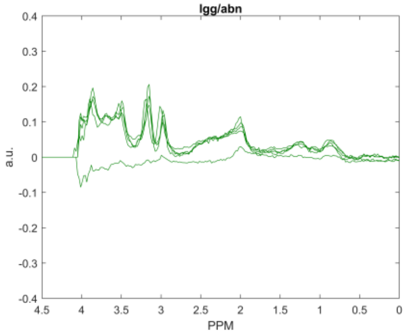

eT3115, S8(10x8)
Metastasis. BER=0.71, STI=0.75

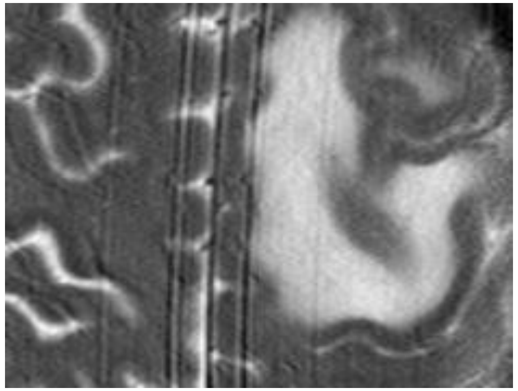

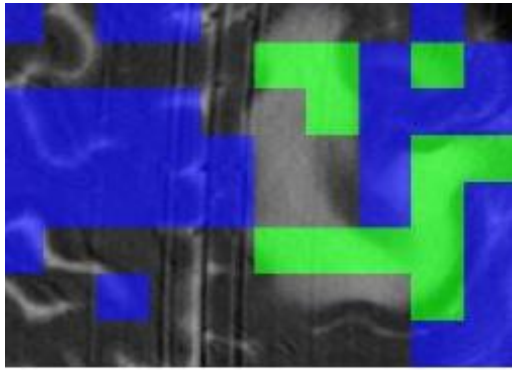

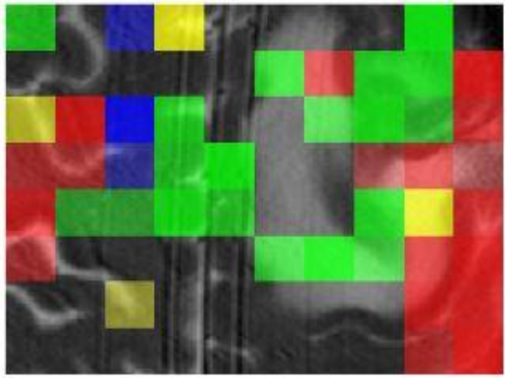

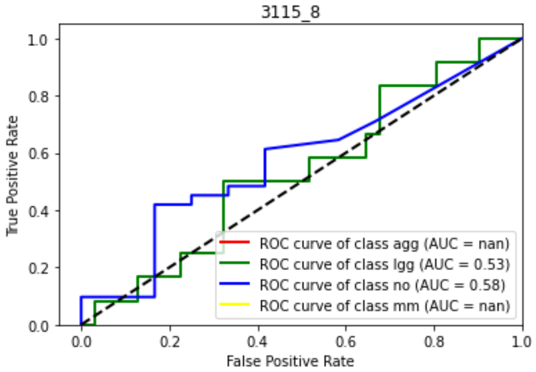

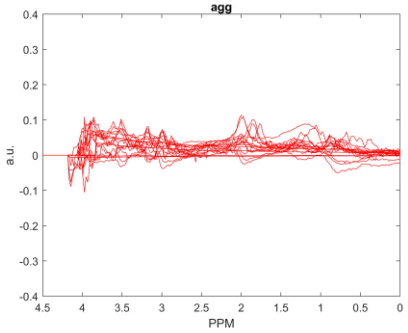

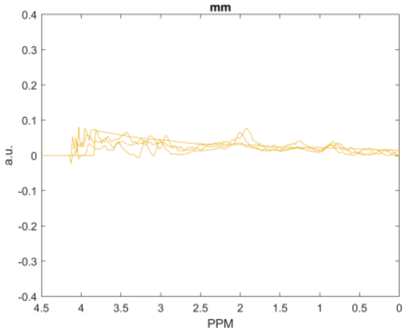

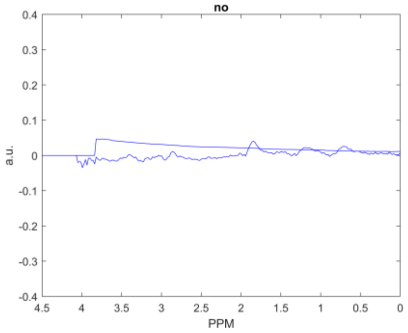

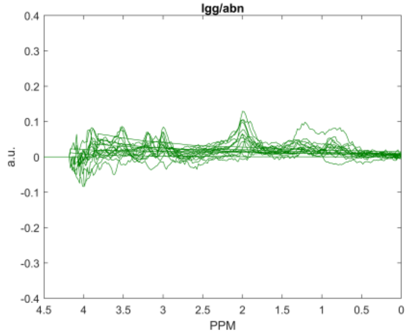

eT2932(20x16)
Atypical Meningioma. BER=0.24, STI=0.83

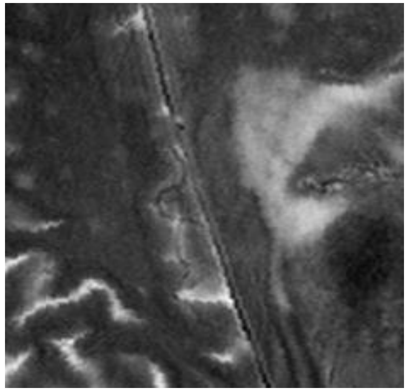

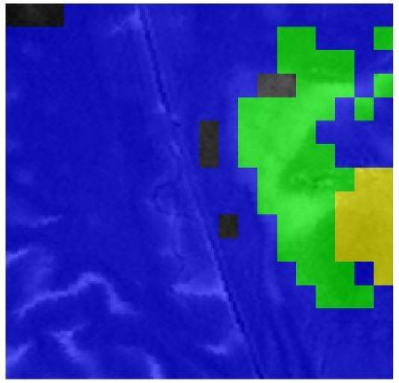

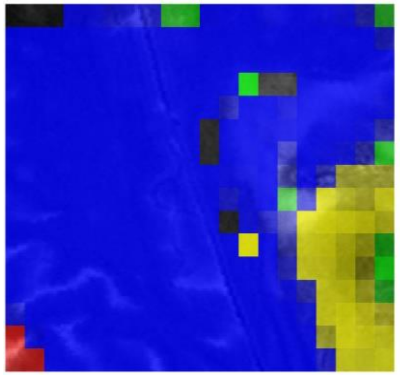

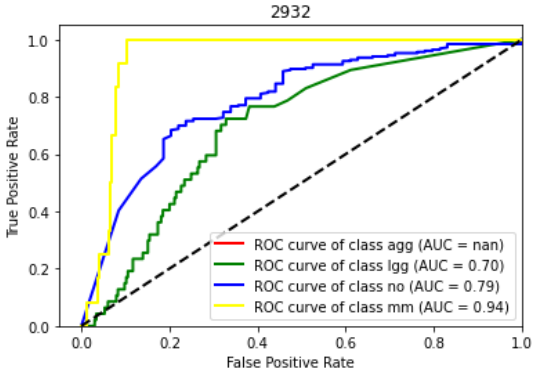

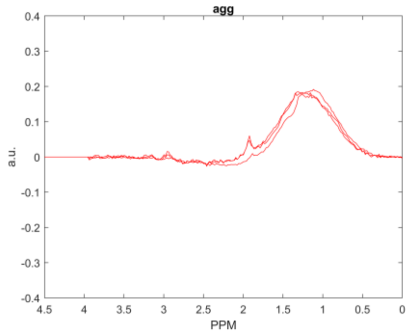

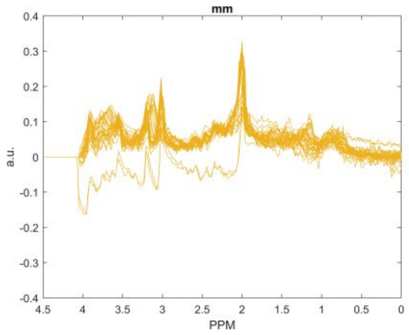

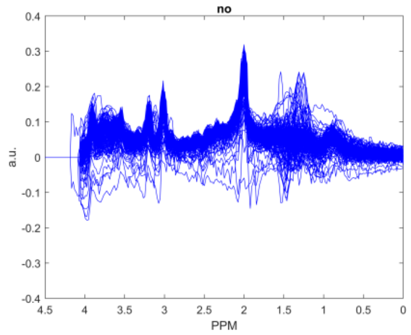

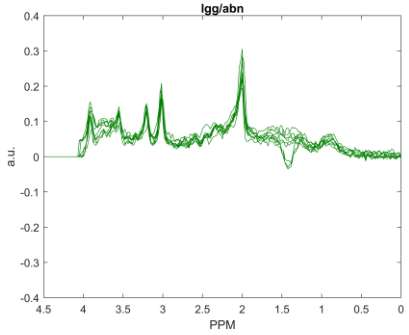

eT2945(18x20)
Meningioma. BER=0.39, STI=0.96

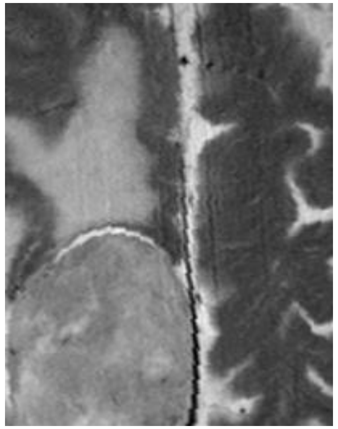

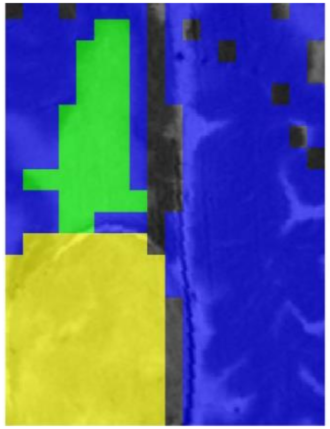

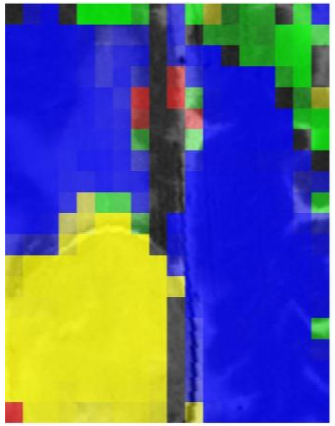

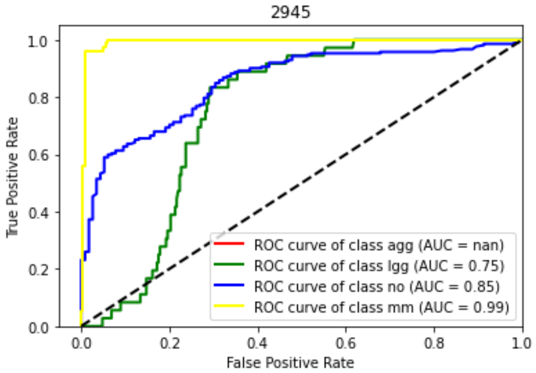

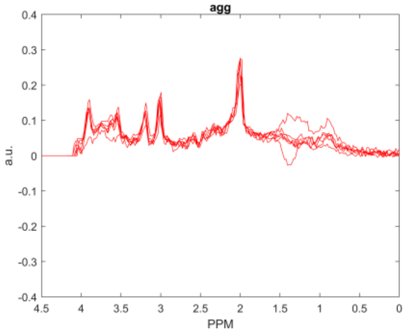

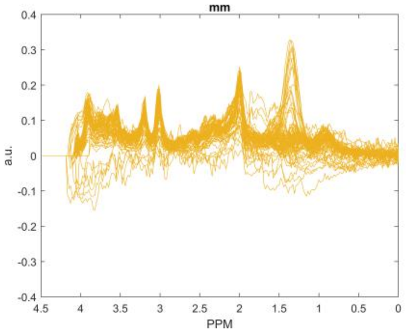

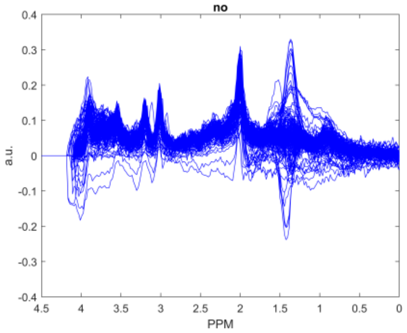

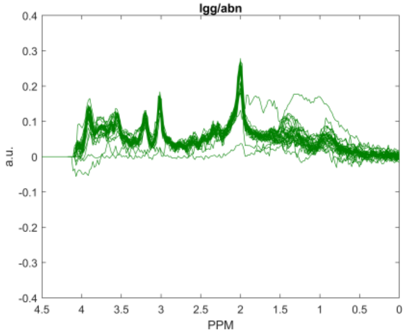

eT2948(18x15)
Meningioma. BER=0.55, STI=0.42

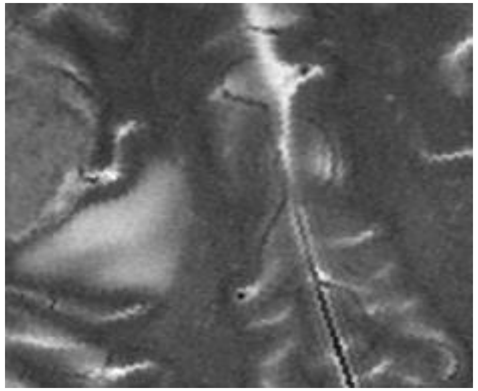

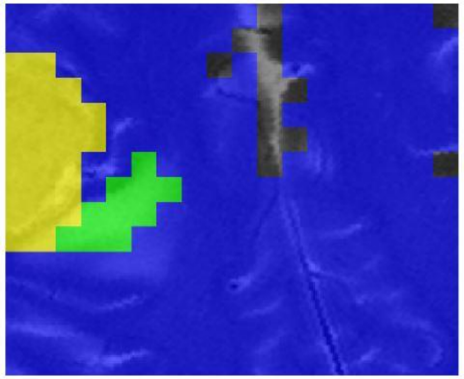

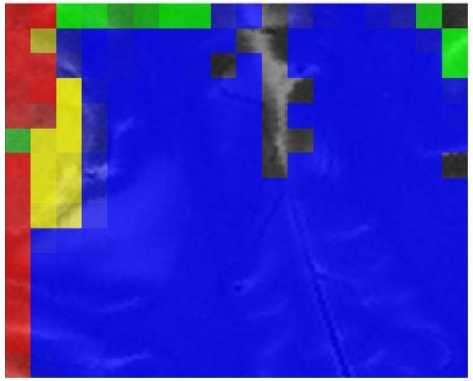

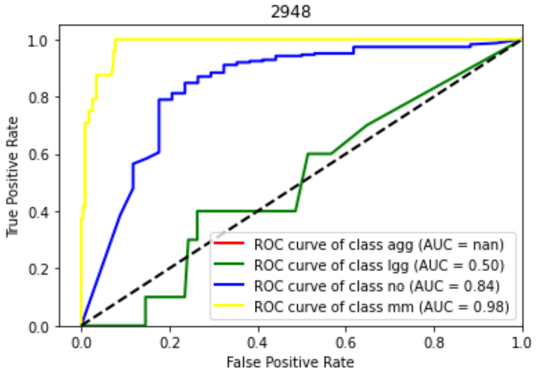

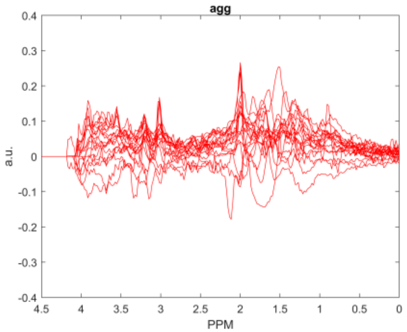

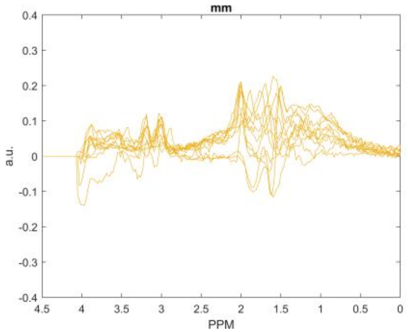

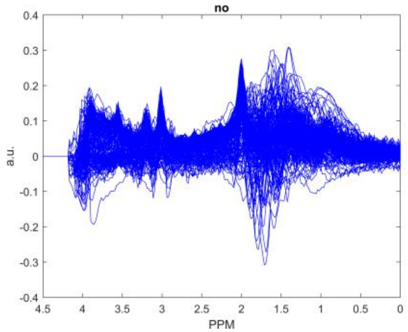

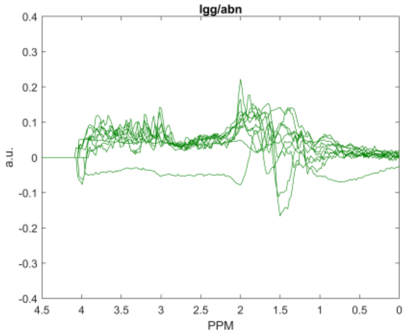

eT3109, S1(10x8)
Meningioma. BER=1.0, STI=0.0

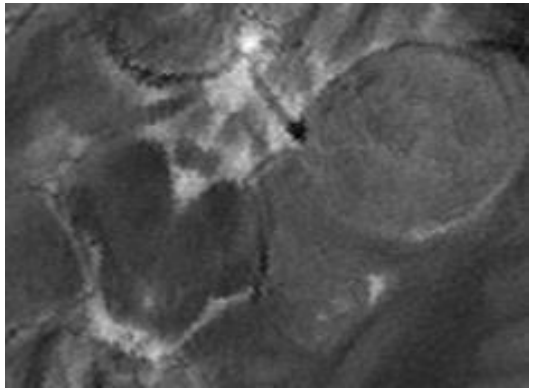

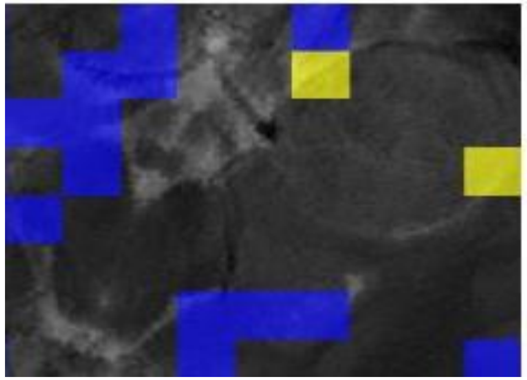

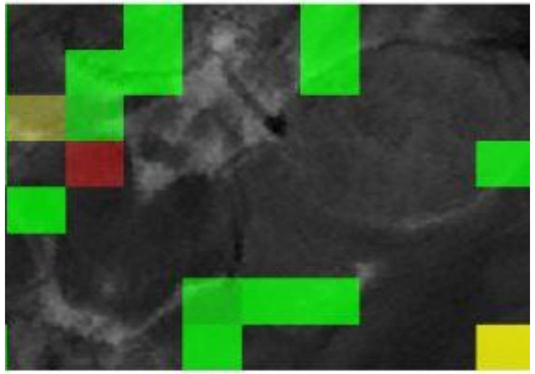

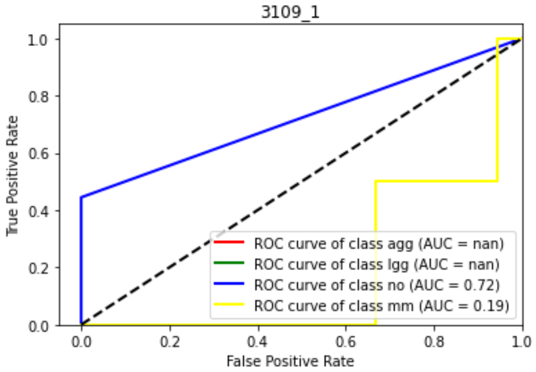

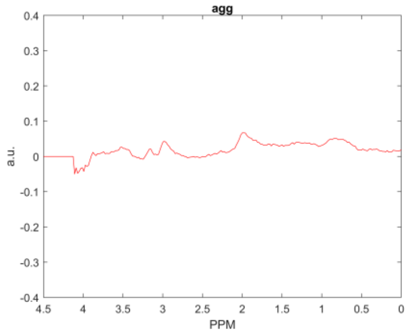

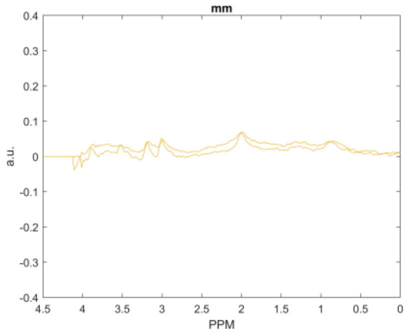

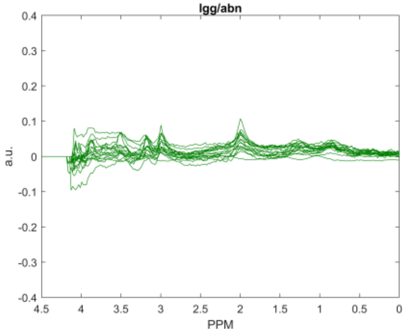

eT3109, S2(10x8)
Meningioma. BER=1.0, STI=0.0

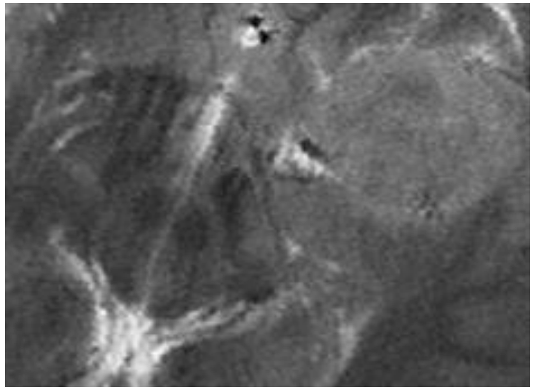

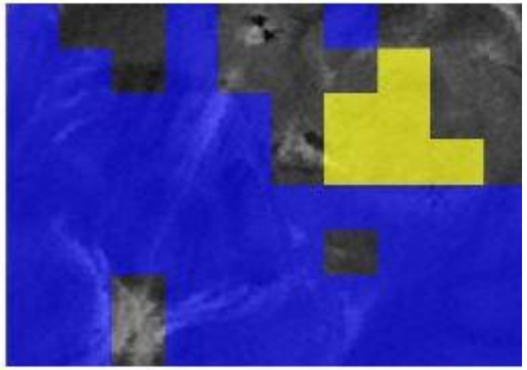

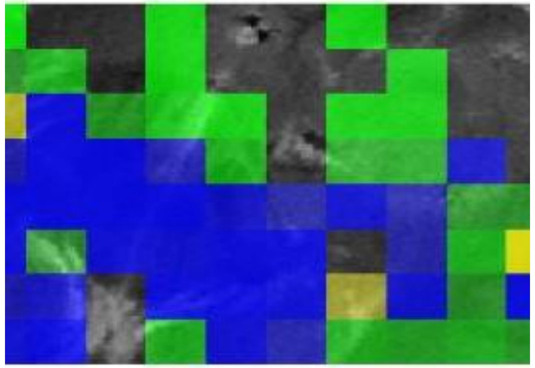

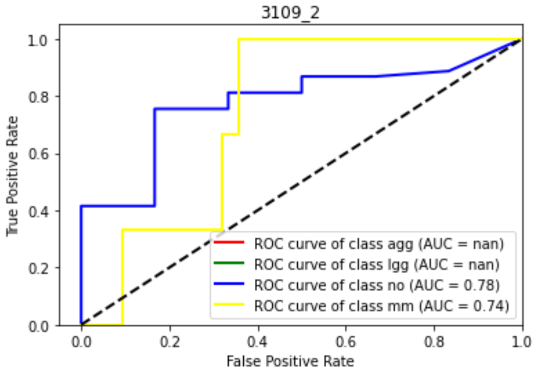

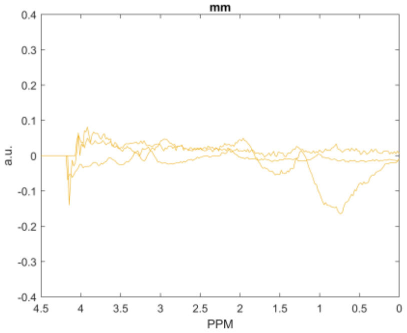

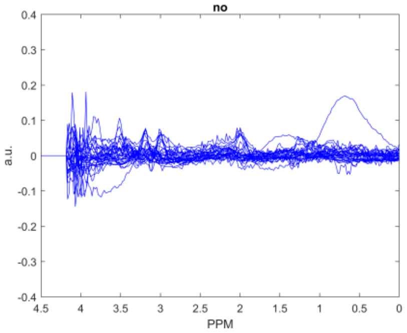

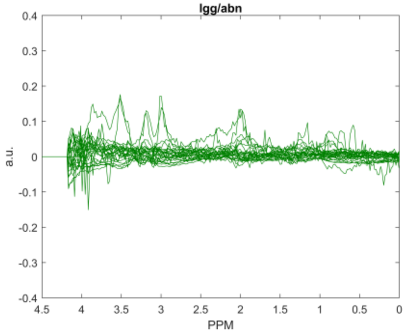

eT3109, S3(10x8)
Meningioma. BER=1.0, STI=0.0

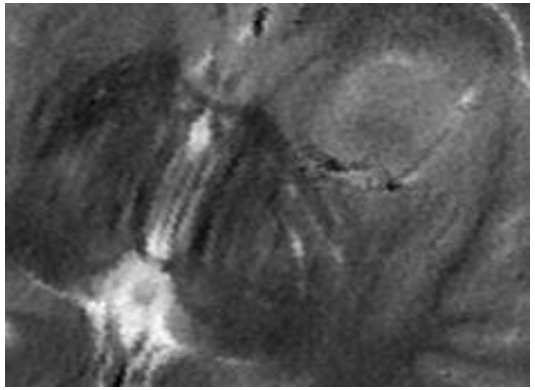

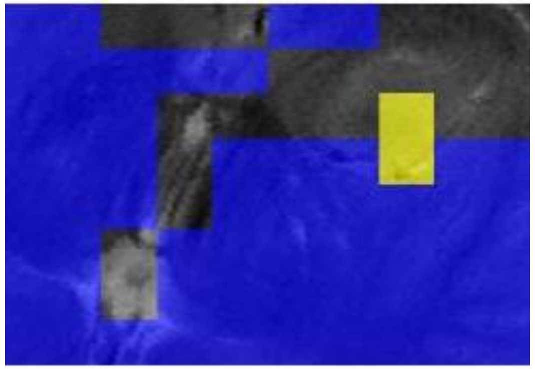

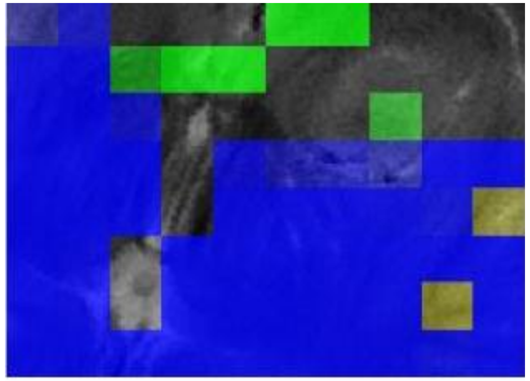

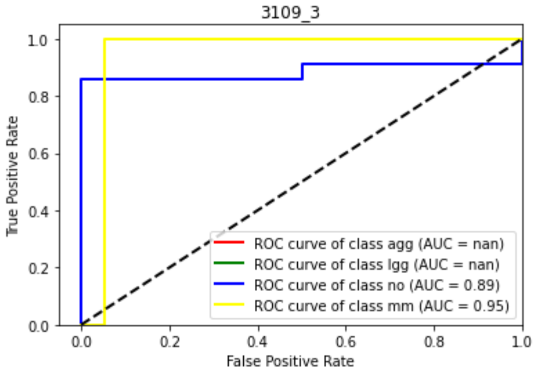

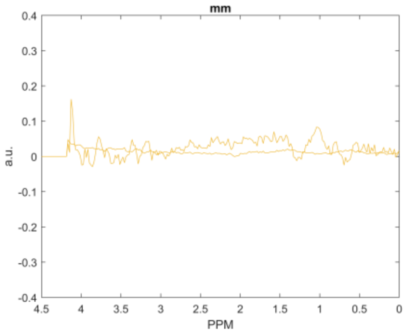

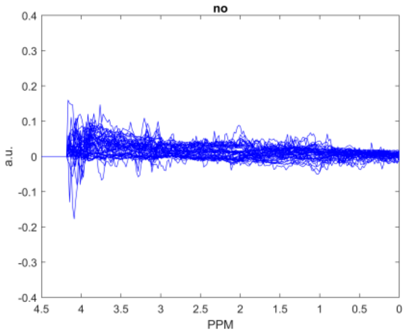

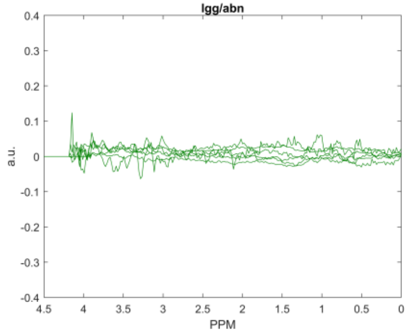

eT3109, S5(10x8)
Meningioma. BER=1.0, STI=0.0

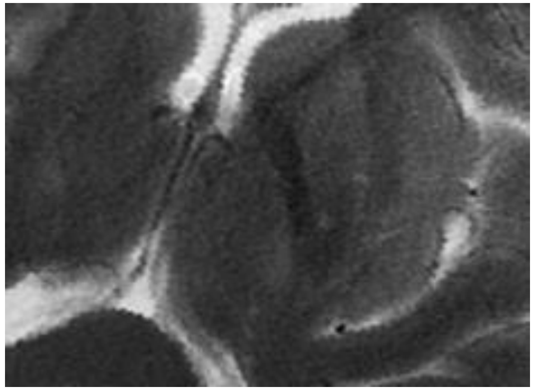

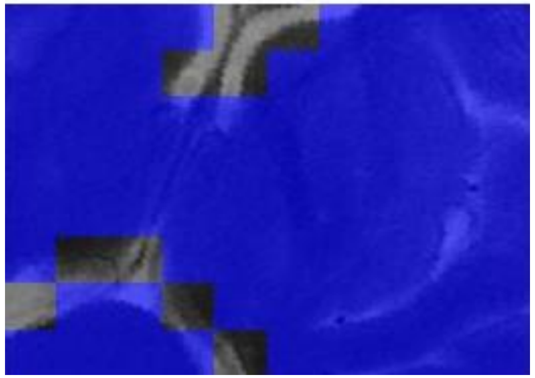

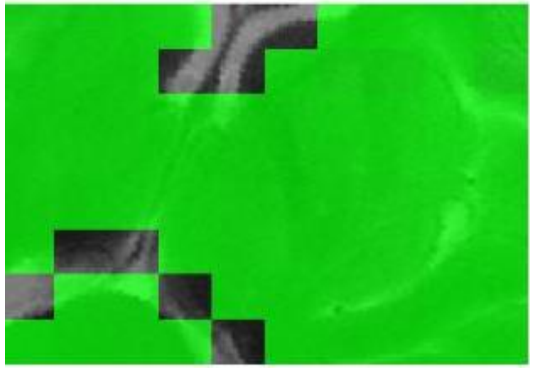

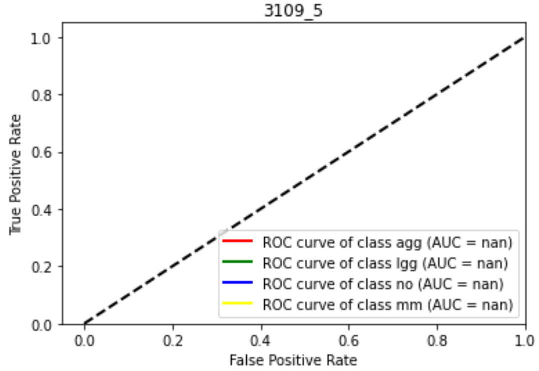

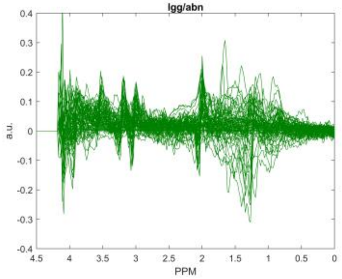

eT3109, S6(10x8)
Meningioma. BER=1.0, STI=0.0

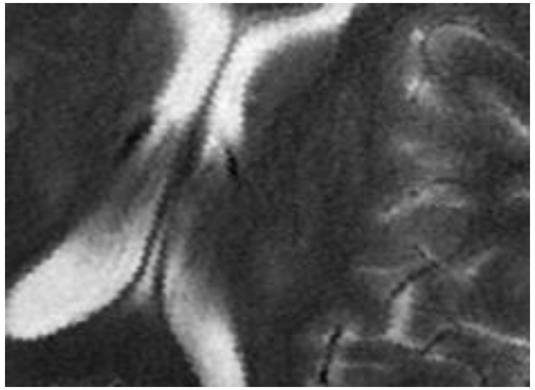

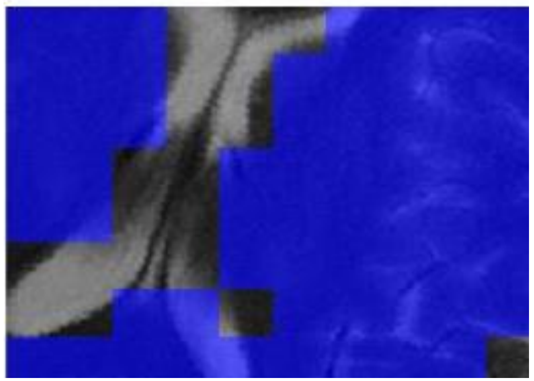

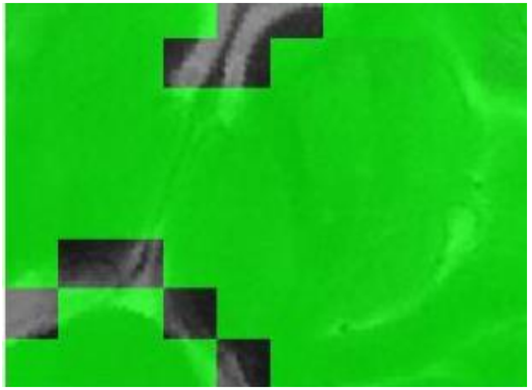

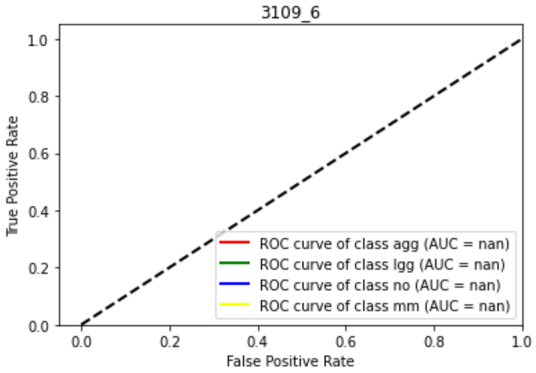

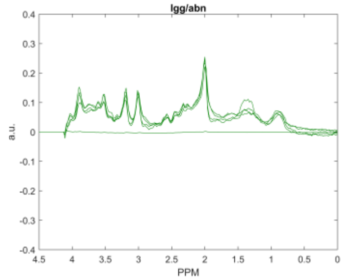

eT3035(18x14)
Diffuse Astrocytoma. BER=0.22, STI=1.0

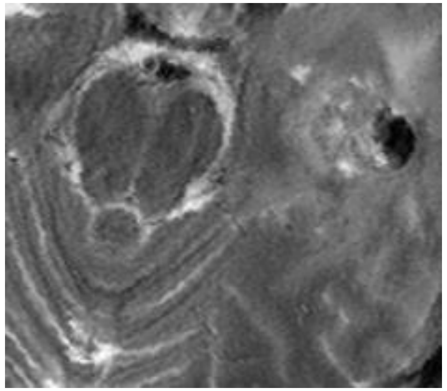

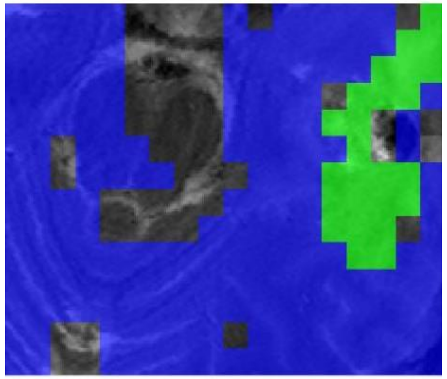

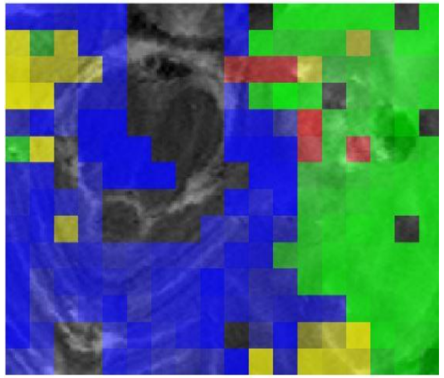

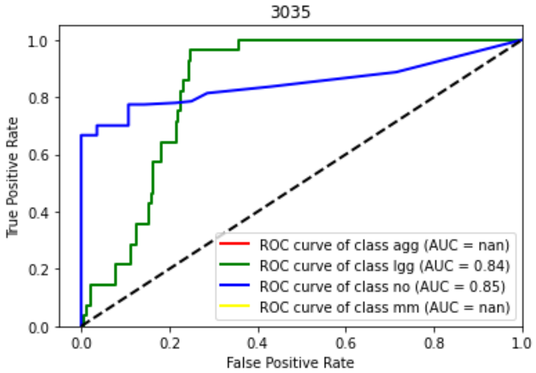

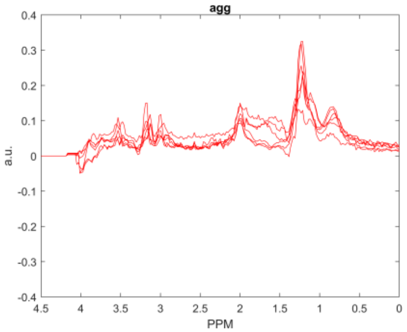

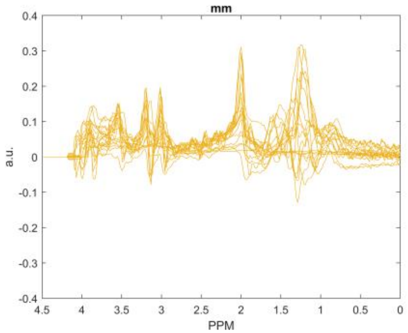

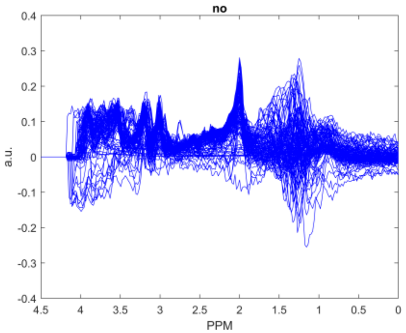

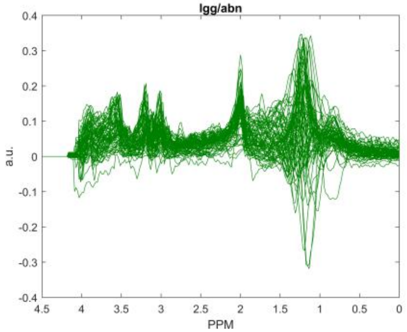

Excluded Cases

eT2491(22x20)
Anaplastic Oligodendroglioma. BER=0.27, STI=Excluded

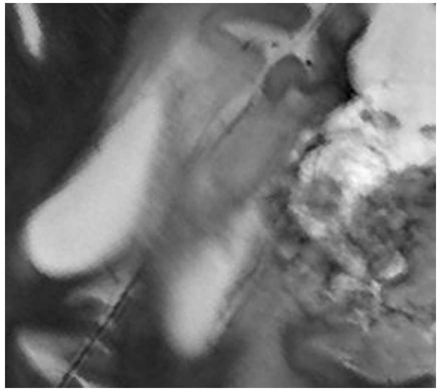

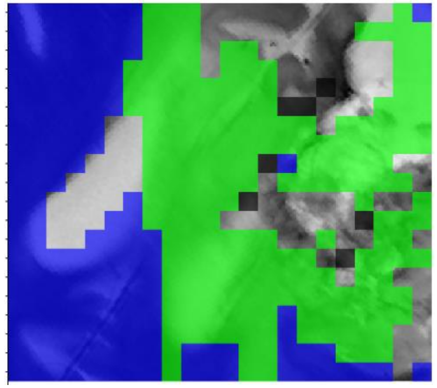

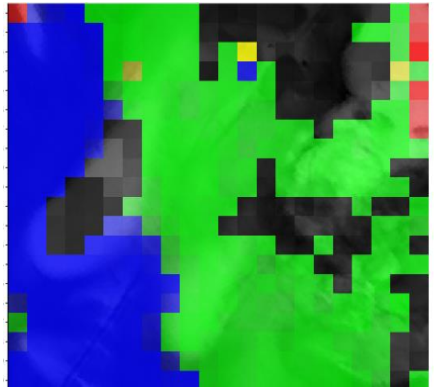

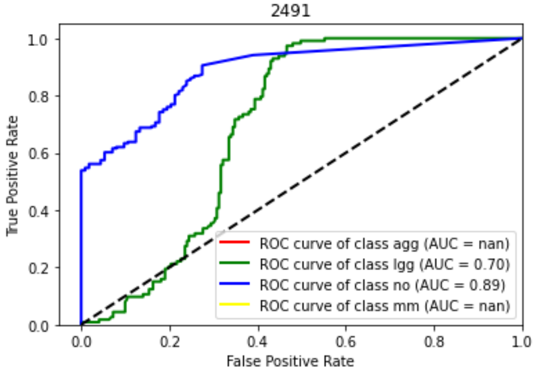

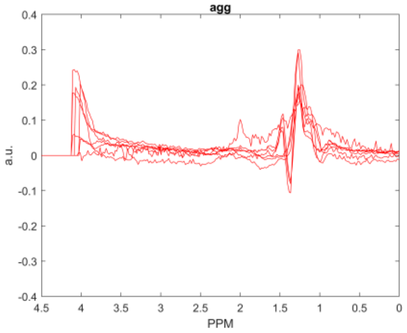

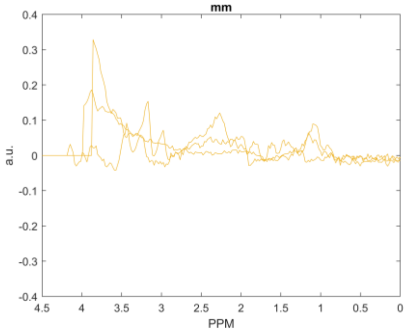

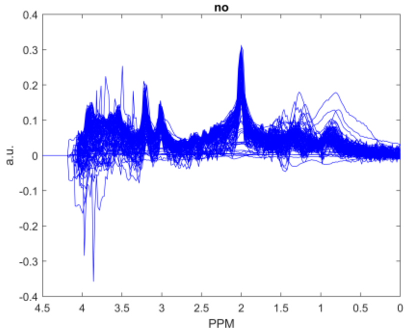

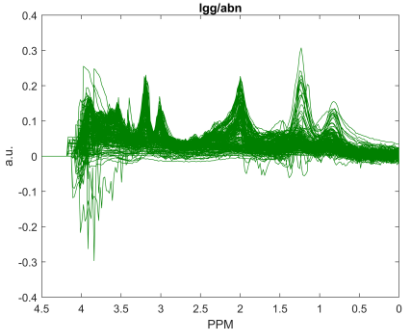

eT3401, S2(10x10)
Anaplastic Astrocytoma. BER=0.48, STI=Excluded

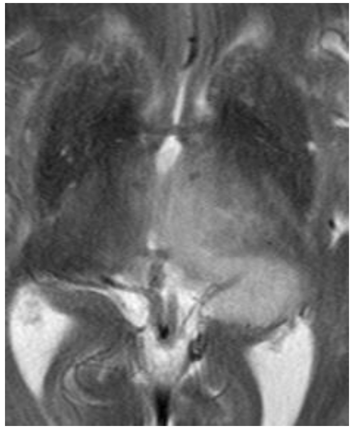

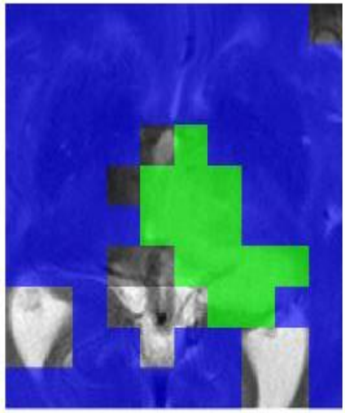

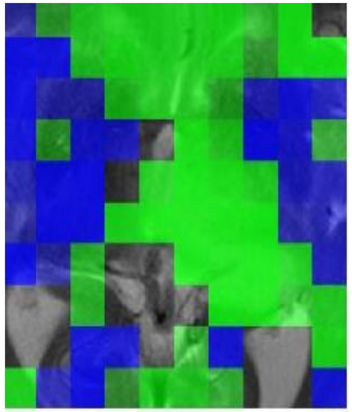

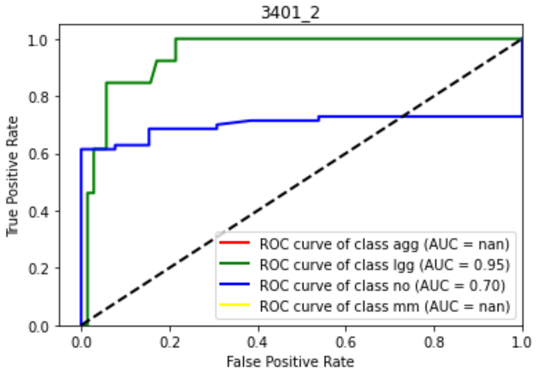

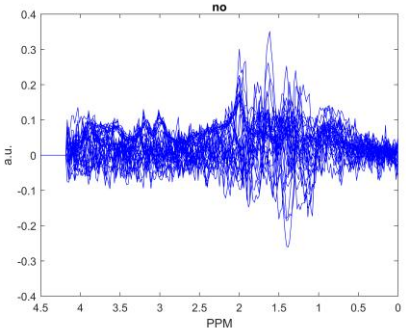

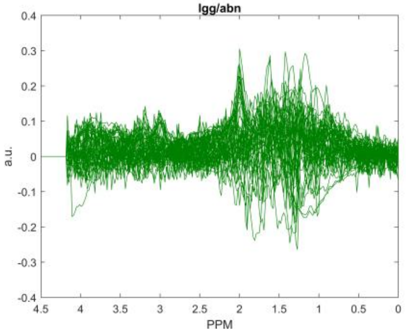

eT3401, S4(10x10)
Anaplastic Astrocytoma. BER=0.26, STI=Excluded

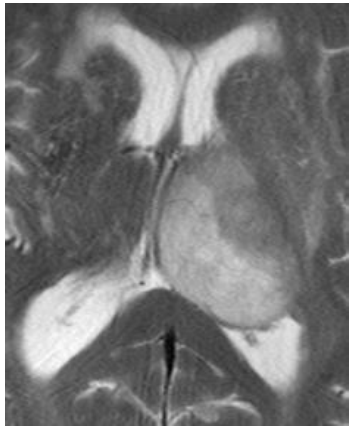

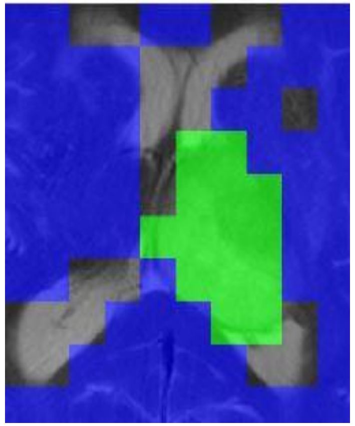

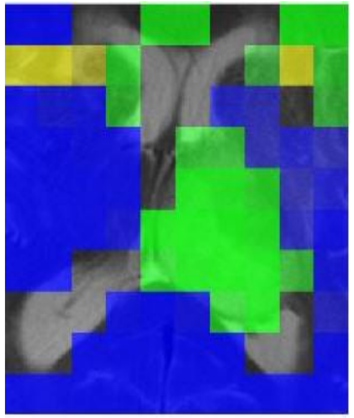

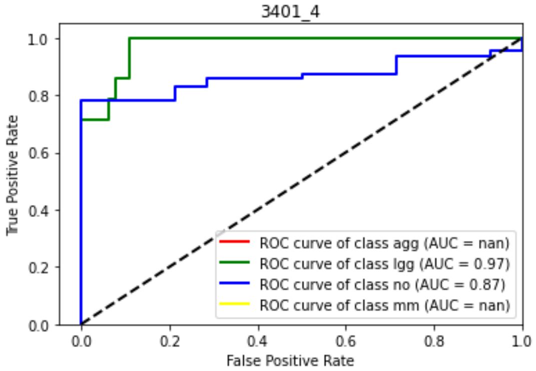

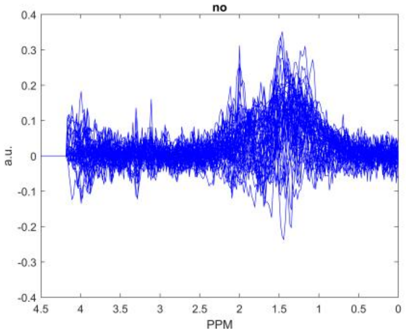

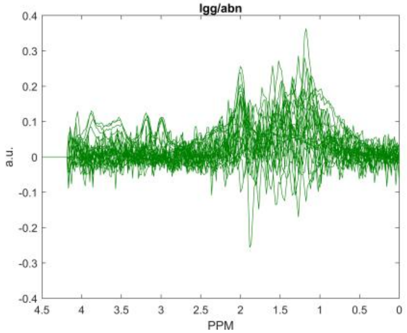

eT3401, S5(10x10)
Anaplastic Astrocytoma. BER=0.33, STI=Excluded

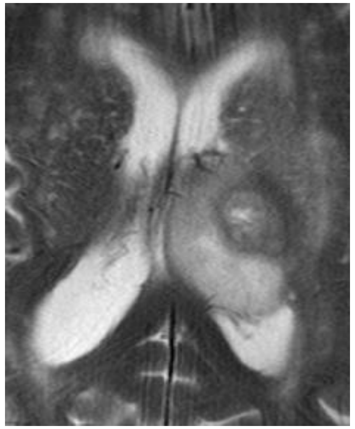

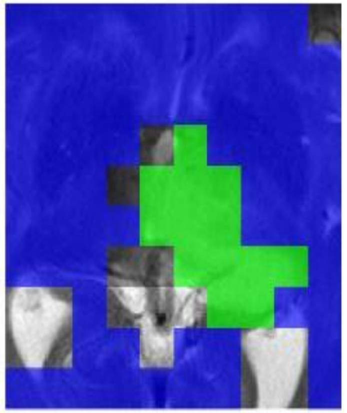

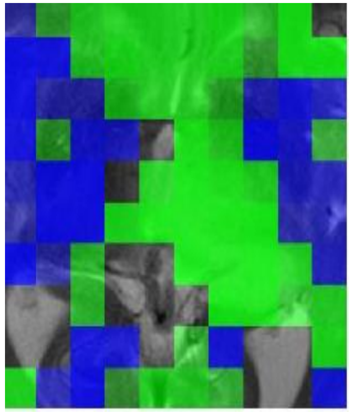

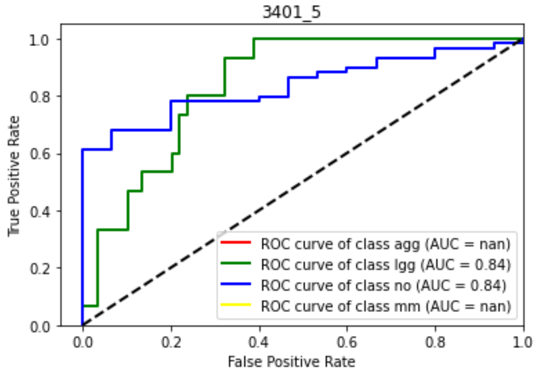

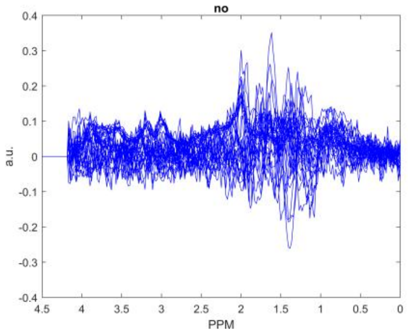

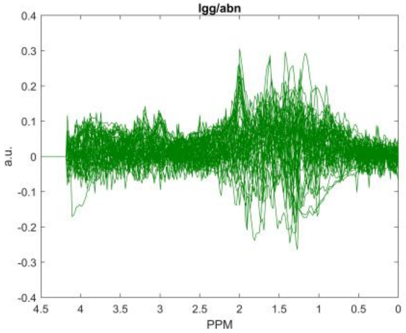

eT3401, S6(10x10)
Anaplastic Astrocytoma. BER=0.33, STI=Excluded

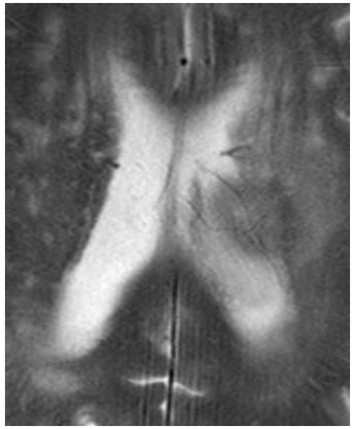

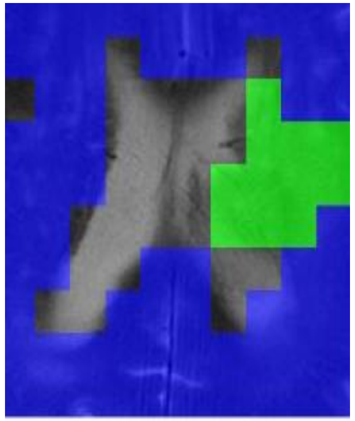

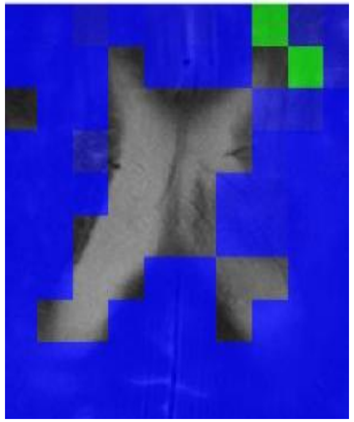

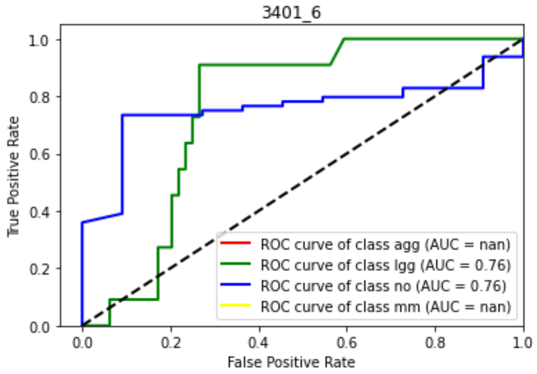

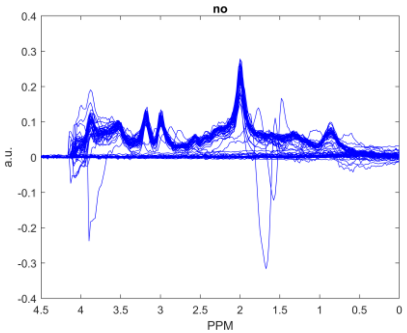

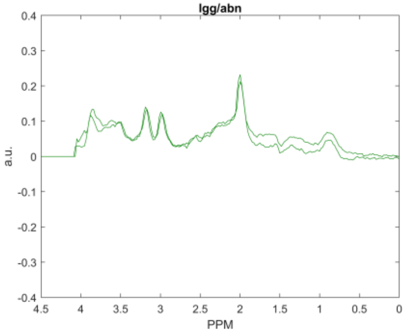

Supplement: Supplementary file 1 [file cancers-15-03709-s001.zip › Supplementary Materials C-20230720.pdf]
